# Integrated Solid/Solution NMR Assignment Enables Mapping of Dynamics and Ligand Binding in a 134 kDa Enzyme

**DOI:** 10.1021/jacs.6c14423

**Published:** 2026-10-13

**Authors:** Federico Napoli, Rajkumar Singh, Anna Kapitonova, Virgil Aitenbichler, Giorgia Toscano, Barbara Perrone, Paul Schanda

**Affiliations:** † 148492 Institute of Science and Technology Austria, Am Campus 1, A-3400 Klosterneuburg, Austria; ‡ Bruker Switzerland AG, CH-8117 Fällanden, Switzerland

## Abstract

Understanding enzyme function requires characterizing not only the static structure but also dynamics and ligand interactions. NMR spectroscopy provides this insight at atomic resolution, yet for large proteins, the difficulty of resonance assignment has largely confined such studies to systems below ∼50 kDa, or to observing only methyl groups. Here, we present an integrated magic-angle spinning (MAS) and solution NMR study of the 134 kDa tetrameric malate dehydrogenase from *Ignicoccus islandicus* (*Ii*MDH), an enzyme of particular interest as an evolutionary intermediate between allosteric lactate dehydrogenases and nonallosteric malate dehydrogenases. By combining high-dimensional (up to 4D) MAS NMR experiments on sedimented protein with solution NMR, we achieved 92% backbone heavy-atom assignment and 91% assignment of all Ile-δ_1_, Leu-δ_1_/-δ_2_, Val-γ_1_/-γ_2_, Met-ε, and Thr-γ methyl groups. Building on these assignments, we used various probes of backbone and side-chain dynamics: elevated MAS NMR ^15^N rotating-frame relaxation (*R*_1ρ_) points to slow motions in functionally critical regions, including the catalytic loop and the mobile surface loop. Complementary methyl-axis order parameters from solution NMR identified additional flexible sites in the hydrophobic core. Chemical shift perturbation experiments upon addition of the substrate analogue oxamate, monitored via backbone ^1^H–^15^N TROSY, revealed both active-site contacts and responses in helices α2F and α3G, regions associated with signal transmission in allosteric homologues. The integrated approach demonstrated here exploits the distinct strengths of MAS and solution NMR and provides a comprehensive view of structure, dynamics, and substrate interactions in a large oligomeric enzyme that neither technique could provide alone.

## Graphical abstract

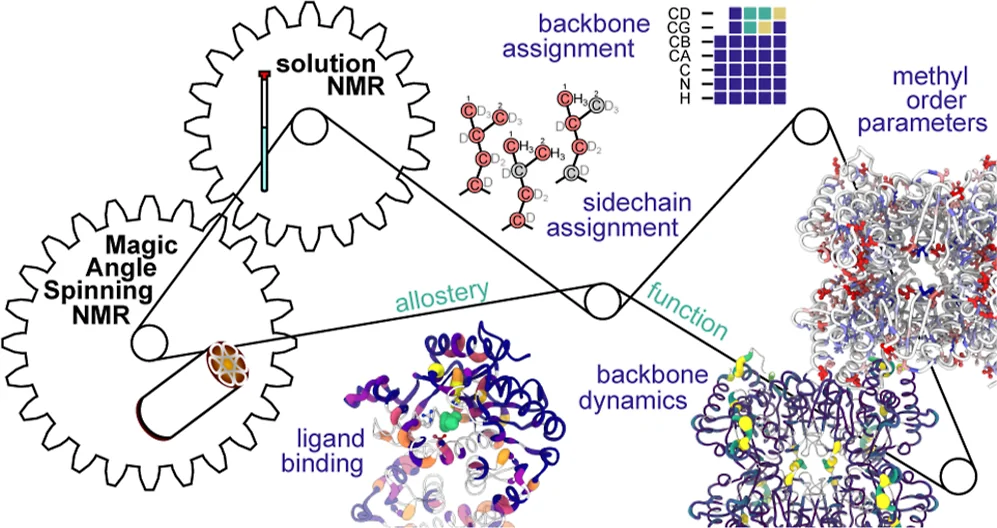

## 1 Introduction

Enzyme catalysis not only depends on a single “static” three-dimensional structure but also critically relies on the enzyme’s ability to sample alternate conformations that are transiently populated during the catalytic cycle or upon ligand binding.^1,2^ This is particularly evident in allosteric enzymes, where binding of a substrate or effector at one site modulates activity at a distant site through structural and dynamical changes that propagate across the protein. Understanding the mechanistic origins of allostery therefore requires characterizing not only the ground-state structure—where X-ray crystallography and cryo-EM excel—but also the dynamics and interactions of the protein at atomic resolution.^3,4^

The superfamily of malate and lactate dehydrogenases (MDHs and LDHs) offers a compelling system for studying the origins of allostery.^5,6^ LDHs and MDHs share the same fold and catalyze chemically equivalent reactions, yet LDHs exhibit allosteric regulation between their four subunits, while MDHs do not.^7^ The malate dehydrogenase from the archaeon *Ignicoccus islandicus* (*Ii*MDH) occupies a particularly interesting position in the phylogeny of this superfamily, sitting at the evolutionary intersection between allosteric LDHs and nonallosteric MDHs.^7^ As a 134 kDa homotetramer, *Ii*MDH catalyzes the reduction of oxaloacetate to malate, coupled to the oxidation of NADH to NAD^+^, within a conserved active site that brings the cofactor and substrate into close proximity. Interestingly, it also catalyzes the conversion of pyruvate to lactate—the reaction performed by LDHs—with signs of allosteric regulation, albeit at lower efficiency.^8^ These properties make *Ii*MDH an ideal model to dissect the structural and dynamical determinants of allostery at the atomic level.

Nuclear magnetic resonance (NMR) spectroscopy is uniquely suited to this endeavor: it reports on both the time-averaged structure and internal dynamics at the resolution of individual atoms, and it can probe proteins directly in solution, where transient interactions with substrates and cofactors are accessible.^9,10^ However, solution NMR has long faced an inherent size limitation because slow Brownian tumbling leads to rapid signal loss during the coherence-transfer steps of multidimensional experiments. TROSY-based backbone^11^ ^1^H–^15^N and methyl^12^ ^1^H–^13^C correlation experiments have substantially alleviated this problem. Methyl-TROSY approaches in particular have provided key functional insights into molecular machines approaching the megadalton range.^13,14^ Nonetheless, important practical limitations remain. First, methyl-TROSY experiments report exclusively on methyl-bearing side chains, leaving polar residues—which are often directly involved in active sites and allosteric interfaces—invisible. Second, and more fundamentally, while two-dimensional (2D) ^1^H–^15^N TROSY spectra can be obtained for large proteins, de novo resonance assignment usually requires 3D spectra with ^15^N–^13^C and ^13^C–^13^C coherence-transfer steps, but such experiments become prohibitively inefficient at high molecular weight. Common solutions to this problem fall into three categories:^15^ (i) in some cases, large proteins may be dissected into smaller domains, which can be assigned, and the assignment may subsequently be transferred to the spectra of the entire protein.^16–20^ The process is bound to the ability to track resonances from the small subdomains to the entire complex—despite the necessarily broken interfaces—and often requires extensive trial-and-error testing of constructs. (ii) As through-space polarization transfer (NOE) is efficient for large proteins, NOESY spectra along with the known 3D structure may also allow for resonance assignment; this technique is often used for methyl assignments.^21–25^ (iii) Alternatively, mutagenesis-based assignment uses a series of 2D ^1^H–^13^C spectra of single-point mutants, whereby the replacement of a methyl-bearing residue leads to the disappearance of a single cross peak.^20,26,27^ The approach is laborious; moreover, the mutations can lead to additional unwanted effects, such as peak shifts for nonmutated residues.

Magic-angle spinning solid-state NMR (MAS NMR) offers a complementary and, in several respects, advantageous approach. By replacing Brownian tumbling with mechanical sample rotation, MAS NMR removes the molecular-weight dependence of resolution and sensitivity, and proteins of hundreds of kilodaltons—frequently in oligomeric assemblies—have been studied by MAS NMR to reveal their structures, dynamics, and interactions.^28–30^ Large protein assemblies can be converted into a suitable “sedimented” solid-state sample by ultracentrifugation^31–33^ while remaining highly hydrated (typically more than 50% water by volume) and retaining the structure and dynamics closely similar to those in solution. This makes sedimented samples well-suited for backbone resonance assignment and for measurements of site-specific dynamics across a wide range of time scales.^34,35^ However, the study of transient intermolecular interactions—such as substrate binding to an enzyme—is less straightforward in sedimented samples.

Here, we exploit the complementary strengths of MAS and solution NMR to obtain a comprehensive view of the structure, dynamics, and substrate interactions of *Ii*MDH. We show that high-dimensional MAS NMR experiments on the sedimented protein yield a near-complete backbone resonance assignment, which is then transferred to the methyl groups through a series of solution and MAS NMR experiments, allowing for the unambiguous assignment of 91.3% of Ile-δ_1_, Leu-δ_1_/-δ_2_, Val-γ_1_/-γ_2_, Met-ε, and Thr-γ methyl groups. Through automated assignment runs with subsets of data, we identify which experiment types are most useful for assignment. The combined assignment enables several parallel lines of investigation of dynamics: ^15^N rotating-frame relaxation (*R*_1ρ_) measurements by MAS NMR are used to probe slow-time scale backbone dynamics; methyl-directed order parameter measurements and rotamer-equilibrium assessments in solution provide additional insights into side chain mobility. Backbone ^1^H–^15^N TROSY spectra in solution—assigned through the MAS NMR data—are used to probe millisecond motions and to map the structural response to the substrate analogue oxamate across the structure. Together, these results demonstrate that the integrated MAS/solution NMR strategy presented here is a powerful route to studying dynamics and interactions in large oligomeric proteins that are inaccessible to either technique alone.

## 2 Results and Discussion

### 2.1 High-Resolution MAS and Solution NMR of the *Ii*MDH Enzyme

Figure 1a–c compares TROSY-based solution-state 2D ^1^H–^15^N and 3D HNCO and HNCA spectra of deuterated (U–^2^H,^13^C,^15^N) *Ii*MDH with equivalent spectra recorded by MAS NMR techniques on a sedimented protein. The first important observation from this comparison is that the cross-peak positions are very similar in solution and in the sediment, mirroring previous reports.^32,36^ This establishes that data from both approaches can be combined and complement each other. Crucially, the number of detectable cross-peaks in solution is significantly lower than that in MAS, particularly in the 3D experiments, in which the signal is lost during the ^15^N–^13^C coherence-transfer delays. This signal loss is even more pronounced in experiments involving additional ^13^C–^13^C transfer steps, such as HNcoCACB or HNCACB, such that these crucial experiments for the assignment do not provide any useful information for the *Ii*MDH case (data not shown). In contrast, in the sedimented sample, excellent spectral quality was found in numerous high-dimensional experiments, establishing MAS NMR as the way forward for the assignment of the backbone and the side-chain heavy atoms.

**Figure 1:**
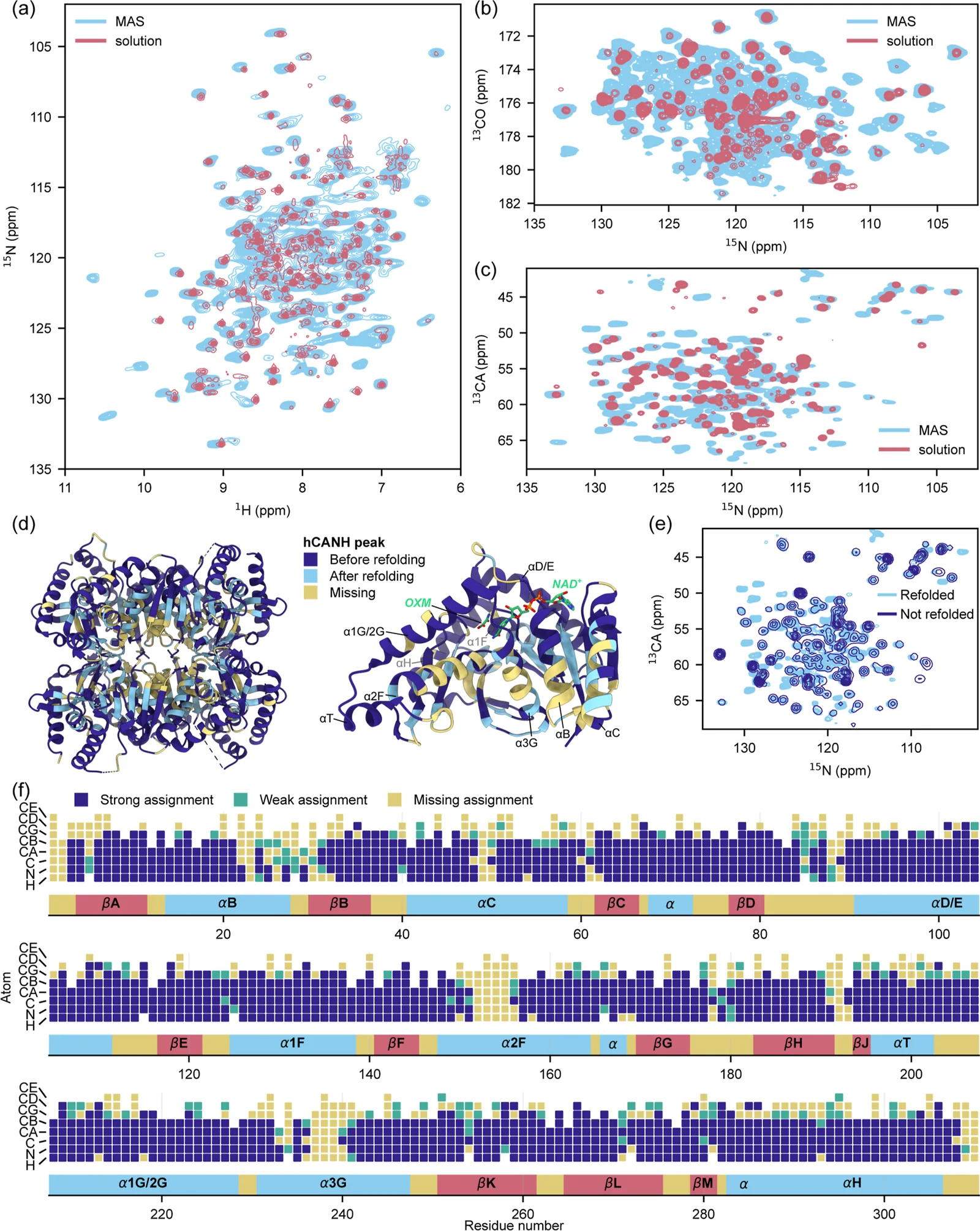
High-resolution solution and MAS NMR of *Ii*MDH. (a) Overlay of solution and MAS NMR 2D HN spectra. Despite the broader line-widths, the MAS NMR spectrum shows more peaks than the solution one. (b) Overlay of the N–CO projection of HNCO (solution) and hCONH (MAS) spectra. (c) Overlay of the N-CA projection of HNCA (solution) and hCANH (MAS). The difference in detectable peaks is exacerbated in 3D spectra, highlighting the limits of solution NMR for backbone assignment of large proteins. (d) Cartoon representation of the *Ii*MDH structure (PDB: 6QSS), highlighting residues that are visible before refolding, only after, or missing. Left: tetramer view; right: monomer view, with putative ligand binding sites—oxamate (OXM) and NAD^+^ aligned from *Thermus thermophilus* LDH (PDB: 2V7P). (e) Overlay of the hCANH spectra before and after refolding. Different contours are plotted for visual clarity. (f) Extent of MAS NMR assignment. Weak assignments are supported by a single peak; strong assignments are supported by two or more. Extended version and statistics are provided in Figure S3.

### 2.2 Assignment of Backbone and Side-Chain Heavy Atoms from MAS NMR

Our assignment approach followed two complementary strategies, using either ^13^C-detected experiments with U–^13^C,^15^N labeled protein (4D CANCOCX and CONCACX, recorded with a CPMAS CryoProbe;^37^ projections are provided in Figure S1) or amide-^1^H-detected experiments with the U–^2^H,^13^C,^15^N sample (3D hCANH, hCONH, hcaCBcaNH, and hNcocaNH and 4D hcaCBCANH, hcaCBcaCONH, hCACONH, hCOCANH, and hNCAcoNH). For the case of deuterated protein, we observed that the reprotonation of amide sites was incomplete, as commonly observed for highly stable proteins.^38–41^ We used an unfolding/refolding procedure to also exchange those amide sites that are buried in the folded state, which made 59 additional signals observable (Figure 1d,e; for experimental details, see the Materials and Methods section). We ensured that the refolded protein is structurally and functionally intact by comparing the protein before and after refolding in terms of chemical shifts, size-exclusion chromatography multiangle laser light scattering (SEC-MALS), mass photometry, and activity (Figure S2). Collectively, the complementary ^1^H– and ^13^C– detected experiments provided rich information for resonance assignment, with a total of 3849 manually picked cross-peaks for this 310-residue protein, listed in Table S1.

For the assignment, we opted for an iterative procedure of the program FLYA^42^—which uses a genetic algorithm for resonance assignment—and manual verification. This process turned out to be very efficient and resulted in the assignment of 92% of the backbone heavy atoms (C^α^, C′, N), 88% of the amide hydrogens (H^N^), and 55% of the side chain carbons (Figure 1f and extended in Figure S3). Overall, we obtained robust assignments—i.e., at least three out of four backbone atoms could be assigned—for 276 out of the 310 residues; the sequence stretches with missing or weak assignments include termini and loop regions (such as residues 1–2, 88–89, 191–193, 308–310), which is a frequently reported observation,^28,36,43,44^ related to fast local dynamics that renders the dipolar-coupling-based transfers less efficient.^45^ More interestingly, a continuous structural segment that extends from the tetramer center to helix α2F (which bears the substrate-complexing residue Arg154) also resisted the assignment efforts (Figure 1d). This observation is compatible with the presence of slow conformational dynamics in these regions, a hypothesis supported by CPMG experiments presented below. The residue-wise secondary structure positions, derived from these assigned chemical shifts using the TALOS-N approach,^46^ are in excellent agreement with those found in the crystal structure (Figure S4).

To assess the individual and cumulative contributions of the different spectra to the assignment, we performed ablation and addition studies with FLYA, presented in Figures S5 and S6. We found that FLYA was able to retrieve more than 90% of our backbone assignment in a fully automated manner and over 80% for the C^β^ assignments. Moreover, we found that ablation of single spectra or different classes (3D or 4D ^1^H-detected spectra, ^13^C-detected spectra) resulted in no or very modest deterioration of the results, suggesting a high degree of redundancy in the input data. Addition studies helped identifying two smaller sets of experiments that could result in satisfactory assignment: first, a combination of CANCOCX, CONCACX, hNCAcoNH, hcaCBcaCONH, and hcaCBCANH (with a combined duration of 803 h); second, in the absence of ^13^C-detection instrumentation, hCOCANH, hcaCBCANH, hNCAcoNH, and hcaCBcaCONH (657 h), see Figure S6d. A detailed description of the workflow is presented in Section S1.

With this near-complete backbone assignment from the sediment, we used two approaches for transferring the assignments to the solution NMR sample: matches of frequency triples in TROSY-based HNCO and HNCA backbone-assignment experiments—the only ones that provided a decent number of cross-peaks in solution—allowed identifying the majority of amide sites observed in the solution NMR TROSY spectrum; furthermore, a nuclear Overhauser effect spectrum (NOESY) connecting the amide sites confirmed and extended these assignments (Figure S7).

### 2.3 Leveraging MAS NMR Assignments for Complete Assignment of Methyl Groups in Solution

We aimed to obtain a comprehensive assignment of Met, Ile, Thr, Val, and Leu methyl sites in solution, with the goal of subsequently probing dynamics and interactions. The near-complete backbone and substantial side-chain ^13^C assignment from MAS NMR described above allows for direct spectroscopic methods for methyl ^1^H–^13^C assignment. We combined several solution- and MAS NMR experiments and studied their relative contributions to the assignment, as described in the following. In addition to these purely spectroscopic approaches, we prepared four single-point mutants (V27A, I50V, T152S, and V168A), out of a total of 115 methyl-harboring residues (M, I, L, V, T).

We compared five spectroscopic approaches—two through-bond experiments in solution, one through-bond in the solid state, and two NOESY-based—differing in labeling scheme, sensitivity, and residue coverage. The first approach employed solution NMR and a recently proposed isotope-labeling pattern of α-ketoisovalerate, which results in the incorporation of ^13^C in the two methyl groups (γ1, γ2 in Val; δ1, δ2 in Leu) and at the α (Leu and Val) and β (Leu) positions; the carbon next to the methyls is ^12^C, and therefore, the methyl carbons do not have one-bond ^13^C–^13^C couplings, which results in well-resolved singlet peaks. A specifically designed pulse sequence in solution connects both methyl groups to the C^α^ (Val) and C^α^/C^β^ (Leu) frequencies.^47^ Because the latter frequencies are available in our case from the MAS NMR assignment, this approach immediately identifies both methyl groups for residues with known C^α^/C^β^ assignments (Figure 2a, lines labeled HMBC-CC-HMQC; for this experiment, the V168A mutant was used). This approach allowed the assignment of 83 (71.6%) Val and Leu methyls. The entire assigned spectrum is shown in Figure S8.

**Figure 2:**
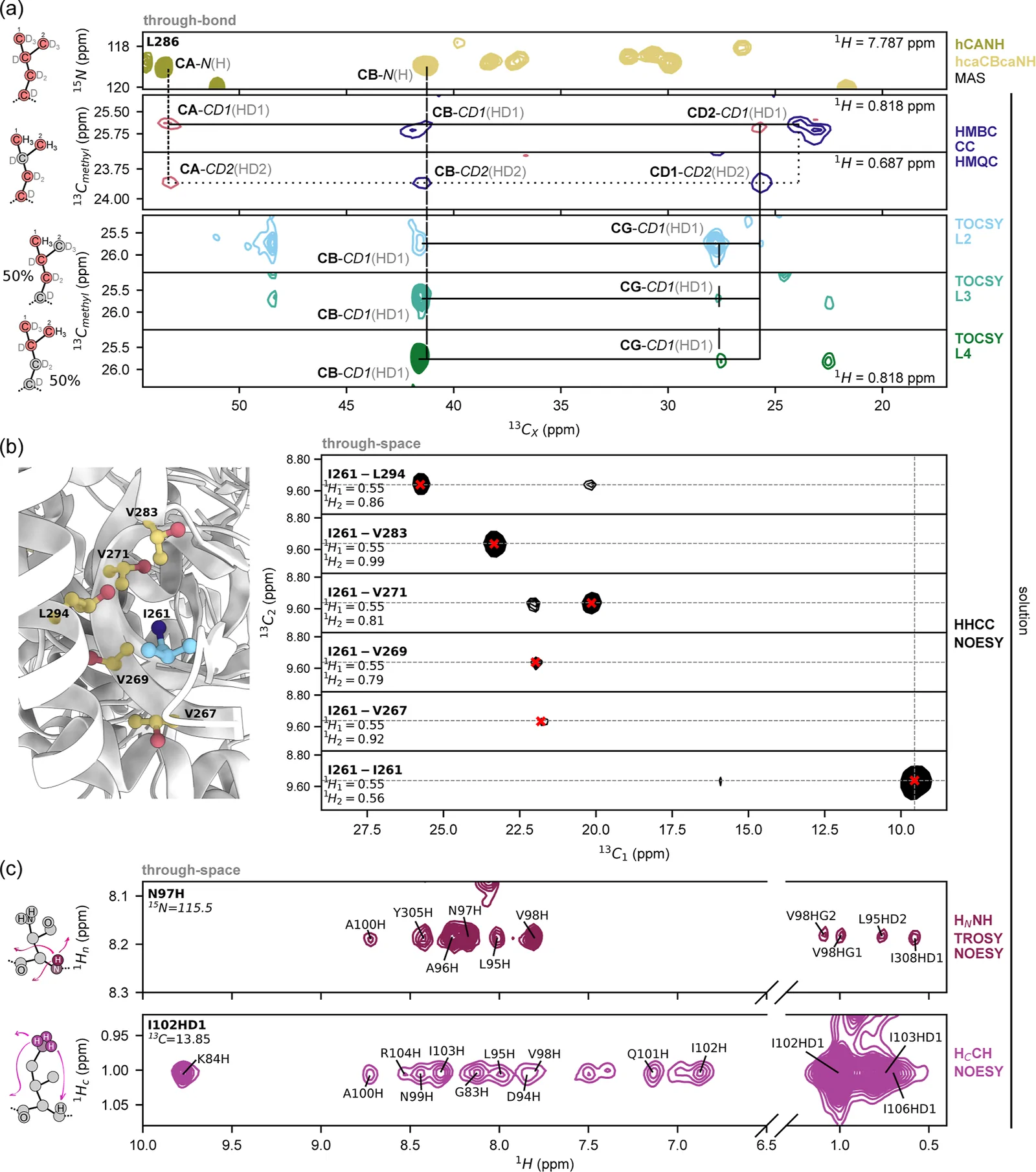
Solution NMR methyl assignment enabled by MAS NMR-derived assignment. (a) Through-bond experiments for Leu286 side-chain assignment in MAS and solution NMR. The different labeling schemes used, reported on the left, induce a mild isotope shift and changes in peak positions. From top to bottom: MAS NMR hCANH and hcaCBcaNH, solution NMR HMBC–CC-HMQC of a sample labeled with α-ketoisovalerate^47^ (both the slices corresponding to the two geminal methyl groups are shown), and solution NMR out-and-back TOCSY of a sample labeled with ketobutyrate and acetolactate precursors^48^ (three TOCSY mixing times—L2, L3, L4—resulted in different extents of transfer, favoring directly bonded or further carbons). (b) Through-space methyl–methyl contacts of Ile261 detected in solution NMR 4D H–H–C–C NOESY. The corresponding region in the crystal structure (PDB: 6QSS) is provided on the left. (c) Through-space backbone–methyl (top, H^N^-(TROSY)–N–H NOESY, Asn97) and methyl–backbone (bottom, H^C^–C-H NOESY, Ile102) contacts by solution NMR.

A second approach employed a labeling pattern that results in two kinds of molecules with linear ^13^C spin systems and deuteration everywhere except at a single methyl group:^48^ one pattern connects the methyl to the backbone (Val: γ1 → β → α; Leu: δ1 → γ → β; Ile: δ1 → γ1 → β → α), while the other connects the two methyls to each other (Val: γ2 → β → γ1; Leu δ2 → γ → δ1). Using a ^13^C-TOCSY-based pulse sequence in solution allowed connecting the methyl to the ^13^C frequencies along the side chain up to C^α^ (Val, Ile) or C^β^ (Leu, Figure 2a, lines labeled TOCSY). The duration of the TOCSY mixing resulted in different extents of transfer, favoring either the directly bonded or further remote carbons (labeled L2, L3, L4 in Figures 2a and S9; see the Materials and Methods section for details). Compared to the labeling pattern with alternate ^13^C spins described above, this experiment suffers from one-bond ^13^C–^13^C J-couplings, resulting in broader ^13^C lines (note that virtual homodecoupling is able to eliminate these splittings but was not employed here). This second approach resulted in 72 Ile, Val, and Leu methyls assigned (48.6%). Overall, these TOCSY experiments performed worse than HMBC–CC-HMQC at assigning Val and Leu residues—especially Val-γ2 and Leu-δ2 groups—but they nicely complemented it with the Ile assignments (which could not be obtained with the α-ketoisovalerate-labeled sample). The combination of the two strategies allowed the assignment of 117 Ile, Leu, and Val methyl groups (79.1%, Figure S10).

As a third approach, we implemented MAS NMR experiments to connect the methyl to the rest of the side chain, in a 3D H^methyl^–C^methyl^–C correlation experiment, or to the backbone amide, in a C^methyl^–N^amide^–H^amide^ experiment, respectively (see Figure S11). The former experiment (hCCH) yielded 98 assigned cross-peaks (44 methyl groups, 29.7%) and is, in principle, a valuable strategy for methyl assignment. The latter experiment (hCccNH) had lower sensitivity and resulted in only 22 assignable peaks, presumably because the magnetization is spread along the side chain carbons, and the final ^13^C–^15^N step primarily selects the magnetization at the C^α^. Thus, while the connection of the methyl to the backbone is also possible in the solid state, we found the solution NMR approaches described above advantageous: not only is the transfer efficiency higher, but the resolution of the ^1^H–^13^C spectrum is also superior.

The experiments described above are not able to report on the methyls of Thr and Met. As a fourth approach, we used several through-space NOESY experiments to also cover these, and to further support and extend the assignments obtained with the through-bond correlation experiments. 4D methyl NOESY (Figure S12) of an ILV-labeled sample, exemplified in Figure 2b for I261, was found to be particularly powerful at generating unambiguous through-space connectivities and was complemented by 3D H–H–C NOESY and 3D H–C–C NOESY of a MIT-labeled sample (Figure S13). The combination of these two 3D experiments contributes to overcoming the assignment ambiguity, and it should be noted that their sensitivity is clearly superior to that of the 4D, in particular for the H–H–C spectrum. The three spectra yielded a total of 295 nondiagonal peaks, resulting in the assignment of 99 Ile, Val, Leu, Met, Thr methyl groups (57.2%).

The last approach leveraged the amide H–N assignments to connect methyl groups to the backbone via two complementary experiments: an amide-resolved H^N^-(TROSY)–N–H NOESY experiment^49^ and a methyl-resolved H^C^–C–H NOESY counterpart (Figures 2c, S7, and S14). Due to the high degeneracy of amide and methyl ^1^H frequencies, these spectra are laborious to work with, but they nevertheless allowed the assignment of 70 Ile, Leu, and Val methyl groups (47.3%).

Collectively, these experiments—and the four single-point mutants that were required to resolve ambiguities—enabled the assignment of 100% of Ile-δ_1_, Leu-δ_1_/-δ_2_, Val-γ_2_, Met-ε, and Thr-γ and 63.4% of Val-γ_1_ (Figure S15). The extensive data set also allows us to evaluate the relative importance of the different approaches (the percentages of assignable methyl groups per spectrum are presented in Table S1). Solution NMR proved more efficient than the MAS experiments for methyl assignment, largely due to the better resolved methyl spectra. The HMBC-CC-HMQC experiment was particularly powerful for Leu/Val assignments (both geminal methyls), while the TOCSYs allowed the assignment of the majority of the Ile signals. NOESY spectra completed the picture and were the only spectroscopic source of assignment for Met and Thr residues: while four-dimensional experiments remain preferable to obtain unambiguous assignment of contacts, three-dimensional H–H–C, H–H–N and H–C–C NOESY spectra allow greater sensitivity and can represent a practical alternative when used in combination.

### 2.4 Backbone Dynamics of Key Functional Regions Identified by MAS NMR

Spin relaxation experiments in MAS NMR are sensitive to internal protein dynamics on a wide range of time scales.^50,51^ While in solution, the overall tumbling dominates spin relaxation, and the site-to-site variations are generally very small, in the solid state, spin-relaxation rate constants across the protein frequently differ by more than 1 order of magnitude. We focused on ^15^N rotating-frame relaxation (*R*_1ρ_), which is particularly sensitive to motions on time scales from hundreds of nanoseconds to the low millisecond regime, with the largest *R*_1ρ_ rate constants occurring for μs time-scale dynamics, depending also on the motional amplitude.^35,51–54^ Given the large size of the protein and associated spectral overlap in 2D spectra, we employed a pseudo-4D experiment consisting of a series of 3D hCANH spectra to resolve as many residues as possible; the high sensitivity of this experiment with a 1.9 mm rotor, combined with nonuniform sampling, allowed recording a full relaxation series of 7 spectra within 4 days. Figure 3a,c shows the *R*_1ρ_ rate constants in sedimented *Ii*MDH, and relaxation decay traces are presented in Figure S16. Determining the time scale and amplitude precisely would require measurements at several spin-lock RF field strengths and/or MAS frequencies. In the present study, we have recorded the *R*_1ρ_ data at a single condition (*ν*_MAS_ = 38 kHz, ν_RF_ = 10 kHz), which precludes the determination of the precise time scale. Elevated *R*_1ρ_ rate constants are found at several functionally interesting sites. The so-called catalytic loop, which harbors the catalytic histidine His178, is one of these regions; interestingly, a three-residue stretch comprising His178 itself was not detectable in these relaxation experiments; the low intensity of these signals is potentially due to the strong relaxation induced by slow dynamics. Residues 85–92, forming the “mobile surface loop”, also feature enhanced *R*_1ρ_ relaxation; this part is thought to close upon ligand binding, as shown by previous crystal structures of homologues.^55^ The segment with the most pronounced slow motion—as seen by elevated *R*_1ρ_—is the so-called Mobile Region 2 (MR2), which connects helices αT and α1*G*/2G. This region is associated with structural reorganizations between active and inactive states in allosteric lactate dehydrogenases,^56,57^ but not in nonallosteric malate dehydrogenases. Together with the observation of unassigned stretches in α3G and α2F, harboring the substrate-chelating Arg154, this finding suggests that the active site of *Ii*MDH explores multiple conformations and is not in a static activated one. Lastly, elevated *R*_1ρ_ rates are found for the N- and C-termini, which are expected to be flexible.

**Figure 3:**
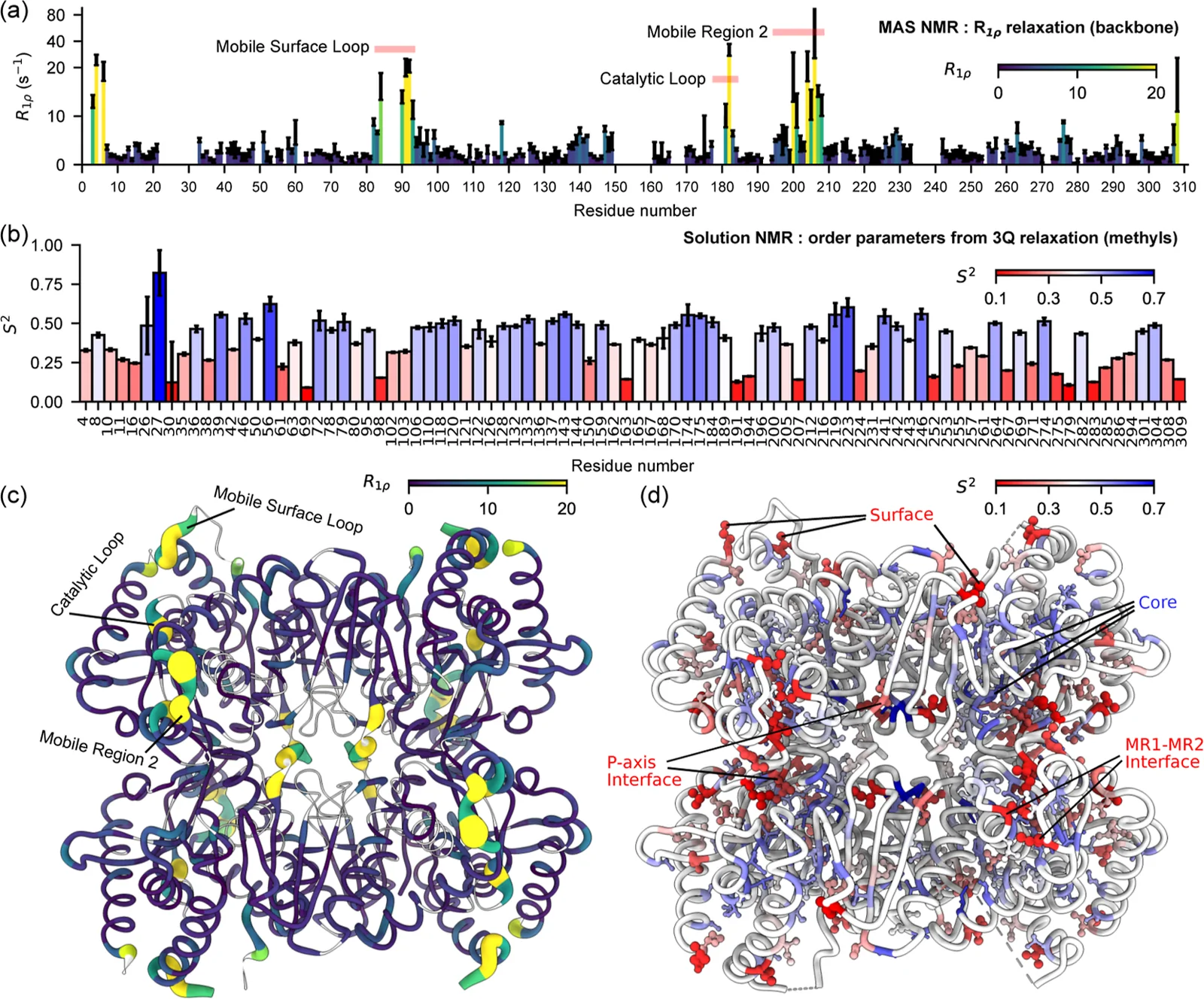
Site-specific insights into the dynamics of *Ii*MDH from backbone-directed MAS NMR and methyl-directed solution NMR. (a,c) ^15^N *R*_1ρ_ rate constants acquired at 38 kHz MAS frequency and a spin-lock radio frequency (RF) field strength of 10 kHz. (b,d) Methyl-axis order parameters, *S* ^2^, determined with a triple-quantum relaxation violated coherence transfer experiment.^59^

Collectively, backbone dynamics measurements in the solid state identify slow motions in regions of functional importance.

### 2.5 Solution-State ^15^N Relaxation–Dispersion NMR Points to Millisecond Motions

We have also attempted to complement these data with backbone amide Carr–Purcell–Meiboom–Gill (CPMG) relaxation–dispersion (RD) experiments in solution, using a TROSY-based sequence. This experiment probes the ^15^N transverse relaxation (*R*_2_) as a function of the frequency at which refocusing pulses are applied. Although such *R*_2_-type experiments are possible in the solid state,^58^ they are difficult because they are plagued by rapid coherence decay;^51^ thus, we explored ^15^N CPMG RD in solution. As outlined in the Introduction section, the sensitivity of backbone-detected experiments for a 134 kDa protein is limited; nonetheless, we have obtained data for 190 residues, which provide evidence for millisecond dynamics particularly in helices α1F, α1*G*/2G, α3G and Mobile Region 1 (MR1), located at the end of helix α2F^56^ (see Figure S17). These parts surround helix α2F that harbors the key active-site residue Arg154. This observation lends support to the hypothesis that conformational exchange processes revealed by elevated *R*_1ρ_ in solids (which are predicted to result in enhanced line broadening, see above), are one reason behind some parts of this protein resisting the MAS NMR assignment. Additional reasons for unassigned residues are likely to exist, such as degeneracy of resonance frequencies (peak overlap) or inefficient coherence transfer, possibly by dynamic processes not seen in the ^15^N CPMG and *R*_1ρ_ data.

### 2.6 Methyl-Detected Dynamics in Solution Highlight Dynamic Residues, Including in the Core

We studied side-chain dynamics to gain complementary views of *Ii*MDH’s mobility. Not only do methyls sense different types of motions, but in the case of *Ii*MDH, methyls also expand the regions of the protein for which dynamical information is accessible: in some parts where the backbone remained unassigned, methyl probes are available (residues 26, 27, 30, 150, 159, 241, 242). Solution NMR methyl spectra are advantageous in terms of resolution for *Ii*MDH, which prompted us to use solution NMR triple-quantum relaxation experiments^59^ to quantify the order parameters of methyls, *S*_axis_^2^. This parameter describes the motional restriction of the methyl axis, ranging from 1 (fully restricted) to 0 (unrestricted motion), on time scales shorter than the overall tumbling (ca. 39 ns).

The obtained order parameters of Ile, Leu, and Val in *Ii*MDH follow expected overall trends, where surface-exposed methyl groups are more disordered than those in the hydrophobic core (Figures 3b,d and S18). However, there are notable exceptions that are deeply buried yet show high flexibility, such as Ile30, Ile61, Leu163, Ile191, Ile252, Ile255, Ile275, and Leu279 (Figure S19). Interestingly, these involve the point of contact between MR2 and MR1 and several residues along the *P*-axis dimer–dimer interface.

A quantitative analysis of *S*_axis_^2^ against solvent accessibility and local packing density, computed from the crystal structure for all 90 Ile, Leu, and Val residues, is provided in the Supporting Information (Section S2, Table S5). Solvent accessibility and packing density correlate with *S*_axis_^2^ only very moderately, with a Pearson correlation coefficient |*r*| ≈ 0.3 (*p* < 0.01). Surface-exposed residues tend to be more mobile, but within the buried residues, there is a wide range of mobility. In other words, a static structure is a poor predictor of flexibility, which underscores that measuring *S*_axis_^2^ experimentally remains indispensable, mirroring other observations.^60,61^

Low methyl-axis order parameters reflect the mobility of the backbone as well as the transitions between rotamer states, i.e., rotations around the side-chain dihedral angles *χ*_1_ and *χ*_2_. Chemical shifts provide an orthogonal view on the population of rotamer states.^62–65^ Given the extensive assignment we have obtained, including both Val/Leu proS and proR methyls, quantitative estimates of rotamer-state populations are readily available from our data (Figure S20). Interestingly, we observed a mild correlation between trans/gauche–/gauche+ populations and 3Q relaxation-derived *S* ^2^ for Leu (*R* = 0.677) and Ile (*R* = 0.580) residues, suggesting that rotamer equilibrium can partially contribute to the order parameters. On the other hand, we found only poor correlation for Val (*R* = 0.262) residues. Three factors could contribute to this: first, the time scales probed by chemical shifts (up to ms) and relaxation (≲40 ns) substantially differ. Consequently, excess motion seen by chemical shifts may occur on the time scale from tens of nanoseconds to milliseconds. Second, estimating Val rotamer populations is complicated, and it relies on maximizing the agreement with quantum chemical calculations using a genetic algorithm.^62^ Third, for Val in particular, its shorter side chain could cause the methyl groups to experience more of the backbone dynamics and, therefore, rotamer equilibrium to contribute less to the measured order parameters.

### 2.7 Backbone-Observed Solution NMR for Quantitative Ligand-Binding Studies

Solution methods are clearly at their best for probing intermolecular interactions. Following chemical-shift perturbations (CSPs) upon the addition of a substrate is a powerful way not only to identify the residues involved in the binding but also to quantify the interaction strength. However, methyl-directed experiments fall short of seeing interactions at polar residues and therefore often miss the residues directly involved in the active sites. We used H–N TROSY-HSQC experiments to probe the binding of oxamate, a structural analogue of pyruvate and a known competitive inhibitor.^66,67^ Using two-dimensional line shape analysis in NMR-TITAN^68^ we determined a *K*_d_ of 22.5 ± 1.4 mM (Figure S21).

Figure 4 shows spectra and residue-specific CSPs upon addition of oxamate to *Ii*MDH. It should be mentioned that the solution NMR backbone spectrum exhibits fewer peaks than the MAS NMR one, especially missing residues in the surroundings of the nonassigned regions, possibly due to conformational-exchange broadening. Some of the residues that are most affected by binding are in the catalytic loop harboring the catalytic His178, i.e., the loop that we showed to undergo slow motions (no methyl-harboring residue is present in this loop). Similarly, Thr230 also shows large CSPs; it is in direct contact with the substrate, located on Mobile Region 3 (MR3) between helices α1*G*/2G and α3G^69^.

**Figure 4:**
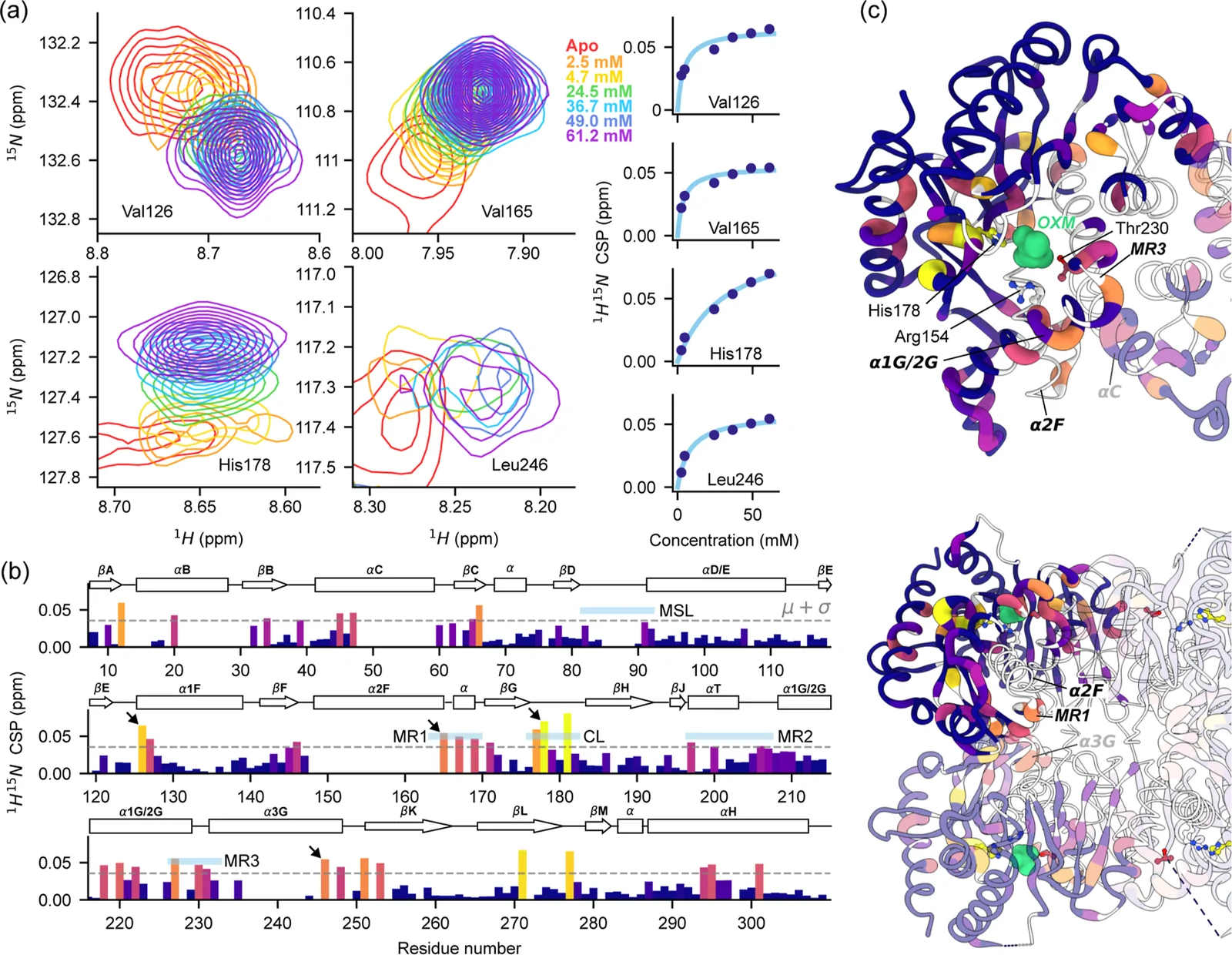
Backbone-observed oxamate binding in *Ii*MDH. (a) Concentration-dependent CSPs $\left(\Delta \delta_{NH}=\sqrt{{\left(\Delta \delta_{H}\right)}^{2}+{\left(0.14\Delta \delta_{N}\right)}^{2}}\right)$ upon oxamate binding for selected residues in *Ii*MDH. Left: overlay of 2D NH spectra recorded throughout the titration. Right: CSPs as a function of oxamate concentration. (b) Bar plot of CSPs in the presence of 61.2 mM oxamate. Large perturbations are observed for the catalytic loop (CL), MR1, MR3, and helix α3G. No significant changes $\left(\Delta \delta_{NH}>\overset{®}{\Delta \delta_{NH}}+\sigma_{\Delta \delta_{NH}}\right)$ are found at the mobile surface loop (MSL) and helix αD/E. Residues shown in panel (a) are highlighted with arrows. (c) Worm radius and color gradient representation of CSPs onto the *Ii*MDH structure (PDB: 6QSS). Top: top view; bottom: front view. The subunits are shown in different transparency for visual clarity.

While these effects are expected—given that these residues directly form the substrate-binding site—our data points to an interesting and less expected region: strong CSPs for Thr146, Thr148, Val165, Ile167, Ala169, and Asp171 are consistent with the hypothesis that helix α2F and MR1 may undergo a significant structural rearrangement. This helix bears residue Arg154, which is known to bind the substrate. Importantly, the Arg154–substrate interaction and the rearrangement of helix α2F/MR1 have been shown to be crucially involved in allosteric signal transmission.^6,56,70^ Moreover, the CSP data possibly suggests long-range structural modifications also in α3G: while on one side of this helix (Gln227, Thr230), the observed CSPs can be ascribed to direct interaction with oxamate, large CSPs are also seen for Leu246 and Asn248, located at its other extremity. This pattern of perturbations is compatible with structural coupling between the active site and the subunit interface. Interestingly, the end of helix α3G of one subunit is in direct contact with MR1 of the P-axis related subunit, creating a connection between active sites reminiscent of allosteric LDHs.^71^ As noted above, *Ii*MDH lies phylogenetically in between the nonallosteric MDH and allosteric LDH groups and has been proposed as a preallosteric enzyme.^8^ Moreover, this interface is surrounded by residues with low order parameters (see the previous section), which could allow motion between different conformations without engaging in strong hydrophobic interactions. At present, these interpretations should be regarded as tentative pending further experimental validation. Interestingly, we did not observe any significant perturbation $\left(\Delta \delta_{NH}>\overset{®}{\Delta \delta_{NH}}+\sigma_{\Delta \delta_{NH}}\right)$ for helix αD/E and the mobile surface loop.

Collectively, the availability of assigned backbone N–H resonances with N–H TROSY—which is still feasible for proteins up to hundreds of kDa—allowed probing substrate interactions, including polar residues that would escape detection by methyl-TROSY NMR.

## 3 Conclusions

Determining the functional mechanisms of enzymes requires going beyond static three-dimensional structures to characterize the dynamics and ligand interactions that underlie catalysis and allosterism. For large oligomeric proteins, this information has been difficult to obtain by NMR: de novo backbone assignment fails in solution at high molecular weight, methyl-TROSY approaches leave polar residues invisible, and the conventional mutagenesis-based route to methyl assignment is laborious. The present work shows that integrating MAS NMR of sedimented samples with solution NMR resolves these bottlenecks in a largely automated manner and opens up a comprehensive view of structure, dynamics, and interactions in a 134 kDa tetrameric enzyme.

The foundation of our approach is the backbone resonance assignment, which is achievable in the sedimented sample by MAS NMR but not in solution at this molecular weight. Near-complete assignment of backbone heavy atoms and amide protons was obtained by combining ^1^H- and ^13^C-detected high-dimensional experiments with iterative automated assignment using FLYA. Transferring these assignments to the solution-state sample through direct spectroscopic connections to the known backbone frequencies yielded 91.3% coverage of Ile, Leu, Val, Met, and Thr methyls—a level of completeness that would be difficult to achieve by mutagenesis alone for a protein of this size. Residues that resisted assignment are predominantly located in loop regions or at the tetrameric interface (including within secondary structure elements). While it is likely that at least some of the unassigned residues were missing because of overlap or chemical-shift degeneracy, it is interesting to note that some cluster in regions with elevated *R*_1ρ_, which points to slow dynamics.

With this assignment in hand, MAS and solution NMR each contributed distinct and complementary dynamical information. ^15^N *R*_1ρ_ relaxation measurements in the sedimented sample—resolving 244 individual sites in a series of 3D relaxation measurements—suggest the presence of pronounced slow-time scale backbone mobility in three functionally important regions: the catalytic loop harboring His178, the mobile surface loop (residues 85–92) that is expected to close upon substrate binding, and Mobile Region 2. An amide CPMG experiment further points to millisecond dynamics particularly at sites around helix α2F that harbors the active-site residue R154. Methyl-axis order parameters from solution NMR provided a complementary picture of side-chain dynamics, identifying additional flexible sites in the hydrophobic core—including deeply buried residues such as Ile30, Ile61, Leu163, Ile191, Ile252, Ile255, Ile275, and Leu279—that are not captured by backbone measurements alone. The two sets of dynamics measurements are thus not redundant: backbone *R*_1ρ_ is uniquely sensitive to slow motions and reports on the peptide plane, while methyl order parameters integrate pico- to nanosecond backbone and side-chain dihedral fluctuations.

The substrate analogue oxamate produced chemical shift perturbations across a wide network of backbone amide sites, detectable by ^1^H–^15^N TROSY in solution. Beyond the expected contacts at the active site—the catalytic loop and Thr230—significant perturbations were observed at helix α2F, which bears the substrate-chelating Arg154, and at the distal end of helix α3G. This distributed CSP pattern is compatible with structural coupling between the active site and the subunit interface. Some of these effects would have been invisible using methyl-TROSY alone, underscoring the value of backbone-level observation for mapping interaction networks in large proteins.

Taken together, these results establish a workflow for the NMR study of large oligomeric assemblies that integrates the strengths of both approaches: sedimented-sample MAS NMR for backbone assignment and dynamics and solution NMR for ligand interactions, side-chain assignment, and dynamics. In this study, we combined a large set of data from different samples, including ^1^H^13^C^15^N and refolded ^2^H^13^C^15^N proteins and ketoisovalerate- and acetolactate/ketobutyrate-labeled samples, to introduce multiple ^13^C sites in LV or ILV side-chains, respectively, and single-methyl ILV- and MIT-labeled proteins. Extensive assignment—simplified by relying on automated assignment tools—yielded an unusually complete set of probes, distributed across the backbone and side chains of five amino acid types. Nevertheless, this approach could be tailored to require substantially less data and still result in valuable coverage of backbone and side-chain frequencies: we have addressed this in the Supporting Information (Section S1, Table S1, and Figures S5, S6, and S10). For example, four ^1^H-detected MAS experiments for a refolded ^2^H^13^C^15^N sample combined with solution NMR TOCSYs on an acetolactate/ketobutyrate-labeled sample can result in ca. 80% backbone assignment (90% of what was achieved in this work, Figure S6d) and at least one methyl group assigned for more than 70% of Ile/Leu/Val residues (Figure S10). We anticipate that this strategy will be applicable to other large oligomeric proteins, when suitable isotope-labeling capabilities and high-field MAS and solution NMR instrumentation are available. It will be particularly powerful in cases, such as the MDH/LDH superfamily, where understanding function requires simultaneous access to dynamics at the active site, the subunit interfaces, and the regions connecting them.

## 4 Materials and Methods

### 4.1 Protein Production, Activity Measurements, and Size Characterization

A DNA insert coding for the sequence of *I. islandicus* malate dehydrogenase (uniprot ID A0A0U3FQH7) was cloned into a pET-20b vector between the NdeI and BamHI cloning sites (codon-optimized, obtained from GeneCust, https://genecust.org). Protein production was performed in *Escherichia coli* BL21 Star (DE3) in suitably prepared media for isotope labeling, as follows. For the production of fully protonated protein (used for ^13^C-detected experiments), the cells were grown in 100% H_2_O M9 medium, supplemented with 1 g L^–1^ ^15^N ammonium chloride and 2 g L^–1^ ^13^C_6_ glucose. For the production of deuterated proteins, cells were adapted to D_2_O in gradual steps (0, 50, 100%) of 8 h or overnight duration. All cultures were supplemented with 1 g L^–1^ ^15^N ammonium chloride and 2 g L^–1^ glucose, which was either ^13^C,1,2,3,4,5,6,6-^2^H_7_ labeled (MAS NMR samples and α-ketoisovalerate labeling) or 1,2,3,4,5,6,6-^2^H_7_ labeled (all the other side-chain labeling schemes). To produce the sample that was used for the HMBC–CC-HMQC approach, we used (2^13^C, 3-methyl-^13^C, 4^13^C, 3^2^H) α-ketoisovalerate^47^ (Mag-Lab, https://www.mag-lab.eu, compound L15), which was added to the culture 1 h prior to induction (150 mg L^–1^). For the samples with linearized ^13^C chains, used for the TOCSY experiments in solution and in solids, we followed the protocol by Kerfah et al.^48^ with some modifications: no alanine precursor was used; the glucose was deuterated, but not ^13^C labeled, which means that the α position of Leu is not ^13^C labeled in the final protein. We purchased linearized precursors for side-chain labeling in the form of a precursor kit (NMR-bio, https://nmr-bio.com). Acetolactate and ketobutyrate precursors were added 1 h or 20 min before induction, respectively. For the single-methyl labeling of Ile, Val, Leu (ILV sample) or Ile, Met, and Thr (MIT sample), used for NOESY experiments, we purchased TLAM-Iδ_1_LV-proS and TLAM-Iδ_1_MεTγ precursor kits (NMR-bio) and followed the manufacturer’s instructions.

Cultures were started at an OD_600_ of 0.2 and grown at 37 °C. Expression was induced when OD_600_ reached 0.6–0.8 and performed at 37 °C for 3–5 h or at 20 °C overnight. The buffers used in this study are listed in Table 1.

**Table 1:** Buffer Compositions

Buffer L	50 mM NaCl, 50 mM Tris–HCl, 0.025 mg mL^–1^ RNase, 0.05 mg mL^–1^ DNase, 2 mM MgCl_2_, protease inhibitor (complete, EDTA-free), pH 7
Buffer A	50 mM NaCl, 50 mM Tris–HCl, pH 7
Buffer B	1 M NaCl, 50 mM Tris–HCl, pH 7
Buffer T	50 mM NaCl, 50 mM MES, pH 6

Cells were harvested by centrifugation (15 min, 4000 rcf), and pellets were resuspended in Buffer L and lysed either by sonication, using a Q700 ultrasonic processor (Qsonica, 40% amplitude, 6 min operating time), or by high pressure (continuous high-pressure cell disruptor, 25–30 kpsi, 2 cycles). Initial purification was performed by heat shock of the lysates (75 °C, 20 min) before high-speed centrifugation (50,000 rcf, 60 min, 4 °C). The supernatant was filtered (0.22 μm pore size) before applying it on an anion exchange Resource Q column (GE Healthcare) pre-equilibrated with Buffer A. Protein was eluted with a linear gradient of Buffer B (10 column volumes, 60 mL). Protein-containing fractions were concentrated to <2 mL and applied on a Size Exclusion Chromatography HiLoad 26/600 Superdex 200 PG column (Cytiva/GE), pre-equilibrated with Buffer A.

For the unfolding/refolding procedure, the protein was diluted to 0.1 mg/mL in 6 M guanidinium chloride and incubated at 4 °C overnight, followed by a heat shock (85 °C, 1 h). The sample was allowed to cool down before dialysis with Buffer A in two steps. Lastly, the sample was centrifuged (20,000 rcf, 20 min) to remove aggregates and concentrated in Buffer A.

Conversion of oxaloacetate to malate was measured by following the oxidation of NADH (0.2 mM, ε: 6220 M^–1^ cm^–1^). We used a stopped-flow mixing system SFM-3000 (BioLogic) equipped with a FC-15 cuvette (1.5 mm path length, BioLogic) and monitored the absorbance of NADH at 340 nm. Measurements were performed at the highest achievable temperature (57 °C), in Buffer A at pH 7 (in line with previous activity measurements with oxaloacetate^8^). The reported specific activities were determined from triplicates of measurements.

Size exclusion chromatography coupled to multiangle light scattering (SEC-MALS) was used to determine the molecular weight of *Ii*MDH before and after refolding. Samples were prepared in Buffer A, at 2.0 mg mL^–1^. Before each run, samples were centrifuged for 10 min at 16,000 g, 4 °C. 30–40 μL of protein was injected onto a Superdex 200 5/150 (Cytiva) or Xbridge Premier Protein SEC 250 Å, 2.5 μm column (Waters), connected to a 1260 Infinity II HPLC (Agilent Technologies). A miniDAWN TREOS II (Wyatt Technology) and an Optilab T-REX (Wyatt Technology) were used to detect light scattering at 658 nm and measure refractive index, respectively. Runs were performed at room temperature, and the molecular weight was calculated with ASTRA software (Version 8.2.2.119, Waters/Wyatt Technology).

Similarly, molecular weight before and after refolding was also investigated by mass photometry. Measurements were conducted in Buffer A, at room temperature, using a Refeyn Two mass photometer (Refeyn Ltd.). Prior to each measurement, the instrument was focused using 10 μL of buffer, after which 1–2 μL of the sample was added directly to the measurement droplet (to a final concentration of 20–30 nM). Movies were collected for 60 s and analyzed using DiscoverMP software (Refeyn Ltd.). Calibrations were performed in the measurement buffer before each acquisition.

### 4.2 MAS NMR Spectroscopy

Samples for MAS NMR were prepared by ultracentrifugation of the protein solution (at ca. 15 mg/mL, H_2_O-based Buffer A) into MAS NMR rotors using an in-house built ultracentrifugation tool adapted for a Beckman SW32 rotor: a 1.9 mm rotor (Bruker BioSpin, part number H123832) was used for ^1^H-detected experiments (assignment, dynamics); a 3.2 mm rotor was used for the CPMAS CryoProbe (volume 81 μ L with an insert for wet samples) for the ^13^C-detected assignment experiments; and a 1.3 mm rotor was used for hCccNH and hCCH TOCSY experiments. The rotors were filled by overnight centrifugation at 20,000 rpm (68,300 g). Rotor caps were glued with two-component epoxy glue to avoid dehydration. Sample temperatures were determined on an external sample containing 25 mM TmDOTP and 10 mM DSS.^72^ Effective temperatures are not known for the hcaCBcaNH, CONCACX, and CANCOCX spectra as calibrations were not available for the two probes used for them.

All ^1^H-detected MAS NMR experiments were recorded on a Bruker BioSpin NEO spectrometer operating at a ^1^H frequency of 700 MHz, equipped with a 1.9 mm HXY MAS probe tuned to ^1^H, ^13^C, and ^15^N frequencies, except for a hcaCBcaNH spectrum recorded at 600 MHz (1.9 mm probe). All ^13^C-detected experiments were recorded on a 600 MHz Bruker NEO spectrometer equipped with a CPMAS CryoProbe.^37^ Sample labeling, rotor sizes, MAS speeds, pH, set and real temperature, nonuniform sampling percentages, and experimental times for the MAS spectra are reported in Table S3. All acquisition parameters (transfers, dimension settings, decoupling, etc.) are provided in Table S4.

Backbone assignment was achieved by multiple iterations of FLYA^42^ calculations and manual verification. Tolerances of 0.08–0.1 ppm and 0.4–0.55 ppm were used for ^1^H and ^13^C/^15^N, respectively. Each calculation consisted of 25–100 runs. Different combinations of input peak lists were tested, for example, separating ^1^H-detected and ^13^C-detected spectra, which are characterized by mild isotope shift effects deriving from the deuteration level (U-^2^H,^13^C,^15^N refolded for H-detected, and U–^1^H,^13^C,^15^N for C-detected). After the different runs, converged assignments (i.e., where the same assignment within tolerance was obtained in at least 80% of the runs) were validated manually, and the confirmed ones were used as inputs for future runs (shiftassign_statistics parameter, .prot format). Note that the nomenclature “weak” and “strong” in Figures 1 and S3 is related to the number of peaks supporting the assignment (“weak”, 1 peak; “strong”, more than one peak) and should not be confused with FLYA’s convergence nomenclature. All raw data, pulse sequences and FLYA runs are available, as described in the Data Availability section.

*R*_1ρ_ relaxation was measured in a series of 7 three-dimensional hCANH spectra with nonuniform sampling (60%) for a total duration of 4 days. The spinlock frequency was set to 10 kHz, and the relaxation delays were 5, 20, 40, 60, 80, 100, and 130 ms. Relaxation rates were determined by fitting a monoexponential function to the peak intensity decays.

### 4.3 Solution-State NMR Spectroscopy

Unless noted otherwise, solution-state NMR samples were prepared in H_2_O- or D_2_O-based Buffer A, supplemented with 3.75% D_2_O, 0.4 mM DSS (final concentrations) to enable lock and spectral referencing. All spectra were obtained on an 800 MHz Avance NEO spectrometer equipped with a TCI ^1^H/^13^C/^15^N 5 mm cryo probe. Sample labeling, protein concentrations, solvent, pH, temperature, and experimental times are reported in Table S2. All acquisition parameters (transfers, dimension settings, decoupling, etc.) are provided in Table S4. Oxamate titration was carried out in H_2_O-based Buffer T, where pH was set to 6, in line with pyruvate activity tests.^8^ Concentrations of samples for solution-state NMR are indicated in Table S2. The oxamate stock was prepared in the same buffer, and the pH was adjusted using NaOH. The effect of altering the ionic strength was investigated in a matched control in Figure S22.

Methyl-axis order parameters *S* ^2^ were determined through a 3Q-relaxation experiment,^59^ in two series of 8 two-dimensional CH experiments for a total duration of 109 h. Relaxation delays of 2, 4, 6, 8, 11, 15, 20, and 25 ms were used. The overall-tumbling correlation time was determined through the TRACT cross-correlated relaxation experiment.^73^ More details about these two experiments, fitting routines, and equations are provided in the Supporting Information. The consideration of anisotropic tumbling is also discussed there and compared to isotropic tumbling in Figure S18c. The solvent-accessible surface areas were calculated for the crystal structure (PDB: 6QSS) using the Python module FreeSASA.^74^ We also investigated potential systematic errors in the 3Q experiment due to external protons; this analysis, shown in Figure S23 and discussed in Section S2.4 of the Supporting Information, indicates that the accuracy of the *S*_axis_^2^ values is not significantly impacted by remote spins.

Binding affinity for oxamate was determined via two-dimensional line shape analysis in NMR-TITAN.^68^ Val126, Val165, and His178 were chosen due to their large CSP and their belonging to different structural elements (α1F, Mobile Region 1, and catalytic loop). Leu246 could not be fit due to its low intensity throughout the titration. We chose a simple two-state ligand binding model to fit the dissociation constant (*K*_d_, in μM) and rate (*k*_off_, in s^–1^). Protein (monomer basis) and ligand concentrations were explicitly given as inputs. Errors were estimated with the bootstrapping method (100 replicates).

## 5 Associated Content

### 5.1 Data Availability

Resonance assignments and assigned peak lists were deposited at the biological magnetic resonance data bank (BMRB ID 53910). All the raw spectra (Bruker format) used for assignment and dynamics measurements in this study, spectra and peak lists of the titration, all pulse sequences, analysis scripts, and the FLYA runs were deposited at ISTA’s Research Explorer repository^75^ (10.15479/AT-ISTA-22687).

## Supplementary Material



## Notes

Investigation of the relative importance of different experiments for assignment; discussion of the fitting details of 3Q data and correlation to structural parameters; tables with all acquisition parameters and spectral statistics; figures showing projections of multidimensional NMR spectra, comparisons of refolded and untreated protein integrity, secondary-structure analysis, assignment statistics, pulse sequences, relaxation analyses, and titration analyses (PDF)

## References

[1] Teptarakulkarn P. H., Treviño R. E., Hansen A. L., Laureanti J. A., Buchko G. W., Shaw W. J., Shafaat H. S. (2026). Correlating protein
dynamics and catalytic activity of a model hydrogenase using paramagnetic
and biological nuclear magnetic resonance spectroscopy. J. Am. Chem. Soc..

[2] Hunt S. E., Klaus C., John A. E., Zarifi N., Martinez A., Feixas F., Garcia-Borràs M., Thompson M. C., Chica R. A. (2025). Distal mutations
in a designed retro-aldolase alter
loop dynamics to shift and accelerate the rate-limiting step. J. Am. Chem. Soc..

[3] Motlagh H. N., Wrabl J. O., Li J., Hilser V. J. (2014). The ensemble nature
of allostery. Nature.

[4] Basner J. E., Schwartz S. D. (2005). How enzyme dynamics helps catalyze a reaction in atomic
detail: a transition path sampling study. J.
Am. Chem. Soc..

[5] Robin A. Y., Brochier-Armanet C., Bertrand Q., Barette C., Girard E., Madern D. (2023). Deciphering
evolutionary trajectories of lactate dehydrogenases
provides new insights into allostery. Mol. Biol.
Evol..

[6] Coquille S., Pereira C. S., Roche J., Santoni G., Engilberge S., Brochier-Armanet C., Girard E., Sterpone F., Madern D. (2025). Allostery
and evolution: A molecular journey through the structural and dynamical
landscape of an enzyme super family. Mol. Biol.
Evol..

[7] Brochier-Armanet C., Madern D. (2021). Phylogenetics and biochemistry elucidate
the evolutionary
link between L-malate and L-lactate dehydrogenases and disclose an
intermediate group of sequences with mix functional properties. Biochimie.

[8] Roche J., Girard E., Mas C., Madern D. (2019). The archaeal LDH-like
malate dehydrogenase from Ignicoccus islandicus displays dual substrate
recognition, hidden allostery and a non-canonical tetrameric oligomeric
organization. J. Struct. Biol..

[9] Harkness R. W., Zhao H., Toyama Y., Schuck P., Kay L. E. (2024). Exploring
host-guest interactions within a 600 kDa DegP protease cage complex
using hydrodynamics measurements and methyl-TROSY NMR. J. Am. Chem. Soc..

[10] Bonin J. P., Aramini J. M., Kay L. E. (2024). Structural plasticity as a driver
of the maturation of pro-interleukin-18. J.
Am. Chem. Soc..

[11] Pervushin K., Riek R., Wider G., Wüthrich K. (1997). Attenuated
T_2_ relaxation by mutual cancellation of dipole–dipole
coupling and chemical shift anisotropy indicates an avenue to NMR
structures of very large biological macromolecules in solution. Proc. Natl. Acad. Sci. U. S. A..

[12] Tugarinov V., Hwang P. M., Ollerenshaw J. E., Kay L. E. (2003). Cross-correlated
relaxation enhanced ^1^H-^13^C NMR spectroscopy
of methyl groups in very high molecular weight proteins and protein
complexes. J. Am. Chem. Soc..

[13] Rennella E., Huang R., Yu Z., Kay L. E. (2020). Exploring long-range
cooperativity in the 20S proteasome core particle from Thermoplasma
acidophilum using methyl-TROSY-based NMR. Proc.
Natl. Acad. Sci. U. S. A..

[14] Rosenzweig R., Kay L. E. (2014). Bringing dynamic molecular machines into focus by methyl-TROSY
NMR. Annu. Rev. Biochem..

[15] Frueh D. P. (2014). Practical
aspects of NMR signal assignment in larger and challenging proteins. Prog. Nucl. Magn. Reson. Spectrosc..

[16] Mahdi S., Semenova I. V. (2025). Divide-and-conquer strategy
for NMR studies
of the E. coli γ-clamp loader complex. J. Biomol. NMR.

[17] Xie T., Saleh T., Rossi P., Kalodimos C. G. (2020). Conformational
states dynamically populated by a kinase determine its function. Science.

[18] Schwieters C. D., Suh J.-Y., Grishaev A., Ghirlando R., Takayama Y., Clore G. M. (2010). Solution structure of the 128 kDa
enzyme I dimer from *Escherichia coli* and its 146 kDa complex with HPr using residual dipolar couplings
and small- and wide-angle X-ray scattering. J. Am. Chem. Soc..

[19] Gelis I., Bonvin A. M. J. J., Keramisanou D., Koukaki M., Gouridis G., Karamanou S., Economou A., Kalodimos C. G. (2007). Structural
basis for signal-sequence recognition by the translocase motor SecA
as determined by NMR. Cell.

[20] Sprangers R., Kay L. E. (2007). Quantitative dynamics and binding
studies of the 20S
proteasome by NMR. Nature.

[21] Chao F.-A., Shi L., Masterson L. R., Veglia G. (2012). FLAMEnGO: A fuzzy logic approach
for methyl group assignment using NOESY and paramagnetic relaxation
enhancement data. J. Magn. Reson..

[22] Chao F.-A., Kim J., Xia Y., Milligan M., Rowe N., Veglia G. (2014). FLAMEnGO 2.0:
An enhanced fuzzy logic algorithm for structure-based assignment of
methyl group resonances. J. Magn. Reson..

[23] Xu Y., Matthews S. (2013). MAP-XSII: An improved
program for the automatic assignment
of methyl resonances in large proteins. J. Biomol.
NMR.

[24] Monneau Y. R., Rossi P., Bhaumik A., Huang C., Jiang Y., Saleh T., Xie T., Xing Q., Kalodimos C. G. (2017). Automatic
methyl assignment in large proteins by the MAGIC algorithm. J. Biomol. NMR.

[25] Clay M. C., Saleh T., Kamatham S., Rossi P., Kalodimos C. G. (2022). Progress
toward automated methyl assignments for methyl-TROSY applications. Structure.

[26] Amero C., Durá M. A., Noirclerc-Savoye M., Perollier A., Gallet B., Plevin M. J., Vernet T., Franzetti B., Boisbouvier J. (2011). A systematic
mutagenesis-driven strategy for site-resolved
NMR studies of supramolecular assemblies. J.
Biomol. NMR.

[27] Shi L., Kay L. E. (2014). Tracing an allosteric
pathway regulating the activity
of the HslV protease. Proc. Natl. Acad. Sci.
U. S. A..

[28] Klein A., Rovó P., Sakhrani V. V., Wang Y., Holmes J. B., Liu V., Skowronek P., Kukuk L., Vasa S. K., Güntert P., Mueller L. J., Linser R. (2022). Atomic-resolution chemical characterization
of (2x) 72-kDa tryptophan synthase via four- and five-dimensional ^1^H-detected solid-state NMR. Proc. Natl.
Acad. Sci. U. S. A..

[29] Loquet A., Habenstein B., Lange A. (2013). Structural investigations of molecular
machines by solid-state NMR. Acc. Chem. Res..

[30] Gauto D. F., Macek P., Malinverni D., Fraga H., Paloni M., Sučec I., Hessel A., Bustamante J. P., Barducci A., Schanda P. (2022). Functional control of a 0.5 MDa TET
aminopeptidase by a flexible loop revealed by MAS NMR. Nat. Commun..

[31] Mainz A., Jehle S., van Rossum B. J., Oschkinat H., Reif B. (2009). Large protein complexes with extreme
rotational correlation times
investigated in solution by magic-angle-spinning NMR spectroscopy. J. Am. Chem. Soc..

[32] Bertini I., Luchinat C., Parigi G., Ravera E., Reif B., Turano P. (2011). Solid-state NMR of
proteins sedimented by ultracentrifugation. Proc. Natl. Acad. Sci. U. S. A..

[33] Bertini I., Engelke F., Luchinat C., Parigi G., Ravera E., Rosa C., Turano P. (2012). NMR properties of sedimented solutes. Phys. Chem. Chem. Phys..

[34] Rohaim A., Vermeulen B. J. A., Li J., Kümmerer F., Napoli F., Blachowicz L., Medeiros-Silva J., Roux B., Weingarth M. (2022). A distinct
mechanism of C-type inactivation
in the Kv-like KcsA mutant E71V. Nat. Commun..

[35] Napoli F., Becker L. M., Schanda P. (2023). Protein dynamics detected
by magic-angle
spinning relaxation dispersion NMR. Curr. Opin.
Struct. Biol..

[36] Gauto D. F. (2019). Integrated NMR and cryo-EM
atomic-resolution structure determination
of a half-megadalton enzyme complex. Nat. Commun..

[37] Hassan A. (2020). Sensitivity boosts by the CPMAS CryoProbe for challenging
biological
assemblies. J. Magn. Reson..

[38] Napoli F., Guan J.-Y., Arnaud C.-A., Macek P., Fraga H., Breyton C., Schanda P. (2024). Deuteration of proteins boosted by
cell lysates: high-resolution amide and H^α^ magic-angle-spinning
(MAS) NMR without the reprotonation bottleneck. Magn. Reson..

[39] Aucharova H., Klein A., Gomez S. M., Söldner B., Vasa S. K., Linser R. (2024). Protein deuteration
via algal amino
acids to circumvent proton back-exchange for ^1^H-detected
solid-state NMR. Chem. Commun..

[40] Medeiros-Silva J., Mance D., Daniëls M., Jekhmane S., Houben K., Baldus M., Weingarth M. (2016). ^1^H-detected solid-state
NMR studies of water-inaccessible proteins in vitro and in situ. Angew. Chem., Int. Ed..

[41] Tugarinov V., Muhandiram R., Ayed A., Kay L. E. (2002). Four-Dimensional
NMR Spectroscopy of a 723-Residue Protein: Chemical Shift Assignments
and Secondary Structure of Malate Synthase G. J. Am. Chem. Soc..

[42] Schmidt E., Güntert P. (2012). A new algorithm
for reliable and general NMR resonance
assignment. J. Am. Chem. Soc..

[43] Barbet-Massin E., Pell A. J., Retel J. S., Andreas L. B., Jaudzems K., Franks W. T., Nieuwkoop A. J., Hiller M., Higman V., Guerry P. (2014). Rapid proton-detected NMR assignment for proteins with
fast magic angle spinning. J. Am. Chem. Soc..

[44] Ravera E., Cerofolini L., Martelli T., Louka A., Fragai M., Luchinat C. (2016). 1H-detected solid-state NMR of proteins entrapped in
bioinspired silica: a new tool for biomaterials characterization. Sci. Rep..

[45] Matlahov I., van der Wel P. C. A. (2018). Hidden
motions and motion-induced invisibility: Dynamics-based
spectral editing in solid-state NMR. Methods.

[46] Shen Y., Bax A. (2013). Protein backbone and
sidechain torsion angles predicted from NMR
chemical shifts using artificial neural networks. J. Biomol. NMR.

[47] Knödlstorfer S., Toscano G., Ptaszek A. L., Kontaxis G., Napoli F., Schneider J., Maier K., Kapitonova A., Lichtenecker R. J., Schanda P., Konrat R. (2025). A Novel HMBC-CC-HMQC
NMR Strategy for Methyl Assignment Using Triple-^13^C-Labeled
α-Ketoisovalerate Integrated with UCBShift 2.0. J. Mol. Biol..

[48] Kerfah R., Hamelin O., Boisbouvier J., Marion D. (2015). CH_3_-specific
NMR assignment of alanine, isoleucine, leucine and valine methyl groups
in high molecular weight proteins using a single sample. J. Biomol. NMR.

[49] Guo C., Tugarinov V. (2009). Identification
of HN-methyl NOEs in large proteins
using simultaneous amide-methyl TROSY-based detection. J. Biomol. NMR.

[50] Lewandowski J. R., Sass H. J., Grzesiek S., Blackledge M., Emsley L. (2011). Site-specific measurement of slow
motions in proteins. J. Am. Chem. Soc..

[51] Schanda P., Ernst M. (2016). Studying dynamics by magic-angle spinning solid-state NMR spectroscopy:
Principles and applications to biomolecules. Prog. Nucl. Magn. Reson. Spectrosc..

[52] Krushelnitsky A., Reichert D., Saalwächter K. (2013). Solid-state
NMR approaches to internal
dynamics of proteins: from picoseconds to microseconds and seconds. Acc. Chem. Res..

[53] Bonaccorsi M., Le Marchand T., Pintacuda G. (2021). Protein structural dynamics by Magic-Angle
Spinning NMR. Curr. Opin. Struct. Biol..

[54] Rovó P., Linser R. (2018). Microsecond Timescale
Protein Dynamics: a Combined
Solid-State NMR Approach. ChemPhysChem.

[55] Shimozawa Y., Himiyama T., Nakamura T., Nishiya Y. (2021). Structural
analysis
and reaction mechanism of malate dehydrogenase from Geobacillus stearothermophilus. J. Biochem..

[56] Taguchi H. (2017). The simple
and unique allosteric machinery of Thermus caldophilus lactate dehydrogenase:
Structure-function relationship in bacterial allosteric LDHs. Adv. Exp. Med. Biol..

[57] Arai K., Ishimitsu T., Fushinobu S., Uchikoba H., Matsuzawa H., Taguchi H. (2010). Active and inactive state structures of unliganded
Lactobacillus casei allosteric L-lactate dehydrogenase. Proteins.

[58] Tollinger M., Sivertsen A. C., Meier B. H., Ernst M., Schanda P. (2012). Site-resolved
measurement of microsecond-to-millisecond conformational-exchange
processes in proteins by solid-state NMR spectroscopy. J. Am. Chem. Soc..

[59] Sun H., Kay L. E., Tugarinov V. (2011). An optimized
relaxation-based coherence
transfer NMR experiment for the measurement of side-chain order in
methyl-protonated, highly deuterated proteins. J. Phys. Chem. B.

[60] Mittermaier A., Kay L. E., Forman-Kay J. D. (1999). Analysis
of deuterium relaxation-derived
methyl axis order parameters and correlation with local structure. J. Biomol. NMR.

[61] Igumenova T. I., Frederick K. K., Wand A. J. (2006). Characterization of the fast dynamics
of protein amino acid side chains using NMR relaxation in solution. Chem. Rev..

[62] Hansen D. F., Kay L. E. (2011). Determining Valine
Side-Chain Rotamer Conformations
in Proteins from Methyl 13C Chemical Shifts: Application to the 360
kDa Half-Proteasome. J. Am. Chem. Soc..

[63] Hansen D. F., Neudecker P., Kay L. E. (2010). Determination of Isoleucine Side-Chain
Conformations in Ground and Excited States of Proteins from Chemical
Shifts. J. Am. Chem. Soc..

[64] Hansen D. F., Neudecker P., Vallurupalli P., Mulder F. A. A., Kay L. E. (2010). Determination
of Leu Side-Chain Conformations in Excited Protein States by NMR Relaxation
Dispersion. J. Am. Chem. Soc..

[65] Mulder F. A. A. (2009). Leucine
Side-Chain Conformation and Dynamics in Proteins from ^13^C NMR Chemical Shifts. ChemBioChem.

[66] Abad-Zapatero C., Griffith J. P., Sussman J. L., Rossmann M. G. (1987). Refined
crystal
structure of dogfish M4 apo-lactate dehydrogenase. J. Mol. Biol..

[67] Wilkinson J. H., Walter S. J. (1972). Oxamate as a differential
inhibitor of lactate dehydrogenase
isoenzymes. Enzyme.

[68] Waudby C. A., Ramos A., Cabrita L. D., Christodoulou J. (2016). Two-dimensional
NMR lineshape analysis. Sci. Rep..

[69] Iwata S., Kamata K., Yoshida S., Minowa T., Ohta T. (1994). T and R states
in the crystals of bacterial L-lactate dehydrogenase reveal the mechanism
for allosteric control. Nat. Struct. Biol..

[70] Iorio A., Brochier-Armanet C., Mas C., Sterpone F., Madern D. (2022). Protein conformational
space at the edge of allostery: Turning a nonallosteric malate dehydrogenase
into an “allosterized” enzyme using evolution-guided
punctual mutations. Mol. Biol. Evol..

[71] Ikehara Y., Arai K., Furukawa N., Ohno T., Miyake T., Fushinobu S., Nakajima M., Miyanaga A., Taguchi H. (2014). The core of
allosteric motion in Thermus caldophilus L-lactate dehydrogenase. J. Biol. Chem..

[72] Zhang D., Itin B., McDermott A. E. (2019). TmDOTP: An NMR-based thermometer
for magic angle spinning NMR experiments. J.
Magn. Reson..

[73] Lee D., Hilty C., Wider G., Wüthrich K. (2006). Effective
rotational correlation times of proteins from NMR relaxation interference. J. Magn. Reson..

[74] Mitternacht S. (2016). FreeSASA:
An open source C library for solvent accessible surface area calculations. F1000Research.

[75] Schanda P., Napoli F. (2026). Data and scripts for:
Integrated solid/solution NMR
assignment allows mapping dynamics and ligand binding in a 134 kDa
enzyme. Institute of Science and Technology
Austria.

