# Supplementary material for: Integrated Solid/Solution NMR Assignment Enables Mapping of Dynamics and Ligand Binding in a 134 kDa Enzyme
Source: J Am Chem Soc. 2026 Oct 13;148(42):45913–25. doi: 10.1021/jacs.6c14423 (PMC13721178; doi:10.1021/jacs.6c14423)
Supplement: Supplementary file 1 [file ja6c14423_si_001.pdf]

Supporting information for "Integrated solid/solution NMR assignment enables mapping of dynamics and ligand binding in a 134 kDa enzyme"

Federico Napoli, Rajkumar Singh, Anna Kapitonova, Virgil Aitenbichler, Giorgia Toscano, Barbara Perrone, and Paul Schanda

S1 Contributions of different MAS spectra to backbone assignment **S2**

S2 Determination of methyl order parameters and investigation of their link to structure **S2**
- S2.1 Relaxation-violated coherence transfer experiment to measure $S^2_{axis}\tau_c$ . . . S2
- S2.2 Determining the overall-tumbling correlation time τ_c . . . S3
- S2.3 Accounting for non-isotropic overall tumbling . . . S4
- S2.4 Potential artifacts of methyl order parameter measurements . . . S5
- S2.5 Exploring relationships of methyl-axis order parameters with structural parameters . . . S6

S3 Supplementary Tables **S8**
- S1 Assignment spectra statistics. . . . S8
- S2 Sample conditions and instrumental details for solution NMR spectra. . . . S9
- S3 Sample conditions and instrument details for MAS NMR spectra. . . . S9
- S4 Detailed NMR acquisition parameters. . . . S10
- S5 Correlation of S^2_{axis} with structural descriptors. . . . S12

S4 Supplementary Figures **S13**
- S1 ^{13}C detected 4D MAS NMR spectra of sedimented *Ii*MDH with a CPMAS CryoProbe. . . . S13
- S2 *Ii*MDH refolding does not alter its activity or oligomeric state. . . . S14
- S3 MAS NMR assignment of backbone and side-chains. . . . S15
- S4 Chemical shift-based prediction of secondary structures with TALOS-N. . . . S16
- S5 Automated assignment with FLYA and ablation studies. . . . S17
- S6 FLYA addition studies and minimal datasets. . . . S18
- S7 H^N-(TROSY)-N-H NOESY spectrum. . . . S19
- S8 HMBC-CC-HMQC spectrum. . . . S20
- S9 Solution TOCSY experiments. . . . S21
- S10 Assignment of different methyl group classes in solution-NMR through-bond experiments. . . . S22
- S11 TOCSY experiments for methyl assignment in the solid state. . . . S23
- S12 H-H-C-C NOESY spectrum reporting on through-space Ile, Leu and Val methyl contacts. . . . S24
- S13 NOESY spectra reporting on Ile, Met and Thr methyl contacts. . . . S25
- S14 H^C-C-H NOESY spectrum. . . . S26
- S15 Two-dimensional CH spectra of methyl groups. . . . S27
- S16 MAS NMR $R_{1\rho}$ relaxation decays reporting on µs-ms dynamics. . . . S28
- S17 ^{15}N CPMG experiment in solution. . . . S29
- S18 ^{15}N-TRACT and 3Q-relaxation buildups. . . . S30
- S19 Solvent Accessible Surface Area (SASA) and methyl order parameters. . . . S31
- S20 Chemical shift-based prediction of rotamer populations. . . . S32
- S21 Titration analysis with TITAN. . . . S33
- S22 Ionic strength control for the oxamate titration. . . . S34
- S23 Effect of δ on fitted order parameters in the 3Q experiment. . . . S35

S5 Bibliography for supplementary information **S37**

S1 Contributions of different MAS spectra to backbone assignment

In the present work we combined 11 MAS spectra, for a total duration of 1385 hours of measurement, to achieve near-complete backbone assignment of *Ii*MDH. These included four 3D H-detected spectra (hCANH, hCONH, hcaCBcaNH, hNcocaNH), five 4D H-detected spectra (hcaCBCANH, hCACONH, hCOCANH, hcaCBcaCONH, hNCAcoNH), and two 4D C-detected spectra (CONCACX and CANCOCX). In the following, we will evaluate the individual and cumulative contribution of each experiment to the final assignment, with the aim of establishing a minimum experiment set needed for satisfactory backbone assignment. To do so, we performed several FLYA automated assignment calculations, utilizing different combinations of peaklists, and chemical shift predictions computed with UCBshift[1] (shiftassign_statistics parameter, .prot format).

First, we performed an ablation study, performing individual FLYA calculations where, one at a time, each peaklist was omitted. Tolerances of 0.1 ppm and 0.5 ppm were used for H and C/N frequencies, respectively. Given the large number of calculations, the number of runs was set to 10. Standard convergence of 80% was used. Moreover, we tested the effect of removing four different classes of experiments: (i) the experiments connecting consecutive N frequencies (hNcocaNH, hNCAcoNH); (ii) the three-dimensional H-detected experiments; (iii) the four-dimensional H-detected experiments; (iv) the C-detected experiments. We compared the converged chemical shift assignments (flya-strong.prot) with the assignment obtained with manual intervention using all the data presented in this manuscript. Such assignment corresponds to 100% in the following, and not the actual totality of assignable frequencies. An assignment was considered correct if it fell within the aforementioned tolerances, or (if larger) within the experimental error of our resonance assignment.

Results are presented in Figure S5. All the single-ablated runs performed similarly to the run where all the peaklists were included, indicating a high level of redundancy in the information carried by different peaklists. The run where all the 3D or 4D H-detected spectra were omitted performed only slightly worse, with the largest differences for the CB frequencies. Lastly, the run lacking the CONCACX and CANCOCX experiments also performed comparably, but of course resulted in a total lack of side-chain carbon assignments (excluding C_β), which are not encoded in any of the other inputs. This demonstrates that this approach can be extended to study other systems, even when C-detection instrumentation is not available. Also for methyl assignments, both the HMBC-CC-HMQC and TOCSY experiments presented in this work allow connecting the methyl moieties to C_α and C_β frequencies, without the need to extensively pre-assign side-chain carbons in MAS spectra.

Next, we performed an addition study, running FLYA with a single peaklist complemented by the hCANH and hCONH ones (which are short and sensitive experiments, usually measured in the initial exploratory phases), and UCBshift predictions (Figure S6a). We extracted all the runs' results and permuted them to investigate best complementarities, preferring alternatively backbone or side-chain assignments (Figure S6b-c). We combined these results and the ablation study ones to determine the minimal set required to achieve a satisfactory assignment of backbone and CBs frequencies (Figure S6d): this consists of CANCOCX, CONCACX, hNCAcoNH, hcaCBcaCONH and hcaCBCANH (5 spectra, 803 hours), or hCOCANH, hcaCBCANH, hNCAcoNH and hcaCBcaCONH (4 spectra, 657 hours) if C-detection instrumentation is not available.

S2 Determination of methyl order parameters and investigation of their link to structure

S2.1 Relaxation-violated coherence transfer experiment to measure $S^2_{axis}\tau_c$

The methyl-axis order parameters were determined from a triple-quantum (3Q) relaxation violated coherence transfer NMR experiment in solution, described elsewhere[2]. In brief, the experiment measures the time-dependence of the build-up of forbidden 3Q coherence (with intensity I_A) and the intensity evolution of a single-quantum coherence with intensity I_B as a function of the relaxation delay t. The ratio of these intensities depends, *inter alia*, on the sought cross-correlated relaxation with a rate η, and a term $\delta < 0$ that accounts for coupling between the rapidly and slowly decaying ^{1}H SQ coherences due to relaxation with

external protons, as follows:

$$I_A/I_B = \frac{3}{4}\frac{\eta\tanh\left(\sqrt{\eta^2+\delta^2}\,t\right)}{\sqrt{\eta^2+\delta^2}-\delta\tanh\left(\sqrt{\eta^2+\delta^2}\,t\right)} \tag{1}$$

$$\eta = \frac{9}{10}\left(\frac{\mu_0}{4\pi}\right)^2[P_2(\cos\theta_{axis,HH})]^2\frac{\gamma_H^4\hbar^2}{r_{HH}^6}S^2\tau_c \tag{2}$$

Two data sets, corresponding to I_A and I_B, were collected and the experimental ratio was fitted using a standard chi-square fit procedure, with η and δ as fit parameters.

$$\chi^2 = \sum_i\left(\frac{I_{A/B}^{\text{model}}(t_i) - I_{A/B}^{\text{data}}(t_i)}{\sigma_i}\right)^2 \tag{3}$$

where $I_{A/B}$ is a shorthand notation for the intensity ratio. The constants are as follows:

$$\begin{aligned}
&\gamma_H = 2.67522\times10^8\ \text{rad s}^{-1}\,\text{T}^{-1} && \gamma_C = 6.7283\times10^7\ \text{rad s}^{-1}\,\text{T}^{-1} && \gamma_N = -2.7126\times10^7\ \text{rad s}^{-1}\,\text{T}^{-1}\\
&\mu_0 = 4\pi\times10^{-7}\ \text{T}^2\,\text{m}^3\,\text{J}^{-1} && h = 6.626\times10^{-34}\ \text{J s} && \hbar = 1.054\times10^{-34}\ \text{J s}\\
&r_{HH} = 1.813\times10^{-10}\ \text{m} && \theta_{axis,HH} = \frac{\pi}{2} && P_2 = \frac{1}{2}(3\cos^2\theta - 1)
\end{aligned} \tag{4}$$

Methyl triple-quantum (3Q) relaxation experiments report on the product $S^2_{\text{axis}}\tau_c$, where S^2_{axis} is the squared generalized order parameter of the methyl symmetry (C–C) axis and τ_c is the correlation time for reorientation of that axis under overall molecular tumbling[2]. Extraction of S^2_{axis} therefore requires an independent estimate of τ_c. Because the rotational diffusion tensor of the protein is not isotropic, the appropriate τ_c is in principle different for every methyl group, depending on the orientation of its symmetry axis relative to the diffusion tensor frame. We therefore analyzed the 3Q data in two ways: (1) using a single, molecule-wide τ_c, and (2) using site-specific τ_c values derived from the hydrodynamically predicted diffusion tensor.

Prior to that, we describe how the tumbling correlation time was determined experimentally.

S2.2 Determining the overall-tumbling correlation time τ_c

A cross-correlated relaxation experiment, known as TRACT[3], and implemented in NMRlib[4], was used to record the decay of ^{15}N coherence. Two experiments were performed, corresponding to the decays of ^{15}N coherence with the amide hydrogen in either α or β states; these rate constants are denoted as $R_{2,\alpha}$ and $R_{2,\beta}$, and were obtained by fitting a mono-exponential function to the integrals of the amide signals (see Figure S18). The apparent τ_c is then obtained from:

$$C_{\text{CCR}} = S^2\frac{4}{15}\frac{\mu_0}{4\pi}\hbar\gamma_N^2\gamma_H B_0\Delta\sigma\, r_{NH}^{-3}P_2(\cos\theta_{NH,CSA}) \tag{5}$$

$$\tau_c = \frac{|R_{2,\beta} - R_{2,\alpha}|}{2\,|C_{\text{CCR}}|} \tag{6}$$

Hereby, the constants are:

$$r_{NH} = 1.021\times10^{-10}\ \text{m} \qquad B_0 = 18.8\ \text{T} \qquad \Delta\sigma = -140\times10^{-6} \qquad \theta_{NH,CSA} = 0^\circ \tag{7}$$

The experiment was done as two series of 1D spectra and from the difference in the apparent relaxation rate constants the correlation time, τ_c, was calculated to provides a single number, $\tau_c^{\text{TRACT}} = 39.0 \pm 3.9$ ns, which corresponds to the average over all amide sites that are detected.

It should be noted at this point that the parameters used in the calculation of τ_c from the two relaxation rate constants produce values of τ_c that match to very excellent precision (within 0.4%) those calculated

with the parameters used in the original TRACT publication by Lee *et al.*[3], which used $\Delta\sigma = -160 \times 10^{-6}$, $\theta_{NH,CSA}$=17° and r_{NH}=1.02 Å. The reason for this very small difference in the calculated τ_c despite different parameters is that the differences in angle and $\Delta\sigma$ in the two sets of parameters compensate. Later, Yao *et al.* have quantified CSA parameters to high precision and proposed values of $\Delta\sigma = -166 \times 10^{-6}$, $\theta_{NH,CSA}$=19.6° and r_{NH}=1.04 Å, whereby the bond length and CSA takes into account the zero-point libration[5]. Using that set of values would result in a slightly (ca. 7%) higher τ_c, and accordingly lower methyl-axis order parameters. Lastly, it shall be noted that the assumption that the protein behaves as a rigid body, underlying the TRACT approach, tends to result in slightly underestimated values of τ_c, as shown earlier[6]; this might explain the slightly lower value of τ_c found by TRACT compared to HydroNMR (see below). For the present study which primarily seeks site-to-site variations this multiplicative factor is not relevant.

If we assume that the molecular tumbling can be described as isotropic tumbling, converting the values of $S^2_{\text{axis}}\tau_c$ to site-specific S^2_{axis} values is straightforward:

$$S^2_{\text{axis},i}(\text{iso}) = \frac{\left(S^2_{\text{axis}}\tau_c\right)_i}{\tau_c^{\text{TRACT}}},$$

for every methyl site i. This treatment implicitly assumes that all methyl axes experience the same overall tumbling, an assumption that is considered more closely in the following section.

S2.3 Accounting for non-isotropic overall tumbling

More realistically, molecular tumbling is anisotropic; consequently, a site-specific τ_c of effective overall tumbling should be used for each methyl site. In the following we describe this procedure; in brief, the result is that the difference, compared to the above isotropic-tumbling case, is modest for *Ii*MDH.

To assess the tumbling anisotropy, we used the program HydroNMR[7] that models the diffusion properties from the structure, using the crystal structure (6QSS) as input, an atomic element radius of 3.1 Å, a radius of beads in the shell from 2.5 to 3.5 Å, a temperature of 323.0 K and a solvent viscosity of 0.00650 P.

Diagonalisation of the rotational block of the 6 × 6 diffusion matrix by HydroNMR gave principal values of the diffusion tensor of $D_x = 3.428 \times 10^6$, $D_y = 3.098 \times 10^6$ and $D_z = 3.786 \times 10^6$ s^{-1} ($D_{\max}/D_{\min} = 1.22$; $D_{\text{iso}} = 3.437 \times 10^6$ s^{-1}, corresponding to $1/6D_{\text{iso}} = 48.5$ ns). Note that in this analysis we are primarily interested in the anisotropy of the tensor, rather than in the absolute values. The anisotropy ratio is ca. 1.2 (the fastest axis tumbles ca. 20% faster than the slowest). This modest anisotropy implies that site-to-site variations of effective τ_c are, at most, 20%, namely for extreme cases of methyl axes that are aligned parallel to the two diffusion axes with the most extreme values. We analyze further below how much such an anisotropic tumbling alters the obtained methyl order parameters.

For a rigid vector tumbling in a fully asymmetric diffusion tensor, the second-rank orientational correlation function is a sum of five exponentials[8]:

$$C(t) = \sum_{k=1}^{5} A_k\, e^{-t/\tau_k},$$

with time constants determined solely by the tensor,

$$\tau^{-1}_{1,2,3} = 4D_{x,y,z} + \textstyle\sum_{j\neq x,y,z} D_j, \qquad \tau^{-1}_{4,5} = 6\bar{D} \mp 6\sqrt{\bar{D}^2 - L^2},$$

where $\bar{D} = \frac{1}{3}(D_x + D_y + D_z)$ and $L^2 = \frac{1}{3}(D_xD_y + D_yD_z + D_xD_z)$, and with amplitudes A_k that depend only on the direction cosines (x, y, z) of the vector in the principal-axis frame ($A_1 = 3y^2z^2$, $A_2 = 3x^2z^2$, $A_3 = 3x^2y^2$, and $A_{4,5}$ given by the standard expressions[9]). For this tensor the five time constants were 51.46, 51.01, 48.55, 46.15 and 45.84 ns.

The effective, orientation-dependent correlation time of each vector was taken as the time integral of its correlation function,

$$\tau_{c,i} = \int_0^\infty C_i(t)\,\mathrm{d}t = \sum_{k=1}^{5} A_{k,i}\,\tau_k, \qquad J_i(0) = \tfrac{2}{5}\,\tau_{c,i},$$

which is the value that reproduces the correct zero-frequency spectral density. The implementation was validated by reproducing the per-residue amide values reported by HydroNMR for 941 N–H vectors of the same structure (Pearson $r = 1.00000$, RMSD 0.005 ns), and independently by numerical diagonalisation of the rank-2 rotational diffusion operator $\hat{H} = D_x \hat{J}_x^2 + D_y \hat{J}_y^2 + D_z \hat{J}_z^2$, which gave identical results.

$\tau_{c,i}$ was evaluated for the Ile Cγ1–Cδ1, Leu Cγ–Cδ1/Cδ2 and Val Cβ–Cγ1/Cγ2 bond vectors in all four chains of the tetramer. Because the four subunits are related by 222 symmetry and are magnetically equivalent, the four copies of each site were averaged. Over the 148 methyl sites the mean was $\langle \tau_c^{\mathrm{calc}} \rangle = 48.4$ ns, with a site-to-site range of 46.3–50.6 ns.

The hydrodynamically predicted mean exceeds the TRACT value, most plausibly because of uncertainty in the effective solvent viscosity and hydration layer. Since only the relative orientation dependence of the calculated tensor was considered reliable, the site-specific values were rescaled to match the experimental mean,

$$\tau_{c,i}^{\mathrm{scaled}} = \tau_{c,i}^{\mathrm{calc}} \cdot \frac{\tau_c^{\mathrm{TRACT}}}{\langle \tau_c^{\mathrm{calc}} \rangle},$$

and order parameters were obtained as

$$S^2_{\mathrm{axis},i}(\mathrm{aniso}) = \frac{\left(S^2_{\mathrm{axis}} \tau_c\right)_i}{\tau_{c,i}^{\mathrm{scaled}}}.$$

Substituting the two preceding equations shows that the experimental correlation time cancels, so that the anisotropic treatment amounts to a purely geometric rescaling of the isotropic result:

$$S^2_{\mathrm{axis},i}(\mathrm{aniso}) = S^2_{\mathrm{axis},i}(\mathrm{iso}) \cdot \frac{\langle \tau_c^{\mathrm{calc}} \rangle}{\tau_{c,i}^{\mathrm{calc}}}.$$

The corrected order parameters are therefore independent of the absolute value of τ_c^{TRACT} and depend only on the anisotropy of the predicted diffusion tensor. For the present system the correction factors ranged from 0.958 to 1.045 (mean 1.002), corresponding to a mean change in S^2_{axis} of 0.007 and a maximum change of 0.020; the mean experimental uncertainty in S^2_{axis} was 0.023. For 25 out of 90 sites the correction due to the anisotropic tumbling exceeds the error bar slightly; for all other sites, the correction is smaller than the experimental uncertainty. The consideration of anisotropic tumbling does not alter the conclusions drawn from this experiment.

It is worth noting the limitations of this treatment. As usual for any Modelfree-type treatment, the calculation treats each methyl symmetry axis as rigidly attached to the tumbling body, so the resulting $\tau_{c,i}$ represent the overall-tumbling contribution only; all internal motion is contained in S^2_{axis}. Fast three-fold methyl rotation is separable and contributes the usual factor $P_2(\cos 109.5°)^2 = 1/9$, which is not included in the values reported here. Val and Leu methyls were assigned to the Cγ2 and Cδ2 positions of the deposited structure, consistent with the stereospecific (pro-S) labeling scheme employed; the two methyls of a given Val or Leu differ in τ_c by 1.5 ns on average (maximum 3.9 ns), so an incorrect stereospecific assignment would introduce an error comparable to the anisotropy correction itself. Ile δ1 sites are not subject to this ambiguity. Finally, seven sites showed a spread greater than 1 ns among the four crystallographically independent copies, reflecting inconsistent side-chain rotamer modeling rather than genuine inequivalence; their predicted τ_c values carry a correspondingly larger uncertainty. Of note, the precision with which the methyl-axis orientation has been reported in the crystal structure will also influence this analysis; model errors in the axis orientation would result in a different effective correlation time.

The Python fitting routines for both experiments are provided with the data repository; see main text for the access details.

S2.4 Potential artifacts of methyl order parameter measurements

The fit of the 3Q buildup curves involves two parameters: η, which is the sought quantity proportional to S^2_{axis}, and δ (see Eq. 1). According to Sun *et al.*[2], the latter parameter comprises effects from proton spins external to the methyl group. As a first note, from the functional form of Eq. 1, δ determines the plateau

of the buildup curve, but not the buildup itself. Hence, as we will show below, changes in δ impact the precision with which S^2_{axis} can be determined, but not its accuracy.

We then interrogated how δ impacts the fitted values of S^2_{axis}, through synthetic data inspired by the experimental ones (Figure S23). In brief, we have assumed three order parameters, corresponding to the minimum, mean and maximum found in our data set. Using the experimentally determined tumbling correlation time, we have generated synthetic 3Q buildup curves, and fitted Eq. 1 to those. We found that by artificially altering δ to generate the curve and then fitting Eq. 1 to obtain η (or, equivalently, S^2_{axis}) the bias introduced by the value of δ is generally less than 1% (Figure S23a). However, with large negative δ the "amplitude" of the buildup decreases, and therefore the precision decreases. This analysis also shows that it is important to fit δ: setting it to zero leads to poor fits and potentially a strong bias in η. All these scenarios are shown in Figure S23.

We have also considered whether conformational exchange (millisecond dynamics) may impact the 3Q experiment using methyl MQ CPMG (Napoli *et al.*, manuscript in preparation). We have calculated the "amplitude" of CPMG dispersion curves, $\Delta R_{2,eff}$, by first fitting an exchange model to the experimental CPMG profile, and then calculating $\Delta R_{2,eff}$ as the difference between $R_{2,eff}$ at the lowest and highest CPMG frequencies, i.e. the first and last point of the fitted CPMG curve. We did not find any correlation between S^2_{axis} and $\Delta R_{2,eff}$, suggesting that millisecond time scale exchange processes do not impact the fit of the 3Q experiment (data not shown).

S2.5 Exploring relationships of methyl-axis order parameters with structural parameters

Methyl-axis order parameters S^2_{axis} were available for 90 methyl groups (Ile δ_1, Val γ_2, Leu δ_2), which constitute the complete set of Ile/Val/Leu residues resolved in the crystal structure 6QSS. The asymmetric unit contains four chains (A: residues 2–308; B, C, D: 3–309); all descriptors below were computed independently in each chain and averaged, so that each entry rests on four values, except Leu309, which is absent from chain A and is averaged over three. The experimental values span S^2_{axis} = 0.089–0.827 with a mean of 0.382 and a standard deviation of 0.144.

Because the deposited coordinates contain no hydrogens, the structure was protonated at all sites using `pdb2pqr` with AMBER templates and hydrogen-bond network optimization, yielding 18864 atoms. The procedure leaves the heavy-atom frame essentially untouched (mean displacement 0.007 Å); the only atoms moving by more than 0.3 Å are 30 atoms belonging to Asn/Gln/His side chains whose terminal amide or imidazole groups were flipped. No methyl carbon of interest was displaced. All analyses were performed on the complete tetramer, since the rotational correlation time of τ_c = 39.0 ns indicates that the oligomer, not the protomer, is the relevant species in solution.

S2.5.1 Structural descriptors

Solvent accessibility. Solvent-accessible surface areas were computed with the FreeSASA Python module[10] at its default settings (Lee–Richards algorithm, 20 slices per atom, ProtOr radii, probe radius 1.4 Å).

Local packing density. Packing was quantified by the contact density[11]: the number of non-hydrogen atoms within a sphere of radius R_C = 7.1 Å centered on the methyl carbon, corresponding to a local volume of ~1.5 nm^3. Atoms of the methyl-bearing residue itself were excluded, and the calculation was performed on the complete tetramer and averaged over the four chains. This descriptor was introduced to relate crystallographic mean-square displacements to local packing and is therefore a natural structural counterpart to the order parameters analyzed here. Contact densities range from 21 to 78 atoms, with a mean of 61. Varying R_C between 6 and 8 Å changes the correlations reported below by less than 0.06.

The Ming–Brüschweiler contact model. We further attempted a static-structure-based approach to model the methyl order parameters, the contact model of Ming and Brüschweiler[12], an extension of the backbone local-contact model of Zhang and Brüschweiler[13]. It predicts methyl order parameters from a single static structure — no molecular dynamics trajectory is required — from the local contacts of the methyl carbon together with the number of mobile dihedral angles separating the methyl from the backbone.

S2.5.2 Correlation of structural descriptors with methyl-axis order parameters

All descriptors correlate with S^2_{axis} in the direction anticipated on physical grounds — denser packing and lower solvent accessibility accompany higher order — and all but the burial depth reach nominal significance (Table S5). The result is robust with respect to the details of the calculation: varying the contact radius R_C between 6 and 10 Å changes the correlation coefficient by less than 0.08, and restricting the analysis to the 80 methyls with $\sigma(S^2) < 0.05$ strengthens the correlations slightly (contact density $r = +0.361$; relative side-chain SASA $r = -0.329$; methyl SASA $r = -0.368$).

The magnitude of the effect is nevertheless modest, as exemplified by the correlation with SASA, shown in Figure S19. A multiple regression on contact density, relative side-chain SASA and burial depth yields $R = 0.33$, i.e. $R^2 = 0.11$: structure in this static sense accounts for roughly one tenth of the variance in the order parameters. Grouping the methyls by burial class gives $\langle S^2 \rangle = 0.40 \pm 0.02$ (buried, $n = 57$), 0.41 ± 0.03 (partially exposed, $n = 20$) and 0.27 ± 0.04 (exposed, $n = 13$). Buried and partially exposed methyls are thus indistinguishable from one another; only the exposed group stands apart, and it does so by about 0.14 in S^2, whereas the scatter within any single group exceeds 0.35.

Binning by contact density tells the same story. The least densely packed quintile has $\langle S^2 \rangle = 0.28 \pm 0.03$, while the remaining four quintiles lie between 0.34 and 0.43 with no systematic trend among them. The relationship is therefore a threshold rather than a gradient: sparse packing enforces disorder, but above a modest density the packing environment no longer predicts the order parameter.

Two further observations sharpen this. First, within the buried subset alone the correlation with contact density vanishes ($n = 57$, $r = +0.13$, $p = 0.34$), whereas among the partially exposed and exposed methyls it is appreciable ($n = 33$, $r = +0.43$, $p = 0.01$). Second, the methyl-group SASA saturates: 54 of the 90 methyls have an accessible area below 0.5 Å^2, so this descriptor cannot discriminate among them at all, and restricting the analysis to the 36 methyls with measurable accessibility raises the correlation to $r = -0.491$ ($\rho = -0.481$, $p = 0.003$). The entire structural signal therefore originates from the solvent-exposed minority; dense packing appears necessary but not sufficient for high order, and whether a well-packed methyl is genuinely rigid or hops between rotamers within a cavity that accommodates both is not decided by the packing density of a single conformer.

It is worth noting that Halle's local density model predicts crystallographic mean-square displacements to be inversely proportional to the contact density[11]. The present data are qualitatively consistent with that picture but fall far short of such a tight relationship, which is expected given that S^2_{axis} reports on picosecond-to-nanosecond reorientation of a single bond vector rather than on the time- and ensemble-averaged displacement of an atom.

S3 Supplementary Tables

Table S1: Assignment spectra statistics.

Backbone assignment experiment (MAS)	Number of peaks
hCANH (3D)	250
hCONH (3D)	222
hcaCBcaNH (3D)	193
hcaCBCANH (4D)	223
hCACONH (4D)	177
hCOCANH (4D)	243
hcaCBcaCONH (4D)	430
hNcocaNH (3D)	220
hNCAcoNH (4D)	228
CONCACX (4D, C-det.)	864
CANCOCX (4D, C-det)	799

Side-chain assignment experiment	Number of peaks	Assigned methyls
LV HMBC-CC-HMQC	180*	83 (71.6%)
ILV TOCSYs (L2/L3/L4)	90*/93*/116*	72 (48.6%)
MAS ILV hCCH TOCSY	98*	44 (29.7%)
MAS ILV hCccNH TOCSY	22	
ILV H-H-C-C NOESY	105*	70 (47.3%)
MIT H-H-C/H-C-C NOESY	86*/104*	55 (96.5%)
ILV H^N-(TROSY)-N-H NOESY/H^C-C-H NOESY	315*/183*	70 (47.3 %)

* : Diagonal peaks were omitted from the count

Table S2: Sample conditions and instrumental details for solution NMR spectra.

Experiment	Sample labeling	Concentration	Solvent	pH	T (K)	Field (MHz)	Exp. time (h)
HMBC-CC-HMQC	U-^{2}H,^{13}C,^{15}N, α-ketoisovalerate, V168A	400 µM	D_2O	7	318.5	800	63
TOCSY L2	U-^{2}H,^{15}N, ketobutyrate, acetolactate	400 µM	D_2O	7	318.5	800	93
TOCSY L3	U-^{2}H,^{15}N, ketobutyrate, acetolactate	400 µM	D_2O	7	318.5	800	68
TOCSY L4	U-^{2}H,^{15}N, ketobutyrate, acetolactate	400 µM	D_2O	7	318.5	800	108
H^N-N-H NOESY	U-^{2}H,^{15}N, ketobutyrate, acetolactate	400 µM	H_2O	7	318.5	800	69
H^C-C-H NOESY	U-^{2}H,^{15}N, ketobutyrate, acetolactate	400 µM	H_2O	7	318.5	800	73
H-H-C-C NOESY	U-^{2}H,^{15}N, Ile-δ_1, Leu-δ_2, Val-γ_2	400 µM	D_2O	7	318.5	800	212
^{15}N TRACT	U-^{2}H,^{15}N, Ile-δ_1, Leu-δ_2, Val-γ_2	400 µM	H_2O	7	318.5	800	3
Methyl 3Q-relaxation	U-^{2}H,^{15}N, Ile-δ_1, Leu-δ_2, Val-γ_2	400 µM	H_2O	7	318.5	800	110
H-H-C NOESY	U-^{2}H,^{15}N, Ile-δ_1, Met-ε, Thr-γ	400 µM	D_2O	7	318.5	800	55
H-C-C NOESY	U-^{2}H,^{15}N, Ile-δ_1, Met-ε, Thr-γ	400 µM	D_2O	7	318.5	800	77
^{1}H-^{15}N-TROSY	U-^{2}H,^{13}C,^{15}N (refolded)	496.3 µM	H_2O	6	318.5	800	3
^{1}H-^{15}N-TROSY	U-^{2}H,^{13}C,^{15}N (refolded)	259.1 µM, OXM: 2.5 mM	H_2O	6	318.5	800	3
^{1}H-^{15}N-TROSY	U-^{2}H,^{13}C,^{15}N (refolded)	389.5 µM, OXM: 4.7 mM	H_2O	6	318.5	800	3
^{1}H-^{15}N-TROSY	U-^{2}H,^{13}C,^{15}N (refolded)	586.3 µM, OXM: 24.4 mM	H_2O	6	318.5	800	3
^{1}H-^{15}N-TROSY	U-^{2}H,^{13}C,^{15}N (refolded)	447.6 µM, OXM: 36.7 mM	H_2O	6	318.5	800	3
^{1}H-^{15}N-TROSY	U-^{2}H,^{13}C,^{15}N (refolded)	442.0 µM, OXM: 49.0 mM	H_2O	6	318.5	800	3
^{1}H-^{15}N-TROSY	U-^{2}H,^{13}C,^{15}N (refolded)	395.7 µM, OXM: 61.2 mM	H_2O	6	318.5	800	3
^{1}H-^{15}N-TROSY CPMG	U-^{2}H,^{13}C,^{15}N (refolded)	500 µM	H_2O	7	318.5	800	34

Table S3: Sample conditions and instrument details for MAS NMR spectra.

Experiment	Sample labeling	Rotor (mm)	MAS (kHz)	pH	Set T (K)	Real T (K)	Field (MHz)	NUS (%)	Exp. time (h)
hCANH	U-^{2}H/^{13}C/^{15}N (refolded)	1.9	38	7	255	305	700	/	94
hCONH	U-^{2}H/^{13}C/^{15}N (refolded)	1.9	38	7	255	305	700	/	50
hcaCBcaNH	U-^{2}H/^{13}C/^{15}N	1.9	38	7	255	n/a	600	/	72
hcaCBCANH	U-^{2}H/^{13}C/^{15}N (refolded)	1.9	38	7	255	305	700	23.5	165
hCACONH	U-^{2}H/^{13}C/^{15}N (refolded)	1.9	38	7	255	305	700	38.8	163
hCOCANH	U-^{2}H/^{13}C/^{15}N (refolded)	1.9	38	7	255	305	700	18.1	104
hcaCBcaCONH	U-^{2}H/^{13}C/^{15}N (refolded)	1.9	38	7	255	305	700	24.5	195
hNcocaNH	U-^{2}H/^{13}C/^{15}N (refolded)	1.9	38	7	255	305	700	/	99
hNCAcoNH	U-^{2}H/^{13}C/^{15}N (refolded)	1.9	38	7	255	305	700	25	193
$R_{1\rho}$ relaxation	U-^{2}H/^{13}C/^{15}N (refolded)	1.9	38	7	255	305	700	60	95
hCccNH TOCSY	U-^{2}H/^{15}N, ketobutyrate/acetolactate	1.3	55.555	7	260	310	700	75	124
hCCH TOCSY	U-^{2}H/^{15}N, ketobutyrate/acetolactate	1.3	55.555	7	260	310	700	65	163
CONCACX	U-^{13}C/^{15}N	3.2	15	7	283	n/a	600	26	138
CANCOCX	U-^{13}C/^{15}N	3.2	15	7	283	n/a	600	19.2	112

Table S4: Detailed NMR acquisition parameters. A spreadsheet of this table and all raw data in Bruker format are provided in the data repository. See the "Data availability" section for access details.

**Experiment**	**SW (ppm)**	**Carrier (ppm)**	**Acquisition points/time (s)**	**NUS (%)**	**Transfer/mixing times (us)**	**RF field strengths (kHz)**	**Decoupling****
3D hCANH, 38 kHz MAS	H: 29.74<br>N: 40<br>CA: 45	H: 5<br>N: 117<br>CA: 54	H: 1024/0.025<br>N: 140/0.025<br>CA: 140/0.0088	/	H-CA CP: 3900<br>CA-N CP: 10000<br>N-H CP: 1000	H-CA CP: 84.5 (H), 51 (CA)<br>CA-N CP: 8.5 (CA), 28.5 (N)<br>N-H CP: 52 (N), 85 (H)	1H dec (15N t): waltz16, p=35us, RF=8kHz<br>15N dec (1H t): waltz16, p=50us, RF=5kHz
3D hCONH, 38 kHz MAS	H: 29.74<br>N: 40<br>CO: 14	H: 6.2<br>N: 117<br>CO: 175	H: 1024/0.025<br>N: 96/0.017<br>CC: 98/0.0199	/	H-CO CP: 4000<br>CO-N CP: 10000<br>N-H CP: 1000	H-CO CP: 88.1 (H), 52 (CO)<br>CO-N CP: 8 (CO), 29 (N)<br>N-H CP: 52 (N), 85.5 (H)	1H dec (15N t): waltz16, p=25us, RF=10kHz<br>15N dec (1H t): waltz16, p=50us, RF=5kHz
3D hcaCBcaNH, 38 kHz MAS	H: 29.74<br>N: 36<br>CA/CB: 75	H: 7.5<br>N: 118<br>CA/CB: 38	H: 532/0.015<br>N: 80/0.018<br>CA: 120/0.0053	/	H-CA CP: 3600<br>CA-N CP: 8000<br>N-H CP: 1200<br>CA-CB INEPT: 6000	H-CA CP: 62.6 (H), 23 (CA)<br>CA-N CP: 8 (CA), 28 (N)<br>N-H CP: 24.4 (N), 63.3 (H)	1H dec (15N t): waltz16, p=25us, RF=10kHz<br>15N dec (1H t): waltz16, p=50us, RF=5kHz
4D hcaCBCANH, 38 kHz MAS	H: 29.75<br>CA: 30<br>N: 36<br>CB: 70	H: 6.2<br>CA: 54<br>N: 119.5<br>CB: 39	H: 512/0.013<br>CA: 58/0.0055<br>N: 50/0.0098<br>CB: 98/0.0040	23.5	H-CA CP: 3250<br>CA-N CP: 10000<br>N-H CP: 1000<br>CA-CB INEPT: 6300	H-CA CP: 87.8 (H), 51 (CA)<br>CA-N CP: 6.3 (CA), 29.5 (N)<br>N-H CP: 52 (N), 85.5 (H)	1H dec (15N t): waltz16, p=25us, RF=10kHz<br>15N dec (1H t): waltz16, p=50us, RF=5kHz
4D hCACONH, 38 kHz MAS	H: 29.75<br>CO: 13<br>N: 36<br>CA: 40	H: 5.1<br>CO: 175<br>N: 118<br>CA: 54	H: 610/0.015<br>CO: 48/0.0105<br>N: 72/0.0141<br>CA: 64/0.0045	38.8	H-CA CP: 2485<br>CA-CO BSH-CP: 2500<br>CO-N CP: 9368<br>N-H CP: 1000	H-CA CP: 93.7 (H), 54 (CO)<br>CA-CO BSH-CP: 14.6<br>CO-N CP: 12 (CO), 26.5 (N)<br>N-H CP: 52 (N), 93.6 (H)	1H dec (15N t): waltz16, p=31us, RF=6kHz<br>15N dec (1H t): waltz16, p=50us, RF=5kHz
4D hCOCANH, 38 kHz MAS	H: 29.75<br>CA: 40<br>N: 36<br>CO: 14	H: 6.2<br>CA: 54<br>N: 117<br>CO: 175	H: 620/0.015<br>CA: 64/0.0045<br>N: 64/0.013<br>CO: 56/0.0114	18.1	H-CO CP: 4000<br>CO-CA BSH-CP: 4000<br>CA-N CP: 10000<br>N-H CP: 1000	H-CO CP: 87 (H), 51 (CO)<br>CO-CA BSH-CP: 12<br>CA-N CP: 9.5 (CA), 26.3 (N)<br>N-H CP: 52 (N), 85 (H)	1H dec (15N t): waltz16, p=25us, RF=10kHz<br>15N dec (1H t): waltz16, p=50us, RF=5kHz
4D hcaCBcaCONH, 38 kHz MAS	H: 29.75<br>CO: 15<br>N: 34<br>CA/CB: 70	H: 6.2<br>CO: 175<br>N: 119.5<br>CA/CB: 39	H: 1024/0.025<br>CO: 40/0.0076<br>N: 52/0.011<br>CA/CB: 76/0.0031	24.5	H-CA CP: 4000<br>CA-CO BSH-CP: 4000<br>CO-N CP: 10000<br>N-H CP: 1000<br>CA-CB INEPT: 3500	H-CA CP: 88.4 (H), 51 (CA)<br>CA-CO BSH-CP: 12.1<br>CO-N CP: 8 (CO), 28.5 (N)<br>N-H CP: 52 (N), 86 (H)	1H dec (15N t): waltz16, p=25us, RF=10kHz<br>15N dec (1H t): waltz16, p=50us, RF=5kHz
3D hNcocaNH, 38 kHz MAS	H: 29.75<br>N1: 36<br>N2: 36	H: 6.2<br>N1: 119<br>N2: 119	H: 512/0.013<br>N1: 96/0.019<br>N2: 96/0.019	/	H-N CP: 1000<br>N-CO CP: 10000<br>CO-CA BSH-CP: 4200<br>CA-N CP: 10000	H-N CP: 85 (H), 52 (N)<br>N-CO CP: 27.9 (N), 9.5 (CO)<br>CO-CA BSH-CP: 12.2<br>CA-N CP: 9.5 (CA), 27.9 (N)	1H dec (15N t): waltz16, p=25us, RF=10kHz<br>15N dec (1H t): waltz16, p=50us, RF=5kHz
4D hNCAcoNH, 38 kHz MAS	H: 29.75<br>CA: 40<br>N1: 36<br>N2: 36	H: 6.2<br>CA: 54<br>N1: 117<br>N2: 117	H: 620/0.015<br>CA: 60/0.0043<br>N1: 72/0.014<br>N2: 72/0.014	25	H-N CP: 1000<br>N-CA CP: 10000<br>CA-CO BSH-CP: 10000<br>CO-N CP: 10000	H-N CP: 85.5 (H), 52 (N)<br>N-CA CP: 9 (CA), 26.3 (N)<br>CA-CO BSH-CP: 12.9<br>CO-N CP: 9 (CO), 27.4 (N)	1H dec (15N t): waltz16, p=25us, RF=10kHz<br>15N dec (1H t): waltz16, p=50us, RF=5kHz
hCANH R1ρ, 38 kHz MAS	H: 29.75<br>N: 34<br>CA: 40	H: 6.2<br>N:118<br>CA: 54	H: 624/0.015<br>N: 96/0.020<br>CA: 72/0.0051	60	H-CA CP: 4000<br>CA-N CP: 10000<br>N-H CP: 1200<br>Relax: [5, 20, 40, 60, 80, 100, 130] ms	H-CA CP: 86.2 (H), 51 (CA)<br>CA-N CP: 10.6 (CA), 26.3 (N)<br>N-H CP: 52 (N), 85.2 (H)<br>SPINLOCK: 10	1H dec (15N t): waltz16, p=25us, RF=10kHz<br>15N dec (1H t): waltz16, p=16.7us, RF=15kHz
hCccNH TOCSY, 55.6 kHz MAS	H: 29.75<br>N: 36<br>C: 60	H: 5.5<br>N: 118<br>C: 40 17 (M) 54 (CA)	H: 624/0.015<br>N: 128/0.025<br>C: 296/0.014	75	H-C CP: 3000<br>CA-N CP: 9000<br>N-H CP: 1000<br>TOCSY (DIPSI): 21730	H-C CP: 92.3 (H), 40 (C)<br>CA-N CP: 13.7 (CA), 40 (N)<br>N-H CP: 39 (N), 92.3 (H)<br>TOCSY (DIPSI): 10	13C dec (1H t): waltz16, p=25, RF=10kHz<br>15N dec (1H t): waltz16, p=50, RF=10kHz<br>1H dec (15N t): swftppm, p=47us, RF=10kHz
hCCH TOCSY, 55.6 kHz MAS	H: 29.75<br>C1: 80<br>C2: 80	H: 7<br>C1: 17 (M) 54 (CA)<br>C2: 17 (M) 54 (CA)	H: 624/0.015<br>C1: 320/0.0114<br>C2: 320/0.0114	65	H-C CP: 3000<br>TOCSY (DIPSI): 21730	H-C CP: 92.3 (H), 40 (C)<br>TOCSY (DIPSI): 10	13C dec (1H t): waltz16, p=16.7us, RF=15kHz
CONCACX, 15 kHz MAS	CX: 389.7<br>CA: 40<br>N: 36<br>CO: 12	CX: 100<br>CA: 57<br>N: 120<br>CO: 175	CX: 2352/0.020<br>CA: 72/0.0060<br>N: 60/0.014<br>CO: 26/0.0072	26	H-CO CP: 1750<br>CO-N CP: 8000<br>N-CA CP: 8000<br>DARR CA-CX: 50000	H-CO CP: 77.9 (H), 60 (CO)<br>CO-N CP: 11.4 (CO), 4 (N)<br>N-CA CP: 4 (N), 10.1 (CA)<br>DARR CA-CX: 15 kHz (1H)	1H dec (CO-N): CW, 91.7 kHz<br>1H dec (N-CA): CW, 91.7 kHz<br>1H dec (t1s): swftppm, 91.7 kHz
CANCOCX, 15 kHz MAS	CX: 389.7<br>CA: 40<br>N: 36<br>CO: 12	CX: 100<br>CA: 57<br>N: 120<br>CO: 175	CX: 2352/0.020<br>CA: 76/0.0070<br>N: 58/0.012<br>CO: 28/0.0077	19.2	H-CA CP: 1750<br>CA-N CP: 8000<br>N-CO CP: 8000<br>DARR CO-CX: 50000	H-CA CP: 77.9 (H), 60 (CO)<br>CA-N CP: 25.7 (CA), 4 (N)<br>N-CO CP: 4 (N), 11.2 (CO)<br>DARR CO-CX: 15 kHz (1H)	1H dec (CA-N): CW, 91.7<br>1H dec (N-CO): CW, 91.7<br>1H dec (t1s): swftppm, 91.7 kHz
3D HMBC-CC-HMQC	H: 13<br>C(met): 20<br>C(aliph): 80	H: 4.7<br>C(met): 20<br>C(aliph): 43.5	H: 1034/0.0496<br>C(met): 360/0.0447<br>C(aliph): 160/0.005	68	H-C(remote) INEPT: 32 ms (4*1/125Hz)<br>C-C INEPT: 7.2 ms<br>H(met)-C(met): 3.65 ms	not applicable for INEPT transfers	13C dec during acq: 5 kHz WALTZ65<br>1H dec during 13C dim: 1H 180° pulse

Table S4 (continued)

**Experiment**	**SW (ppm)**	**Carrier (ppm)**	**Acquisition points/time (s)**	**NUS (%)**	**Transfer/mixing times (us)**	**RF field strengths (kHz)**	**Decoupling**
3D hC-TOCSY-C-TOCSY-cH (l1=2)	H: 13<br>C(met): 24<br>C(aliph): 80	H: 4.7<br>C(met): 15<br>C(aliph): 45	H: 1034/0.0496<br>C(met): 250/0.0258<br>C(aliph): 434/0.013	28	H-C(met) INEPT: 3.8 ms<br>C-C TOCSY (FLOPSY16): 11.8 ms	FLOPSY16: 10 kHz 13C RF	13C dec during acq: 4.95 kHz WALTZ65<br>1H dec during 13C(met) dim: 1H 180° pulse<br>no dec during 13C(ali) dim.
3D hC-TOCSY-C-TOCSY-cH (l1=3)	H: 13<br>C(met): 24<br>C(aliph): 80	H: 4.7<br>C(met): 15<br>C(aliph): 45	H: 1034/0.0496<br>C(met): 256/0.0265<br>C(aliph): 448/0.0139	22.6	H-C(met) INEPT: 3.8 ms<br>C-C TOCSY (FLOPSY16): 17.6 ms	FLOPSY16: 10 kHz 13C RF	13C dec during acq: 4.95 kHz WALTZ65<br>1H dec during 13C(met) dim: 1H 180° pulse<br>no dec during 13C(ali) dim.
3D hC-TOCSY-C-TOCSY-cH (l1=4)	H: 13<br>C(met): 24<br>C(aliph): 80	H: 4.7<br>C(met): 15<br>C(aliph): 45	H: 1034/0.0496<br>C(met): 256/0.0265<br>C(aliph): 448/0.0139	30	H-C(met) INEPT: 3.8 ms<br>C-C TOCSY (FLOPSY16): 23.5 ms	FLOPSY16: 10 kHz 13C RF	13C dec during acq: 4.95 kHz WALTZ65<br>1H dec during 13C(met) dim: 1H 180° pulse<br>no dec during 13C(ali) dim.
3D NOESY-15N-BEST-TROSY (H^N-N-H)	H(direct): 12<br>N: 36<br>H(indirect): 12	H: 4.7<br>N: 118	H: 1364/0.0709<br>N: 160/0.0274<br>H: 400/0.0208	37.5	NOESY mixing 250 ms	not applicable	no 15N dec during acq. (TROSY-selection)
3D NOESY-HMQC (H^C-C-H)	H (direct): 13<br>C(met): 25<br>H(indirect): 12	H: 4.7<br>C(met): 15<br>C(aliph): 4.7	H (direct): 1454/0.0698<br>C(met): 220/0.0219<br>H(indirect): 400/0.0208	38	NOESY mixing 500 ms	not applicable	13C decoupling WALTZ65, 2.29 kHz
4D HMQC-NOESY-HMQC (H-H-C-C)	H(direct): 12<br>H(indirect): 4<br>C: 23.5<br>C: 23.5	H: 0.8<br>C: 16	H(direct): 1150/0.0598<br>H(indirect): 80/0.0125<br>C: 120/0.0127<br>C: 120/0.0127	9.7	NOESY mixing 500 ms	not applicable	13C decoupling WALTZ65, 4.5 kHz
3D NOESY-HMQC (H-H-C) MIT	H (direct): 13<br>C: 20<br>H(indirect): 4	H: 4.7<br>C: 14.5	H (direct): 1454/0.0698<br>C(met): 192/0.0238<br>H(indirect): 294/0.046	40.8	NOESY mixing 500 ms	not applicable	13C decoupling WALTZ65, 2.29 kHz
3D HMQC-NOESY-HMQC (H-C-C) MIT	H (direct): 13<br>C: 20<br>C: 4	H: 4.7<br>C: 14.5	H : 1500/0.078<br>C: 216/0.0268<br>C: 216/0.0268	35	NOESY mixing 500 ms	not applicable	13C decoupling WALTZ65, 2.29 kHz
3Q-relaxation	H(direct): 15.6<br>C: 22	H: 1.5<br>C: 15.5	H: 1226/0.049<br>C: 280/0.0236	/	relaxation delays: 2, 4, 6, 8, 11, 15, 20 and 25 ms ; NS=48	not applicable	13C decoupling WALTZ65, 2.2 kHz
15N CPMG BEST-TROSY, pseudo 3D	H: 12<br>N: 34	H: 4.7<br>N: 118	H: 1360/0.0707<br>N: 256/0.0483<br>CPMG: 13 points + reference (no delay)	/	Total CPMG delay (sum of delays on both sides of Palmer element): 20 ms	not applicable	no 15N dec (TROSY)

[*] In INEPT transfers the specified delay is for in-phase-to-antiphase; the same delay is used on the way back.

[**] In cases where both the field strength and decoupling-pulse length (pcpd) are specified, the latter was optimised; in all other cases, pcpd was set to the nominal length for this field strength, e.g. a 90° pulse for WALTZ.

Descriptor	Pearson r	p	Spearman ρ
Contact density ($R_C = 7.1$ Å)	+0.325	0.002	+0.282
Burial depth	+0.191	0.072	+0.206
Methyl-group SASA	−0.351	0.0007	−0.191
Side-chain SASA	−0.316	0.003	−0.255
Relative side-chain SASA	−0.296	0.005	−0.244
Contact model, published parameters	+0.38	3×10^{-4}	+0.37

Table S5: Correlation of S^2_{axis} with structural descriptors, evaluated for chain B of the tetramer ($n = 90$). See section S2.5.2 for details.

S4 Supplementary Figures

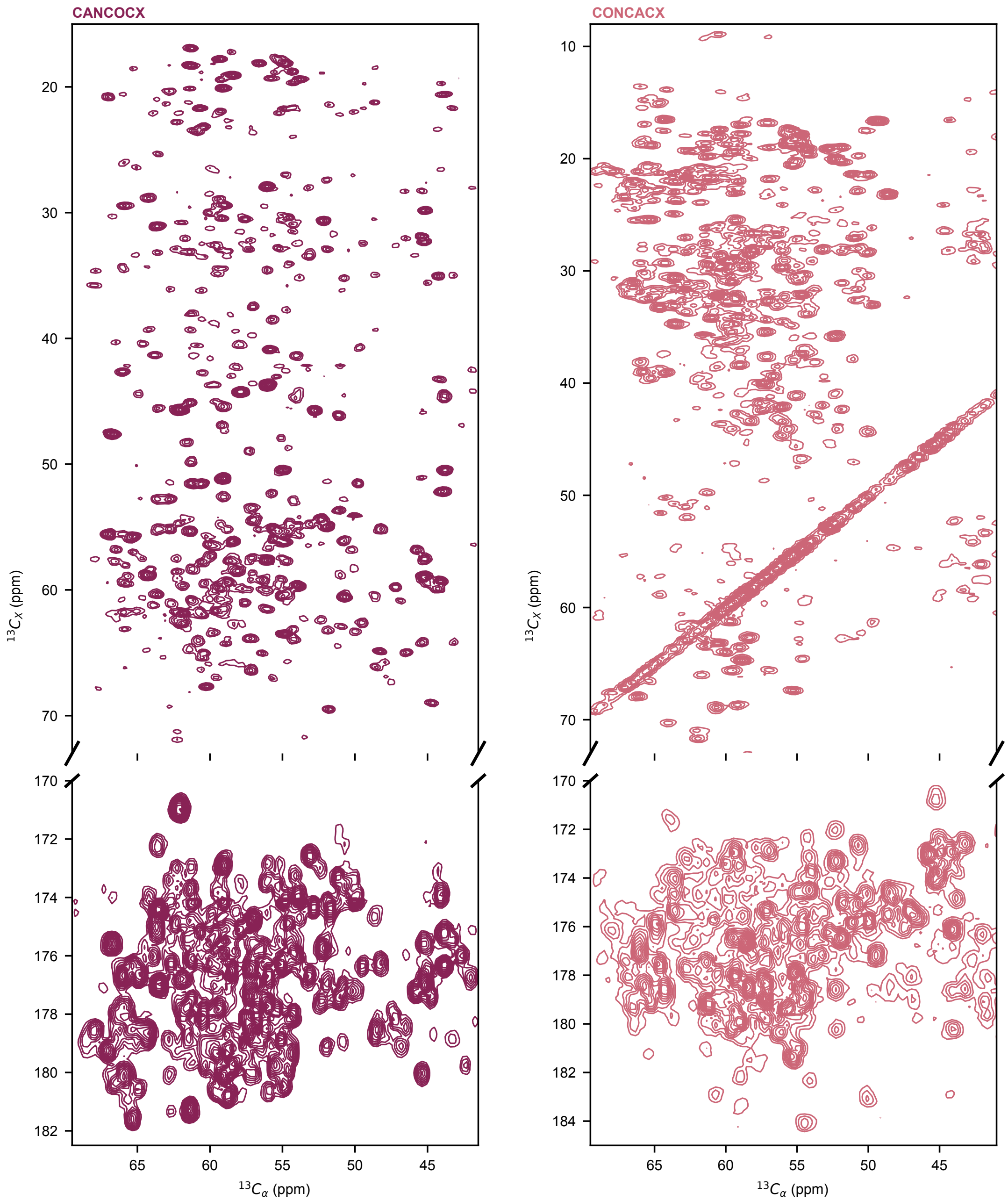

Figure S1: **^{13}C detected 4D MAS NMR spectra of sedimented *Ii*MDH recorded with a CPMAS CryoProbe.** Left: CANCOCX; right: CONCACX. Shown are projections along the dimensions N and CO.

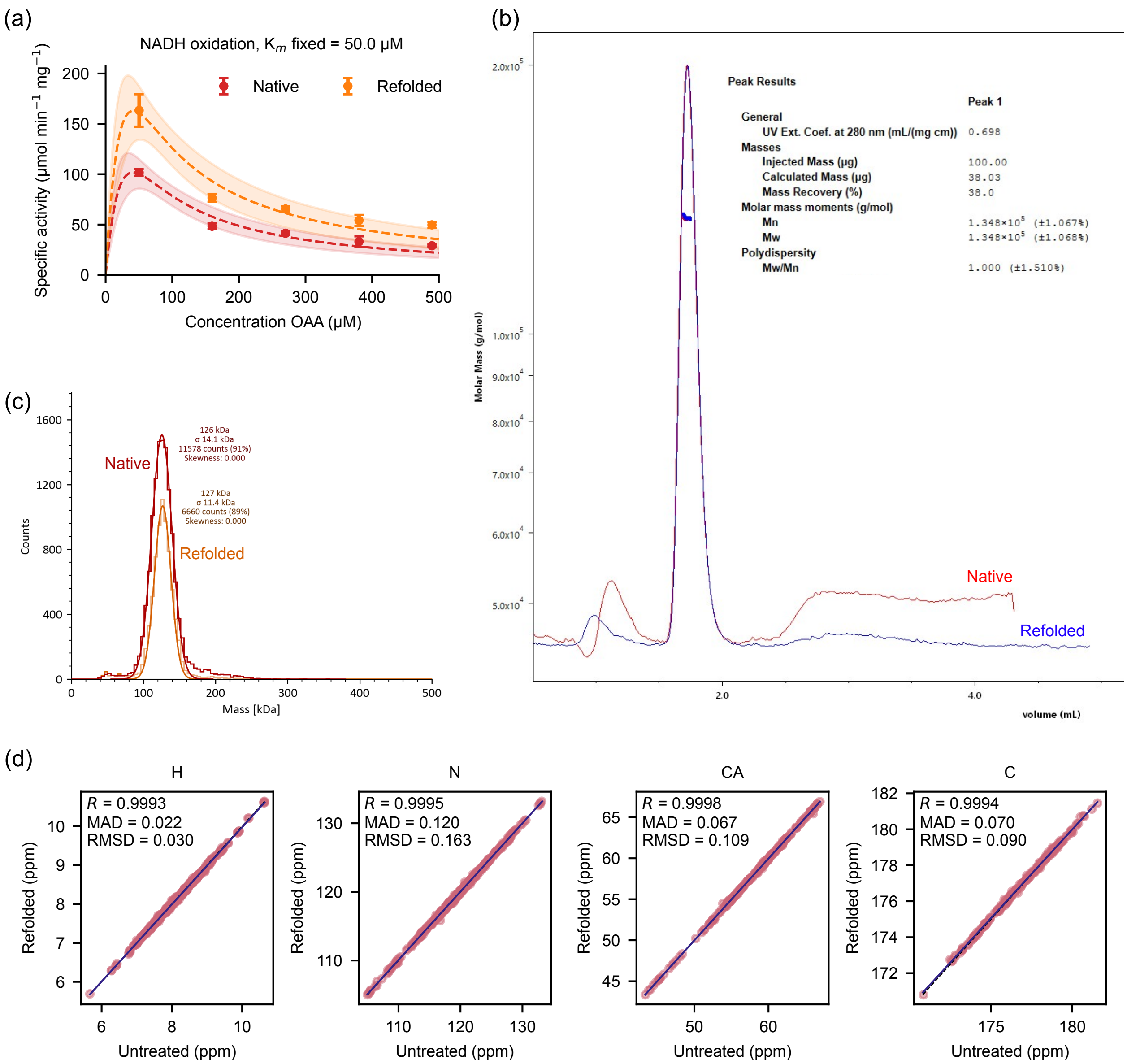

Figure S2: ***Ii*MDH refolding does not alter its activity or oligomeric state.** (a) Specific activity as a function of the oxaloacetate (OAA) concentration before and after refolding. As reported before, the enzyme exhibits strong substrate inhibition[14]. We ascribe the apparent higher activity of the refolded protein to an overestimation of the concentration of native protein due to some contaminant removed during the refolding procedure. (b) Molecular weight determined by SEC-MALS before and after refolding. (c) Molecular weight determined by mass photometry before and after refolding. (d) Comparison of chemical shifts before and after protein refolding. Chemical shifts were extracted for peaks that were visible before and after refolding in hCANH and hCONH spectra. Correlations are extremely high and differences (Mean Absolute Deviation, Root Mean Square Deviation) are within the peak linewidths, indicating that the refolding procedure did not alter the conformational landscape of the protein. Note that the untreated sample was measured on a 600 MHz spectrometer, and temperature calibration was not available. We estimate a difference of up to two degrees between the samples due to the different gas flow used (refolded: 700 MHz, set temperature 255 K, gas flow 800 liters per hour; untreated: 600 MHz, set temperature 255 K, gas flow 1000 liters per hour).

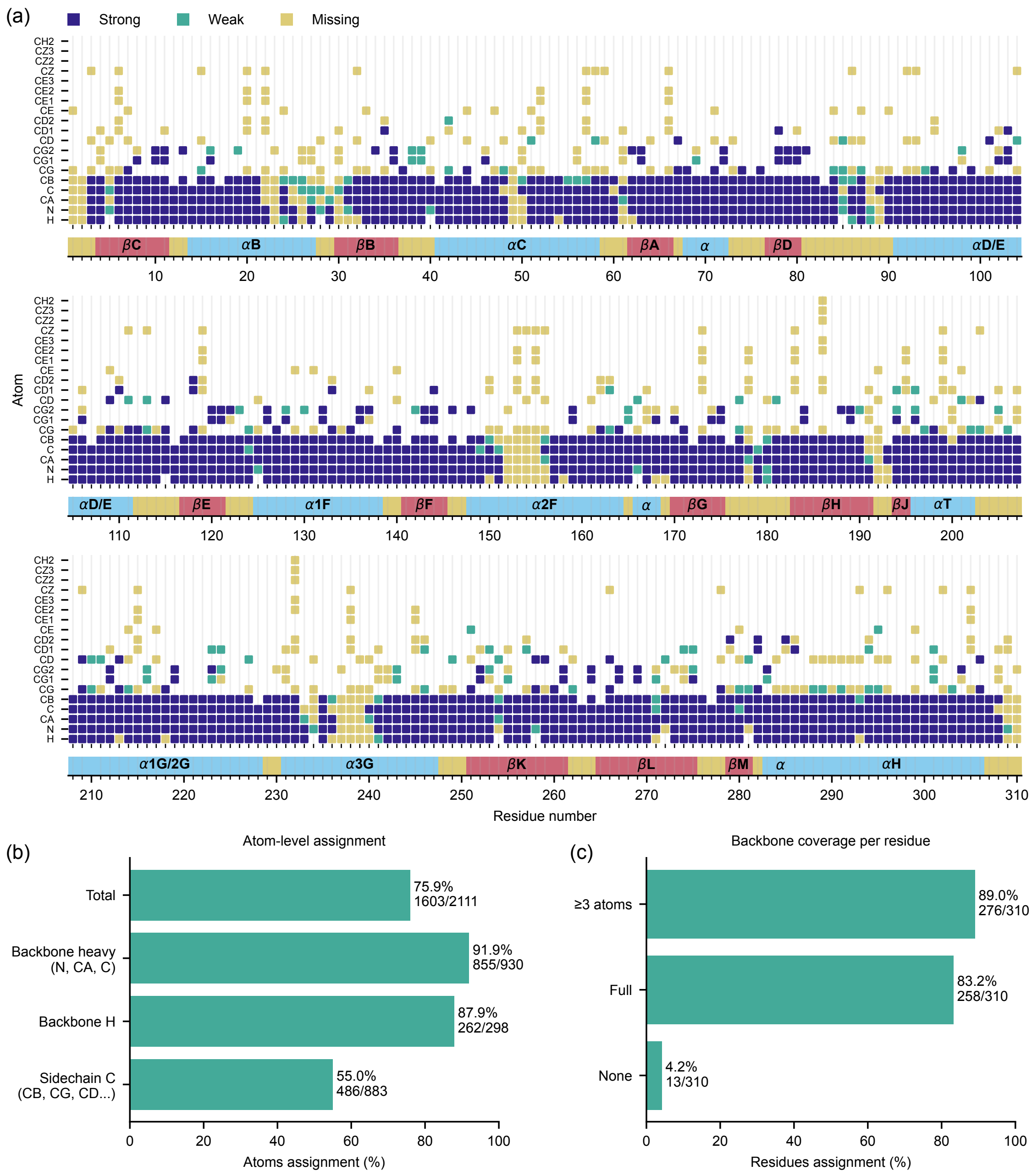

Figure S3: **MAS NMR assignment of backbone and side-chains.** (a) Extent of MAS NMR assignment. Weak assignments are supported by a single peak. (b) Assignment statistics, on a per-atom basis. (c) Assignment statistics, on a per-residue basis.

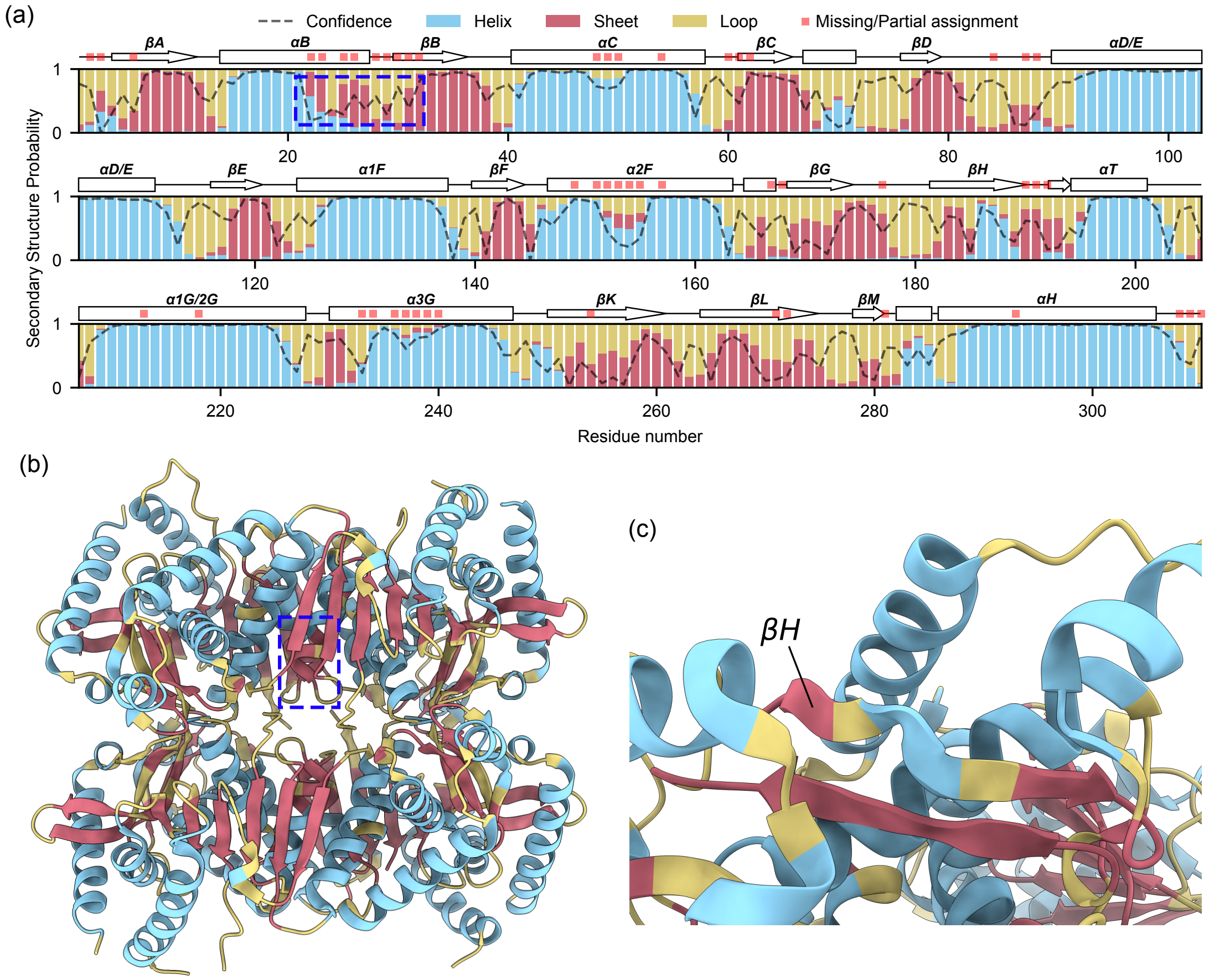

Figure S4: **Chemical shift-based prediction of secondary structures with TALOS-N[15].** (a) Secondary structure probability as predicted by TALOS-N using the MAS NMR assignments, with the prediction confidence. The secondary elements from the crystal structure (PDB: 6QSS) are reported over the stacked bar plot, as well as residues with missing/partial assignment of the backbone heavy-atoms. (b) *Ii*MDH crystal structure, colored based on the TALOS-N predictions, highlighting close agreement between the two, with the exception of helix αB (which is characterized by low confidence due to the lack of assignments). (c) Zoom on the βH strand. TALOS-N predicts a short turn in the middle of the strand, which is observed in the crystal structure too.

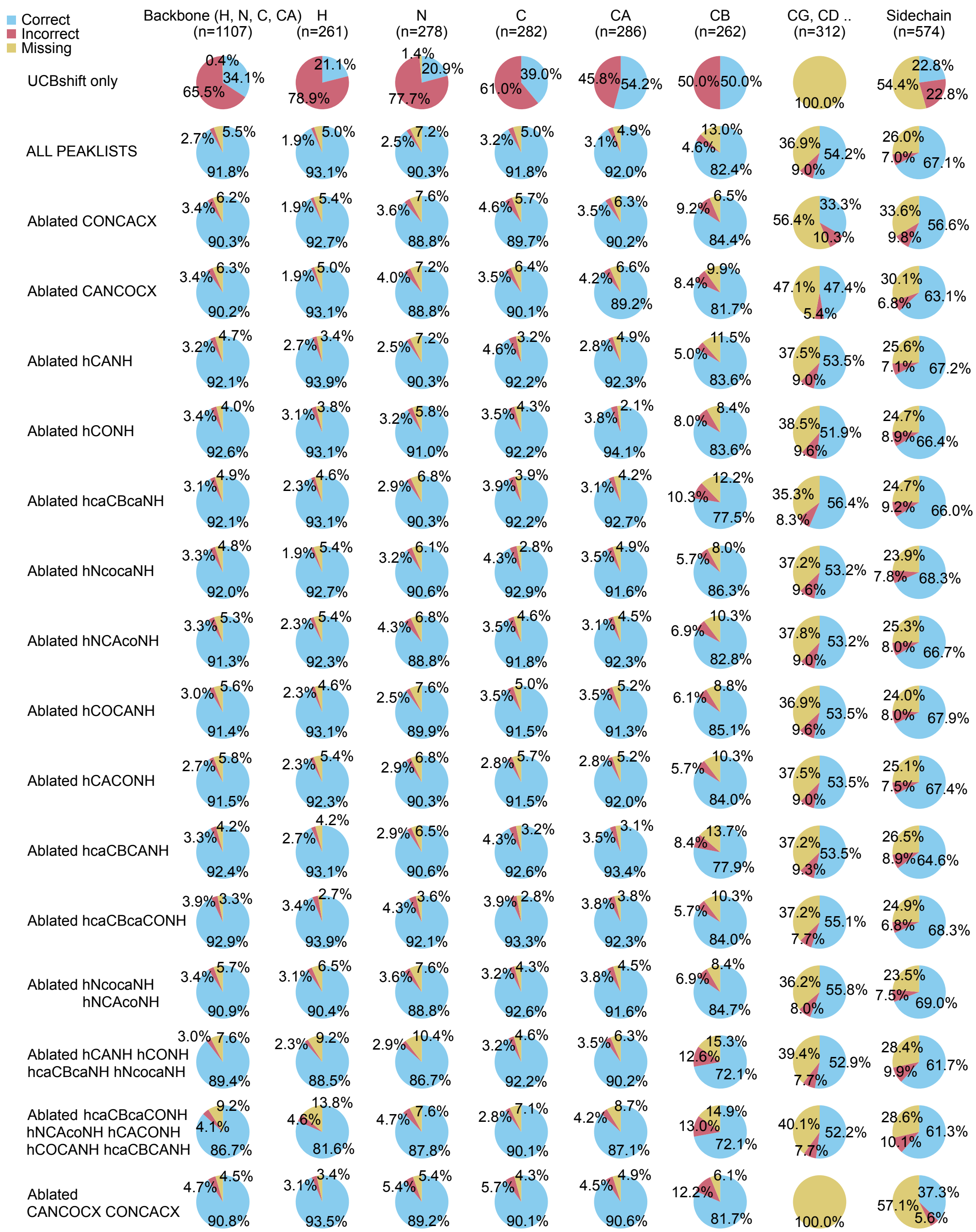

Figure S5: **Automated assignment with FLYA[16] and ablation studies.** We compared our assignment with results of FLYA runs where peaklists were omitted. Removing one or a class of peaklists does not dramatically affect the ability of FLYA to retrieve assignments, indicating a high degree of redundancy for the information contained in the different spectra. In the first row, a direct comparison of the UCBshift[1] prediction with our assignment is provided. 100% corresponds to the assignment described in this publication, and not to the totality of assignable frequencies.

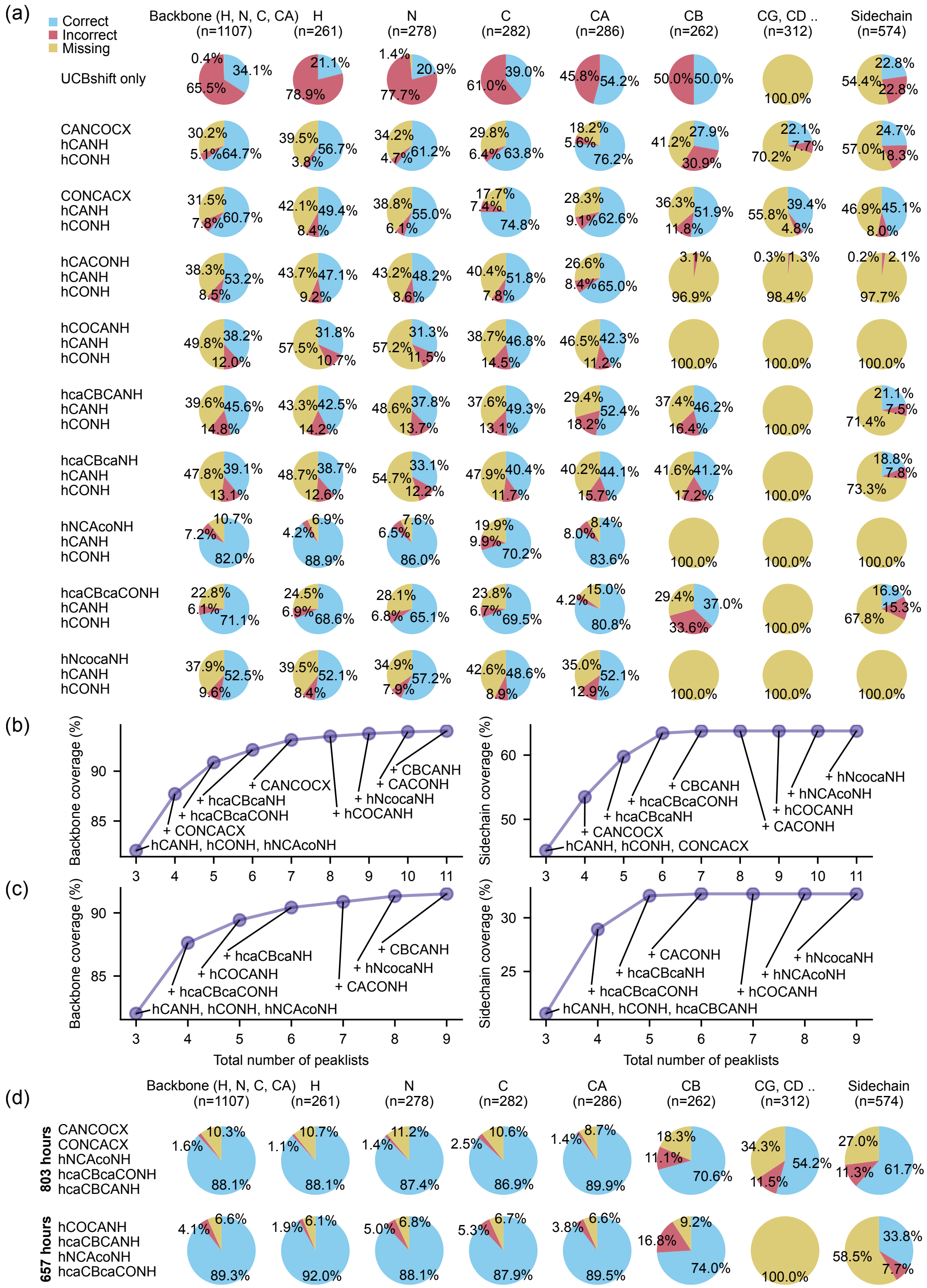

Figure S6: **FLYA addition studies and minimal datasets.** (a) We ran FLYA combining each peaklist with the ones of hCANH and hCONH (short and sensitive experiments, usually measured in the initial exploratory phases), and UCBshift predictions. (b,c) We permuted all the individual runs outputs and identified the best complementarities, depending on the aim: maximizing backbone (H, N, C, CA, left) or side-chain (CB, CG, CD..., right) assignment. In panel (c), we omitted CANCOCX and CONCACX, which rely on C-detection instrumentation. (d) Combining the results of the ablation and addition studies, we propose two combinations of spectra which could result in satisfactory assignment. The bottom option is suitable when C-detection instrumentation is not available.

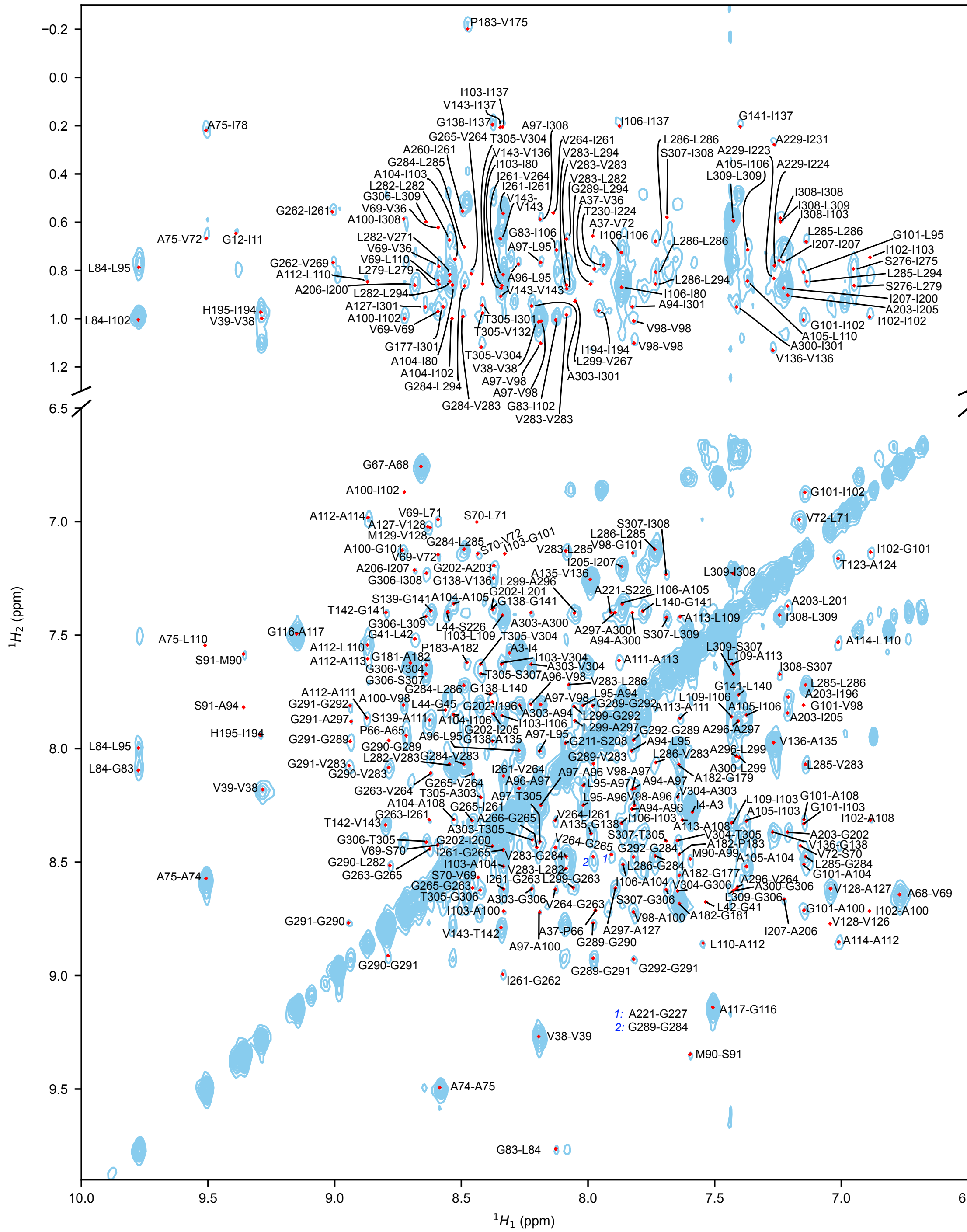

Figure S7: **H^N-(TROSY)-N-H NOESY spectrum projected along the N dimension**[17]. Through-space contacts between amide protons and amide/methyl protons can be detected in this spectrum.

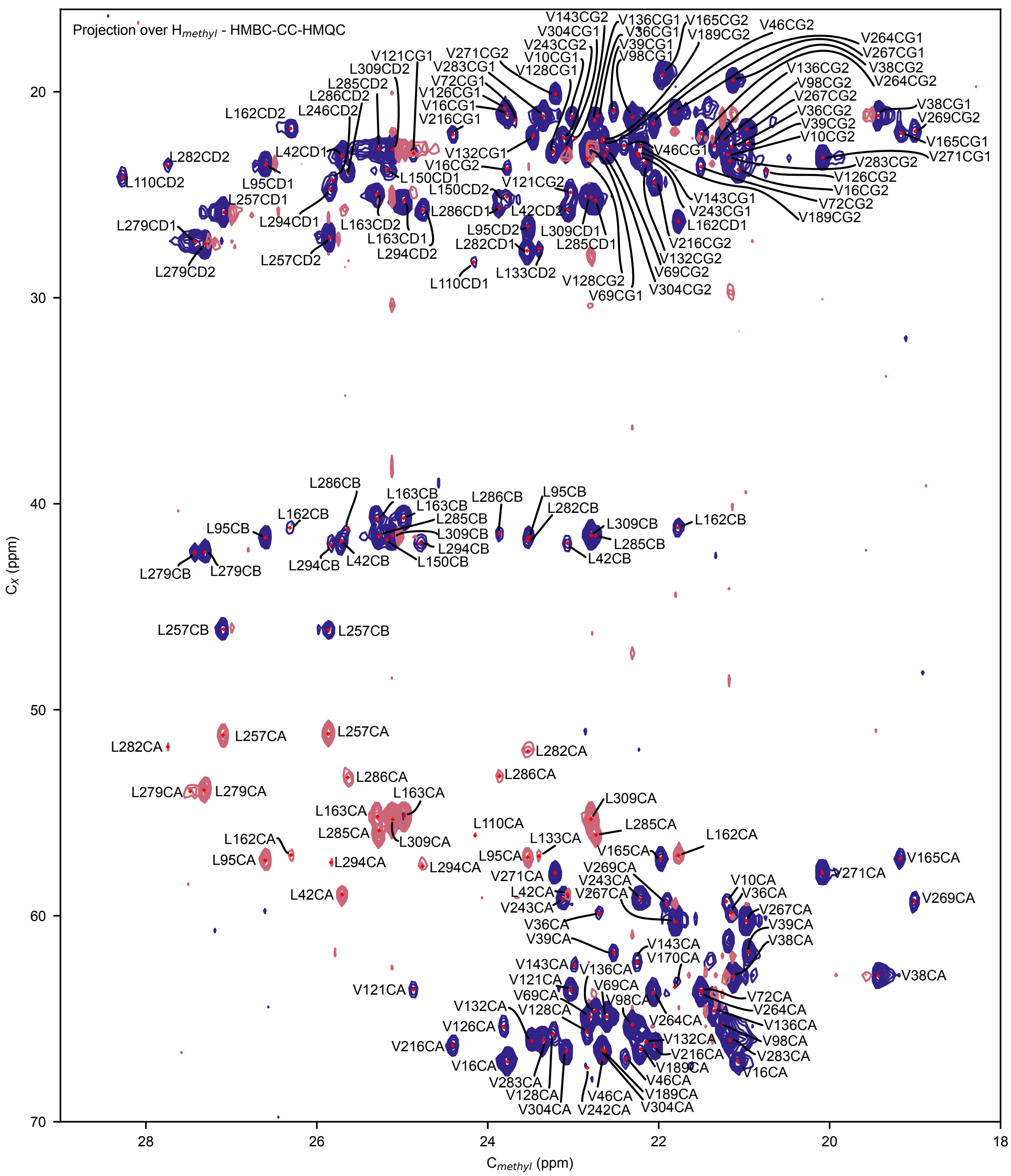

Figure S8: **HMBC-CC-HMQC spectrum projected along the H dimension.** In this spectrum, Leu-δ_1/-δ_2 and Val-γ_1/-γ_2 methyl groups can be connected – through-bond – to their C_α/C_β and C_α, respectively. The sample was produced using α-ketoisovalerate as a precursor[18].

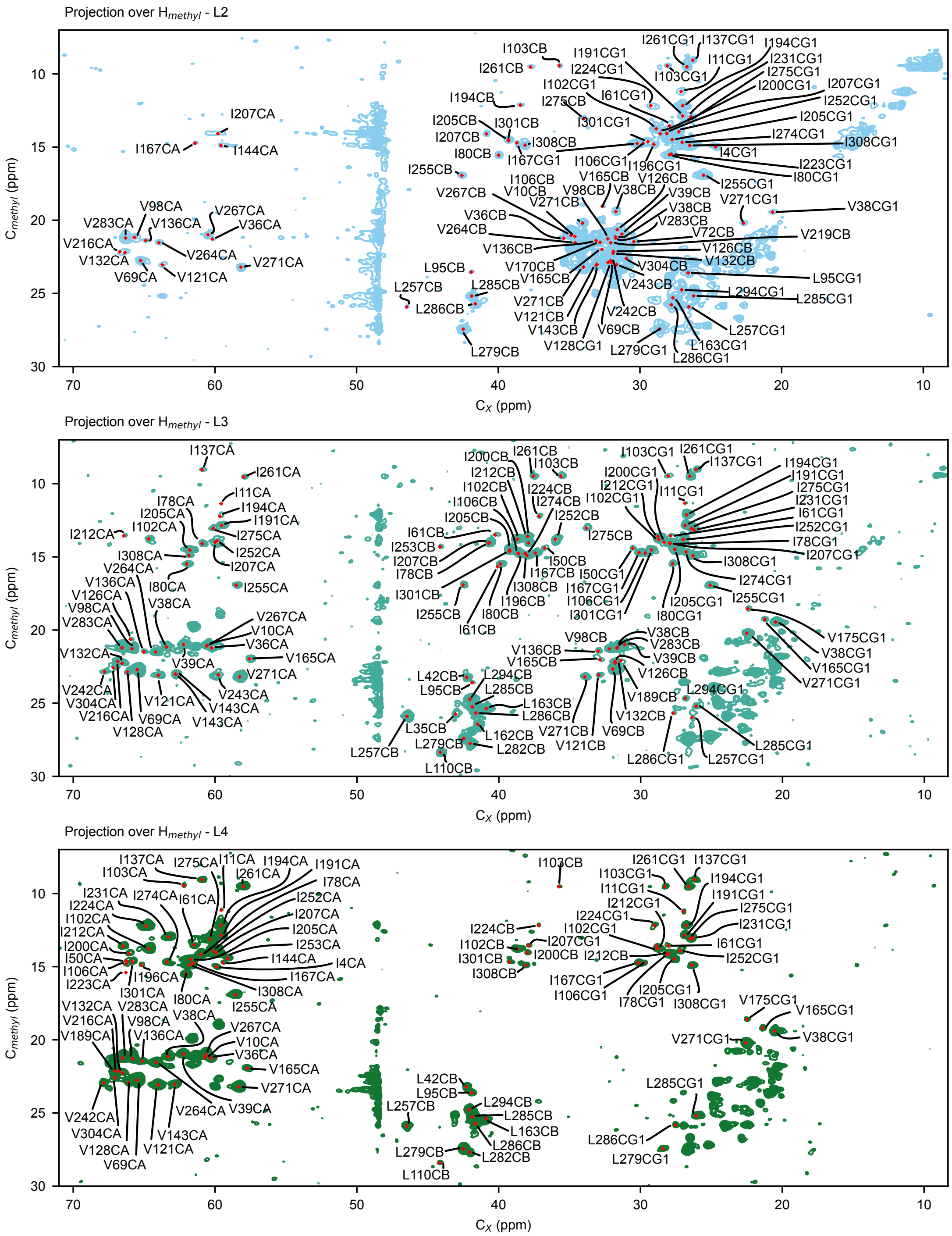

Figure S9: **TOCSY experiments projected along the H dimension.** We measured three TOCSY experiments correlating methyl groups with other carbon atoms[19]. Each experiment had a different mixing time (labeled L2, L3 and L4, corresponding to different numbers of FLOPSY cycles, ca. 11.8 ms, 17.6 ms and 23.5 ms). These resulted in different extents of transfer, favoring either the directly bonded (e.g., Ile-γ_1 in L2) or the further remote carbons (e.g., Ile-α in L4).

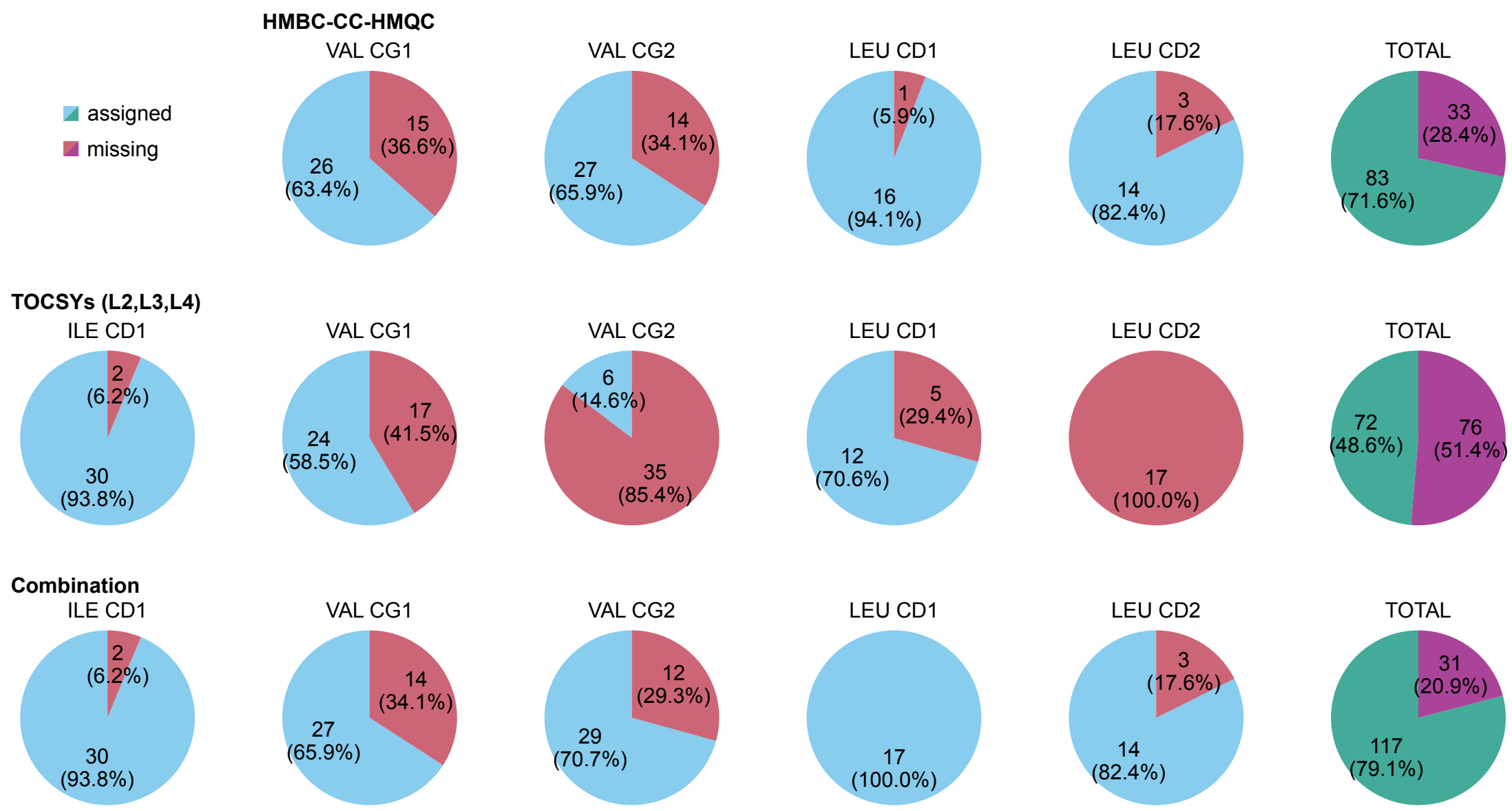

Figure S10: **Assignment of different methyl group classes in solution-NMR through-bond experiments.** The HMBC-CC-HMQC experiment recorded on the α-ketoisovalerate-labeled sample yielded good coverage of Val and Leu methyl groups. The TOCSYs experiments for the ketobutyrate/acetolactate-labeled sample perform worse (especially for Val $\gamma 2$ and Leu $\delta 2$), but nicely complement the previous approach with the Ile $\delta 1$ assignments (which are not accessible with the former labeling scheme).

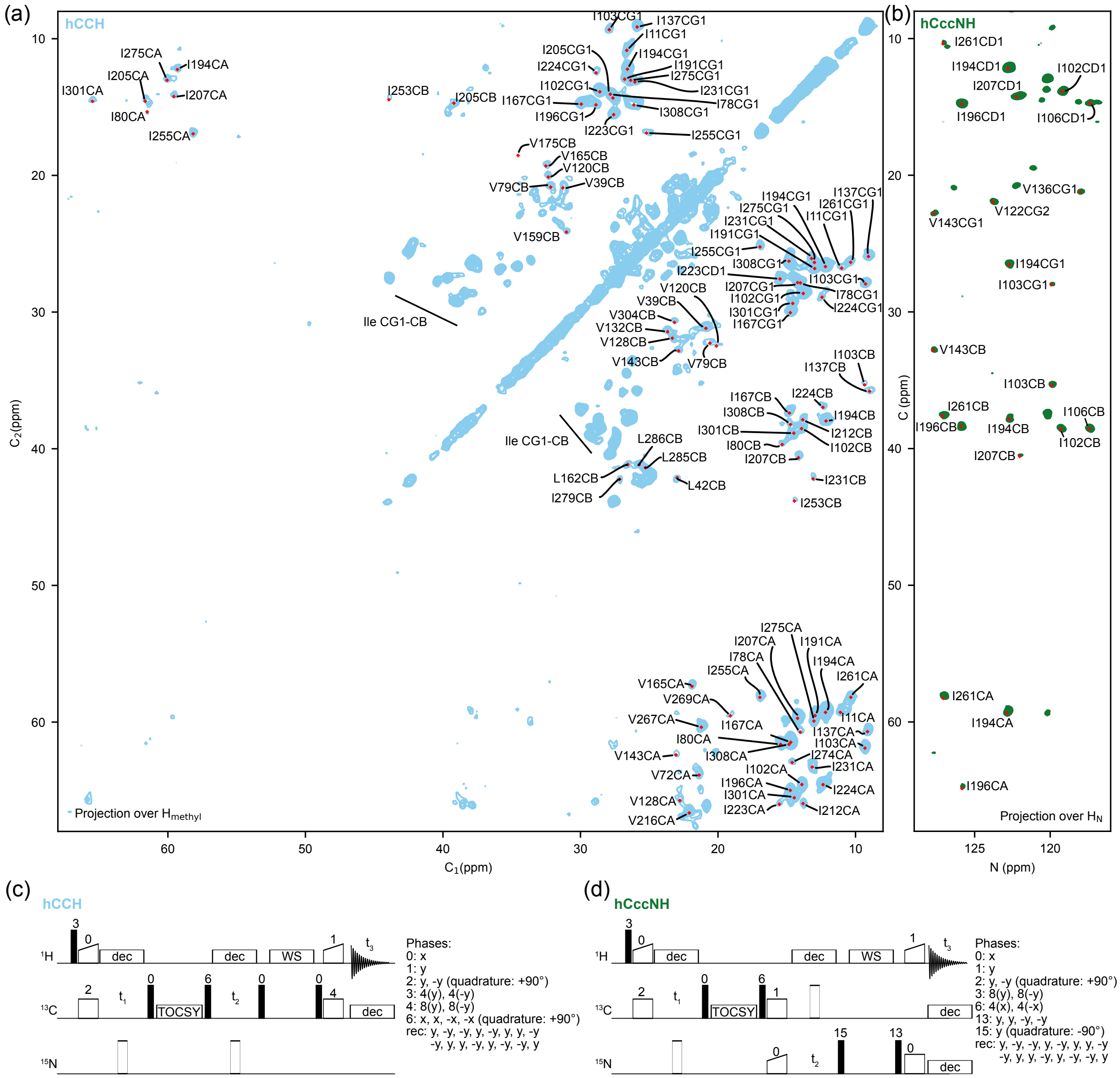

Figure S11: **TOCSY experiments for methyl assignment in the solid state**, applied to a sample with linear ^{13}C chains in Ile, Leu and Val; the sample was the same as the one for the TOCSY experiment in solution shown in Figure S9, but sedimented into a 1.3 mm rotor. (a) Projection of a 3D hCCH experiment along the methyl ^{1}H dimension. The peak labels show the identity of the non-methyl ^{13}C atom; the atom label not explicitly shown is Cδ1 (Ile, Leu) or Cγ1 (Val). (b) Projection of the hCccNH experiment, which connects the methyl carbon to the amide nitrogen and hydrogen frequencies. (c) Pulse sequence for the hCCH experiment (akin to previous implementations of HCC correlation experiments in solution[20] and in solids[21,22]) uses cross-polarization steps (indicated with a ramp on the ^{1}H channel) for transfer from ^{1}H to ^{13}C and back (3 ms, RF fields of ca. 40 kHz on ^{13}C and 92 kHz (midpoint) on ^{1}H with a 90-100 linear ramp), and a TOCSY mixing scheme for ^{13}C-^{13}C transfer (DIPSI-3[23], 21.7 ms, 10 kHz). (d) Pulse sequence for the hCccNH experiment, using cross-polarization steps (^{1}H-^{13}C: 3 ms, 40 kHz on ^{13}C, 92 kHz (midpoint) on ^{1}H with a 90-100 linear ramp; ^{13}C-^{15}N: 9 ms, 13.7 kHz on ^{13}C, 40 kHz (midpoint) on ^{15}N with a linear 90-100 ramp; ^{15}N-^{1}H: 1 ms, 91.2 kHz on ^{1}H with a linear 100-90 ramp, 39 kHz on ^{15}N), and a DIPSI-3 (21.7 ms, 10 kHz) ^{13}C-^{13}C mixing as above. Pulse sequences and parameter files are available free of charge as supplementary material with this publication.

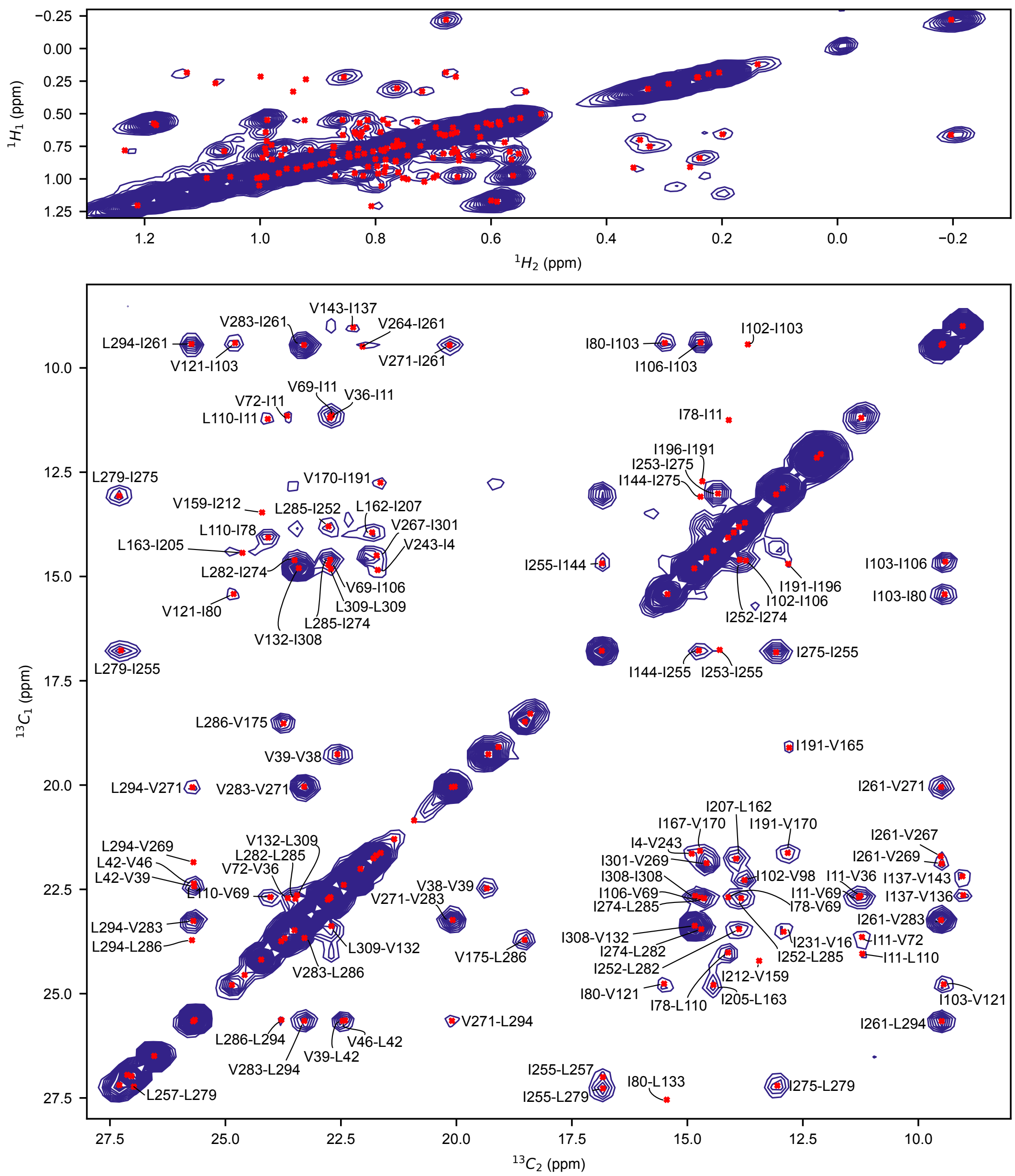

Figure S12: **H-H-C-C NOESY spectrum reporting on through-space Ile, Leu and Val methyl contacts.** Top: H-H plane (projection along the two C axes). Bottom: C-C plane (projection along two H axes).

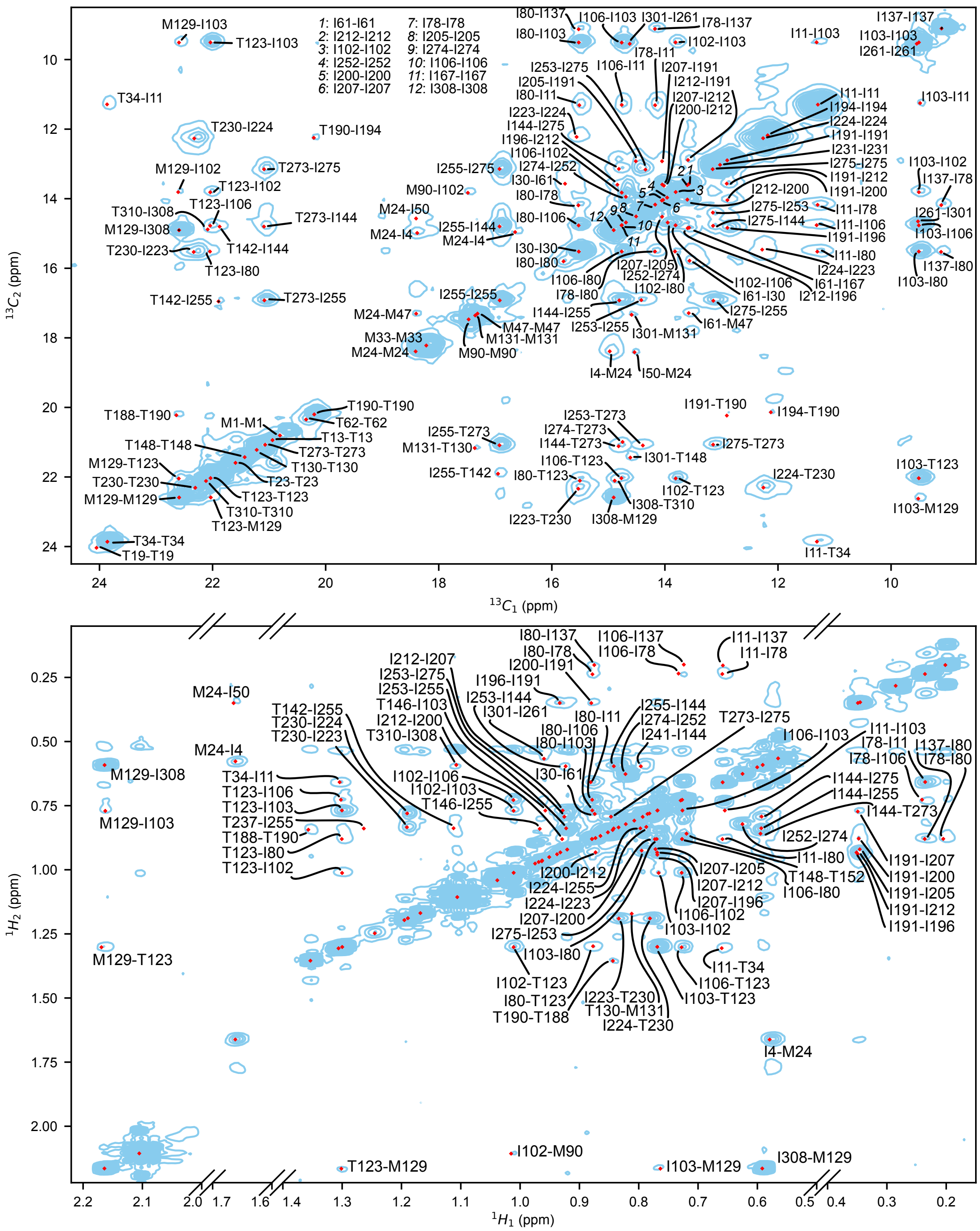

Figure S13: **NOESY spectra reporting on Ile, Met and Thr methyl contacts.** Top: H-C-C (projected along the H dimension). Bottom: H-H-C (projected along the C dimension).

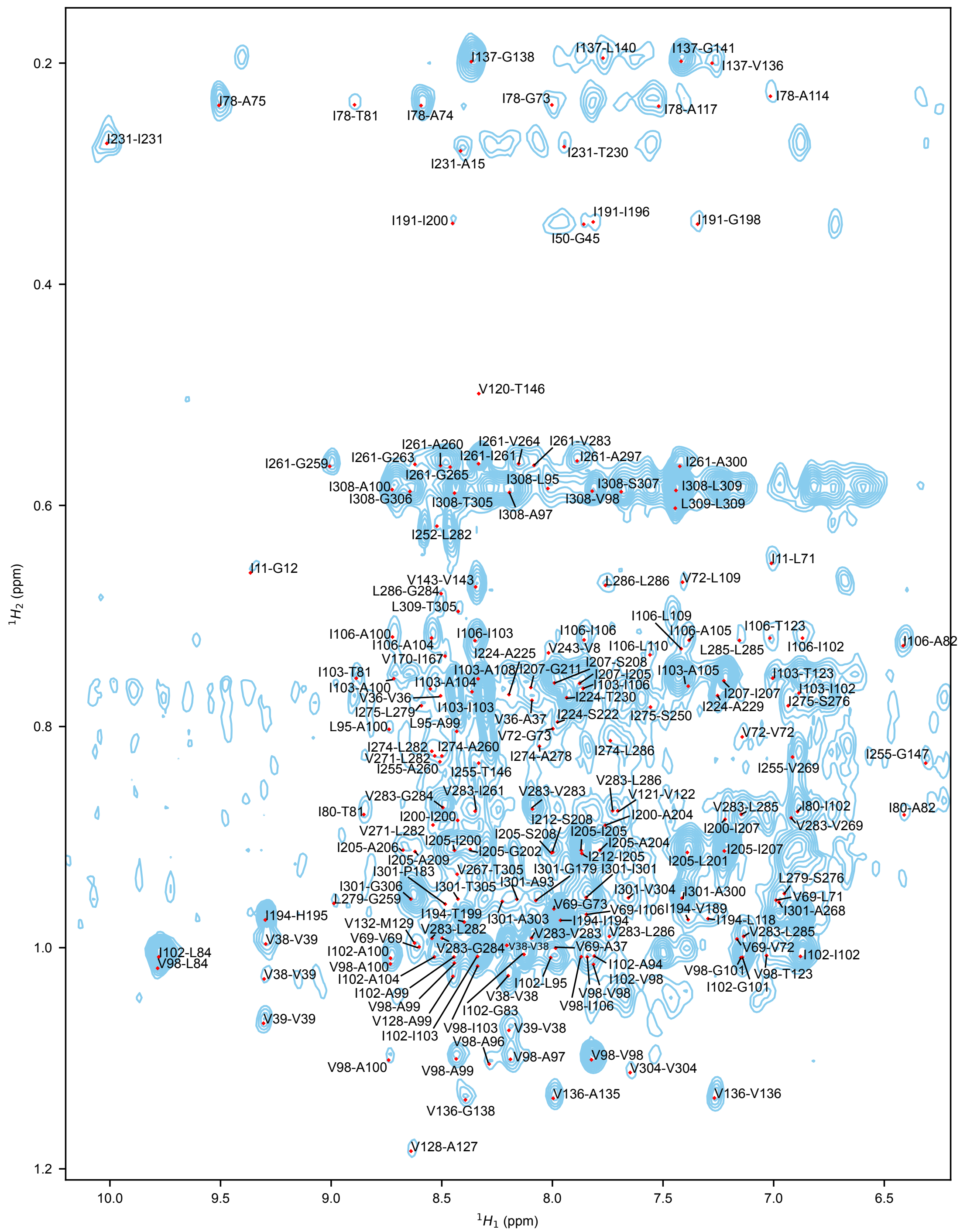

Figure S14: **H^C-C-H NOESY spectrum projected along the C dimension.** Through-space contacts between methyl and amide protons can be detected in this spectrum.

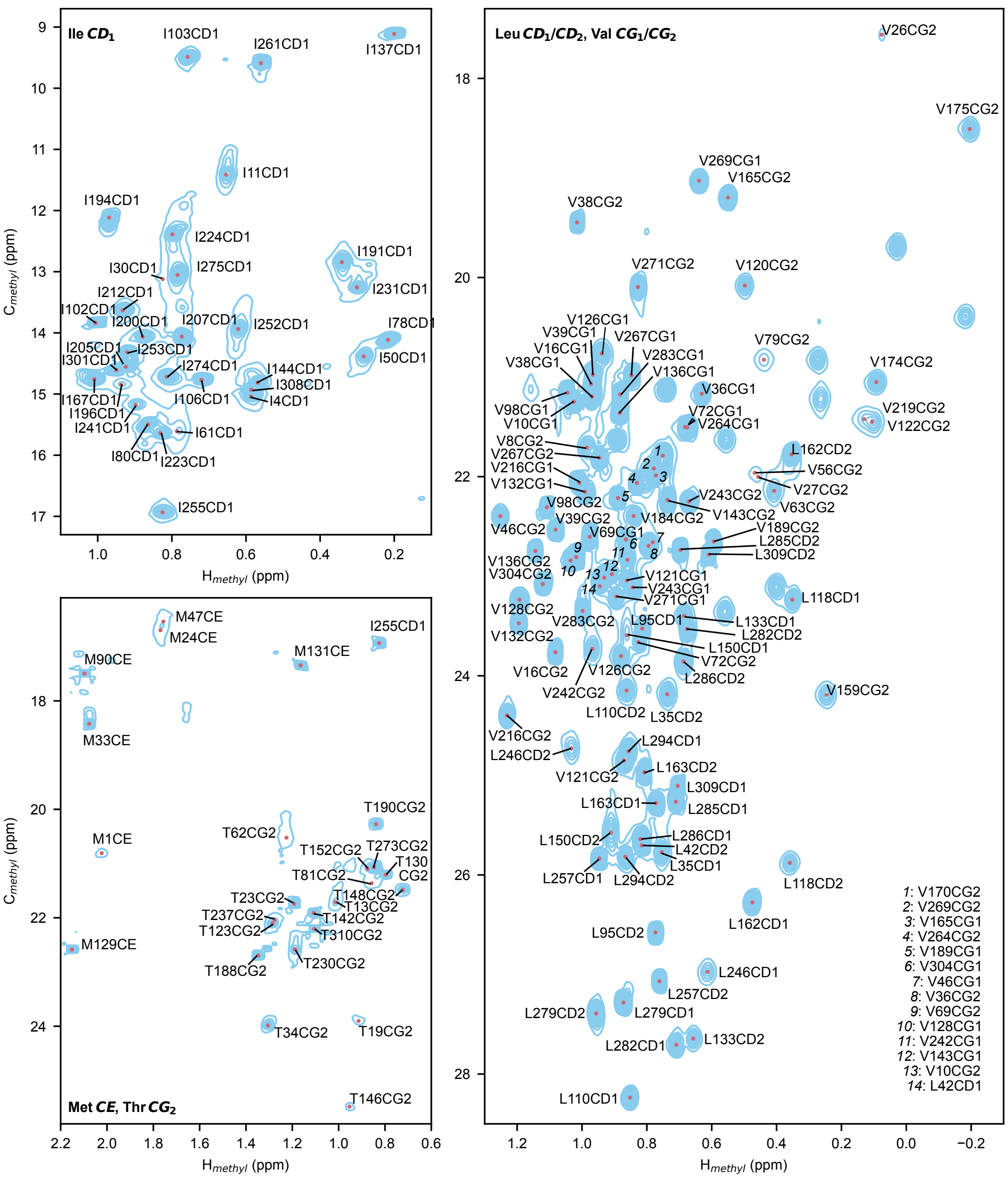

Figure S15: **Two-dimensional CH spectra of methyl groups.** Top-left: Ile-δ_1. Bottom-left: Met-ε and Thr-γ. Right: Leu-δ_1/-δ_2 and Val-γ_1/-γ_2 for mutant V168A.

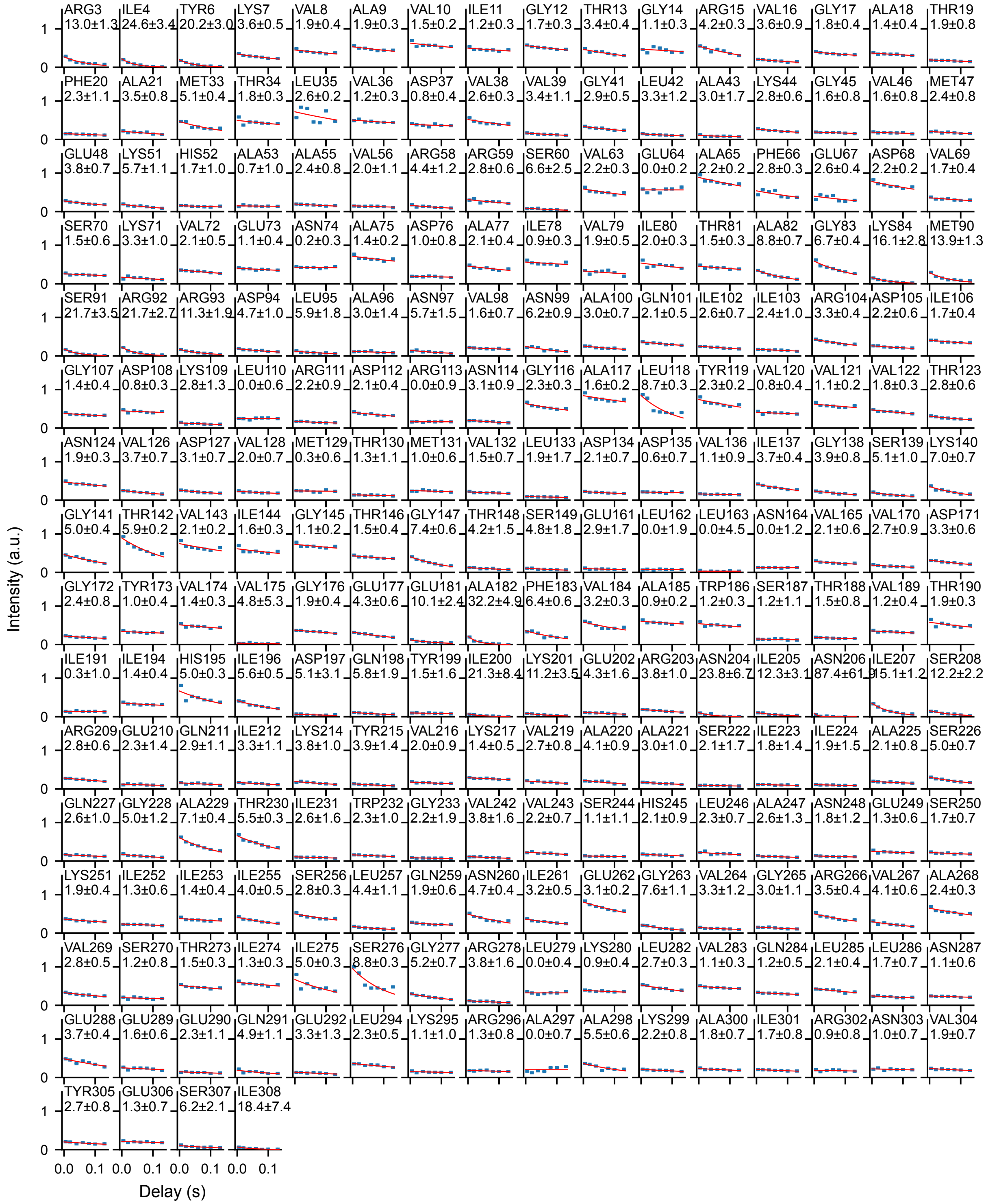

Figure S16: **MAS NMR $R_{1\rho}$ relaxation decays reporting on µs-ms dynamics.** The spinlock was set to 10 kHz; relaxation delays were 5, 20, 40, 60, 80, 100 and 130 ms.

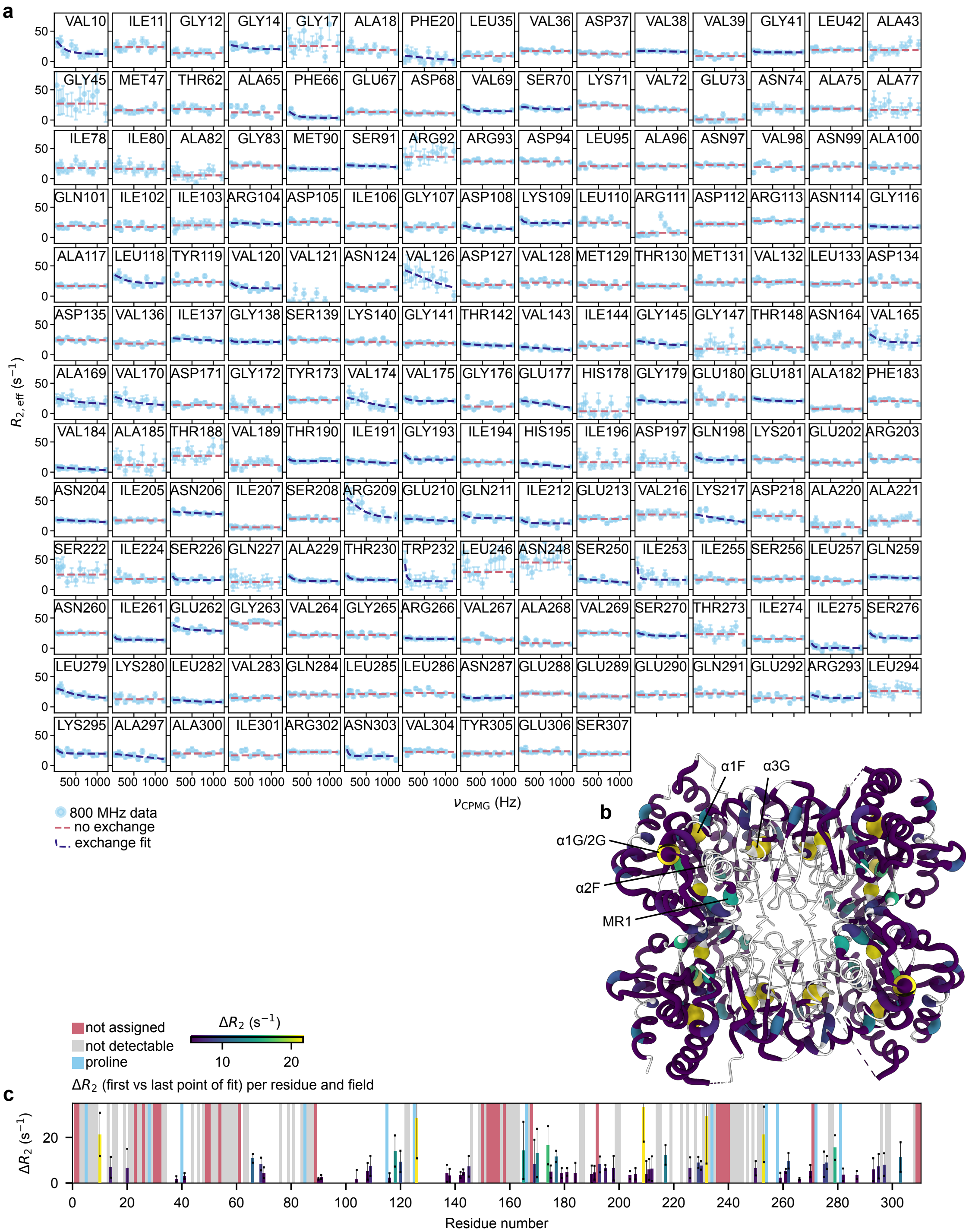

Figure S17: **^{15}N CPMG experiment of *Ii*MDH in solution.** (a) CPMG for the 190 observed amide sites, recorded at a ^{1}H Larmor frequency of 800 MHz. The dashed line shows a fit of an analytical exchange model (Luz-Meiboom[24]). The fit did not attempt to extract exchange parameters – which is essentially impossible with a single B_0 field strength – but to estimate the exchange contribution to $R_{2,eff}$. (b,c) ^{15}N $\Delta R_{2,eff}$, calculated as the difference between the first point ($\nu_{CPMG} = 50\,s^{-1}$) and last point ($\nu_{CPMG} = 1200\,s^{-1}$) of the fitted curve, plotted on the structure as worm radius and with a color gradient (b) and as a function of the residue number (c). Note that in solution, and in particular in this experiment, many more sites are undetectable. All raw data are provided in the data repository.

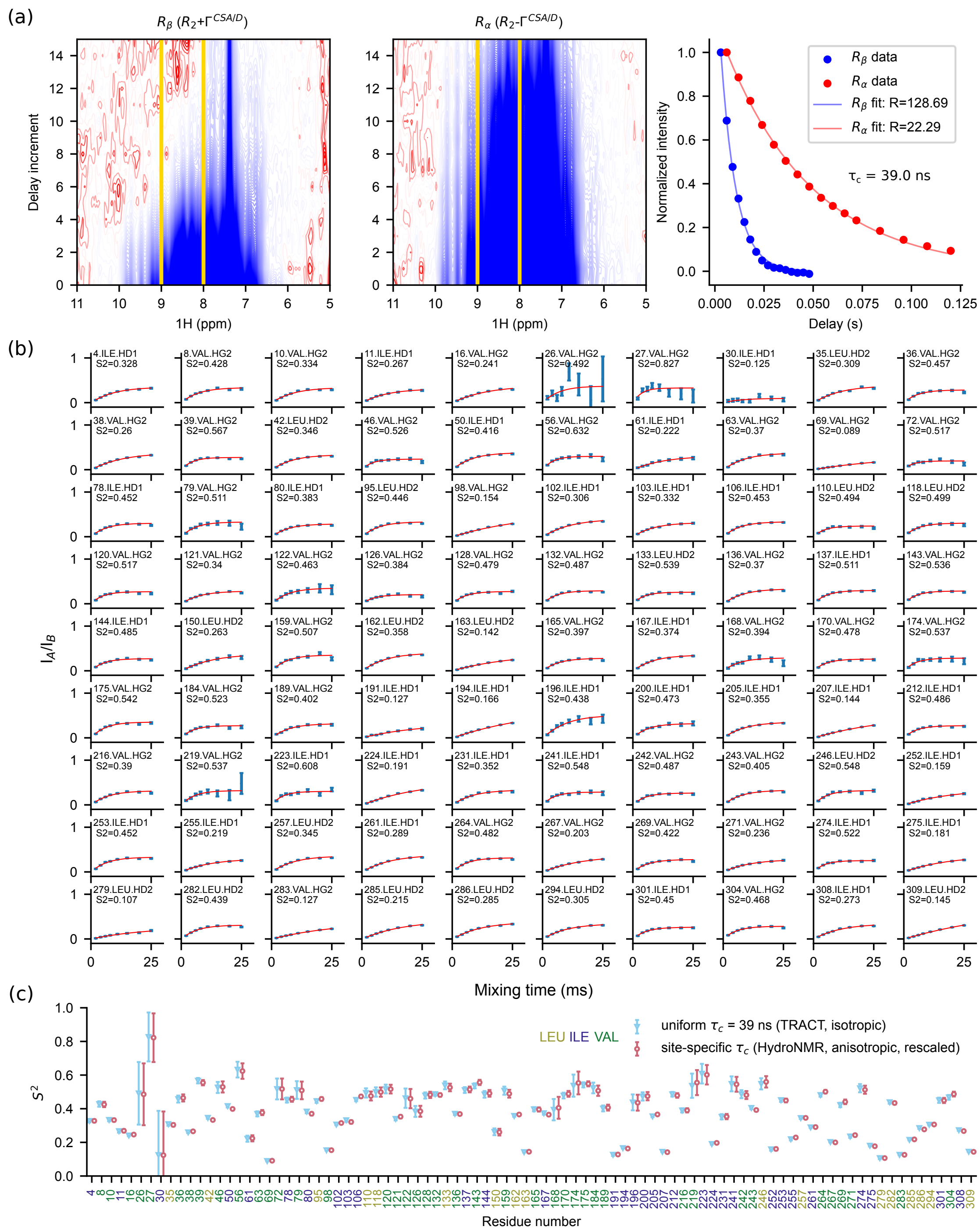

Figure S18: **^{15}N-TRACT and 3Q-relaxation buildups.** (a) $R_{2,\beta}$ and $R_{2,\alpha}$ decays with mono-exponential fits. τ_c (39.0 ns) was determined as in equation (6). (b) 3Q-relaxation buildups for Ile, Leu and Val residues. S^2 was determined as in equations (1-3). Relaxation delays were 2, 4, 6, 8, 11, 15, 20 and 25 ms. (c) Determination of the methyl-axis order parameters from the 3Q experiment, using either the assumption of a uniform overall-tumbling correlation time (blue), or using site-specific values of the effective tumbling correlation time (magenta). The latter has considered the orientation of each methyl axis (equal to the C-C axis) with respect to the tumbling tensor. Note that the error bars do not consider the uncertainty in τ_c, but only the experimental error in the fit of the 3Q data. The reasoning is that uncertainty in the overall tumbling (when treated isotropically) only scales all values; as site-to-site variations are more relevant than the overall level, conflating experimental errors of the 3Q experiment with uncertainties on τ_c would mask the statistically relevant site-to-site variations of S^2_{axis}. See supplementary text, section S2, for details.

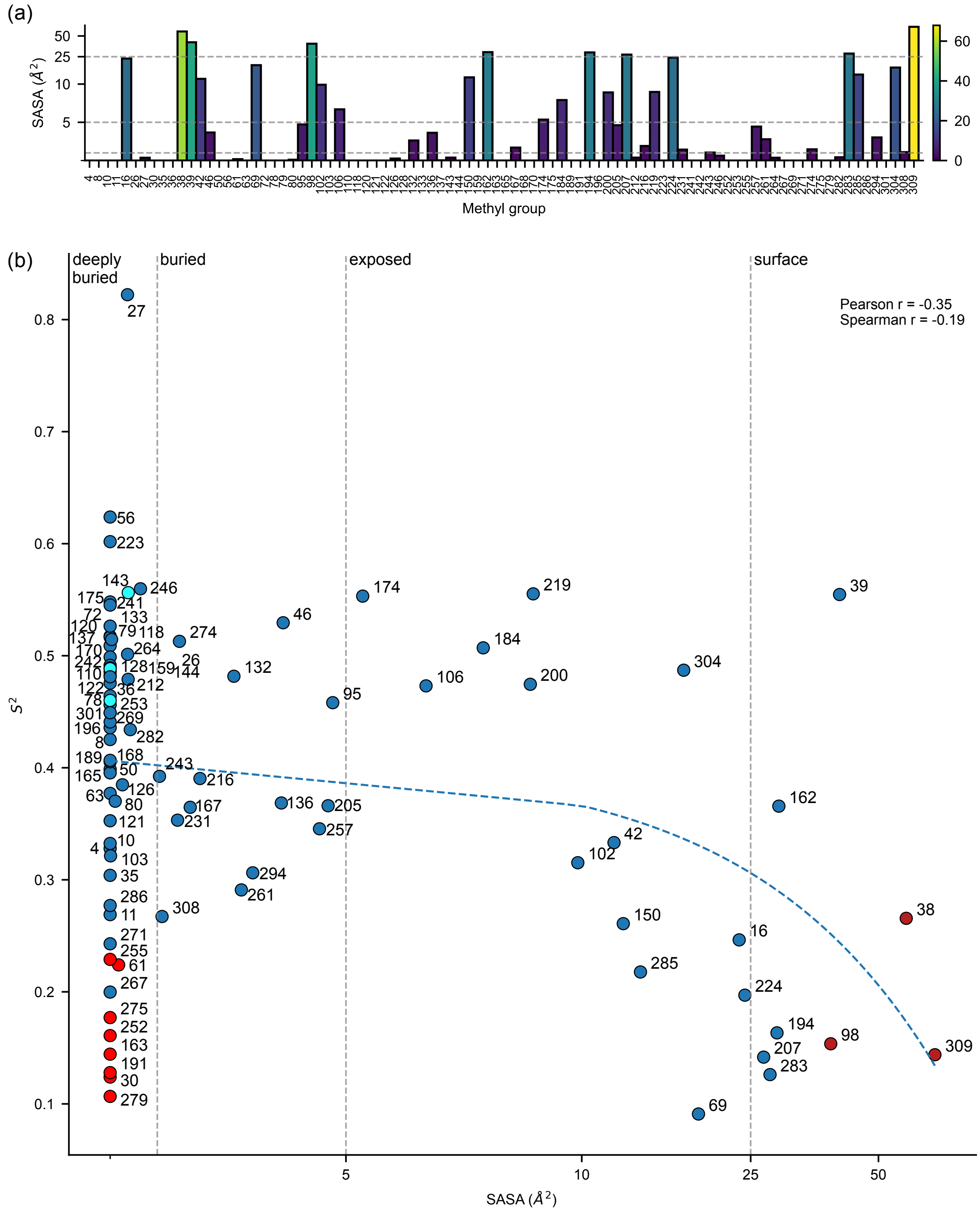

Figure S19: **Correlating Solvent Accessible Surface Area (SASA) and methyl order parameters.** (a) SASA for the methyl groups of Ile, Val and Leu in *Ii*MDH, calculated for the crystal structure (PDB: 6QSS) using the Python module FreeSASA[10]. The vertical axis uses a symlog scale with linear threshold at SASA = 10 Å^2. (b) Correlation between methyl-groups SASA and S^2. Some predictable cases of residues in the core (cyan) and on the surface (maroon) are highlighted, as in Figure 3d. The notable exceptions of deeply buried residues with low order parameters highlighted in the main text (Ile30, Ile61, Leu163, Ile191, Ile252, Ile255, Ile275 and Leu279) are shown in red. The horizontal axis uses a symlog scale with linear threshold at SASA = 10 Å^2.

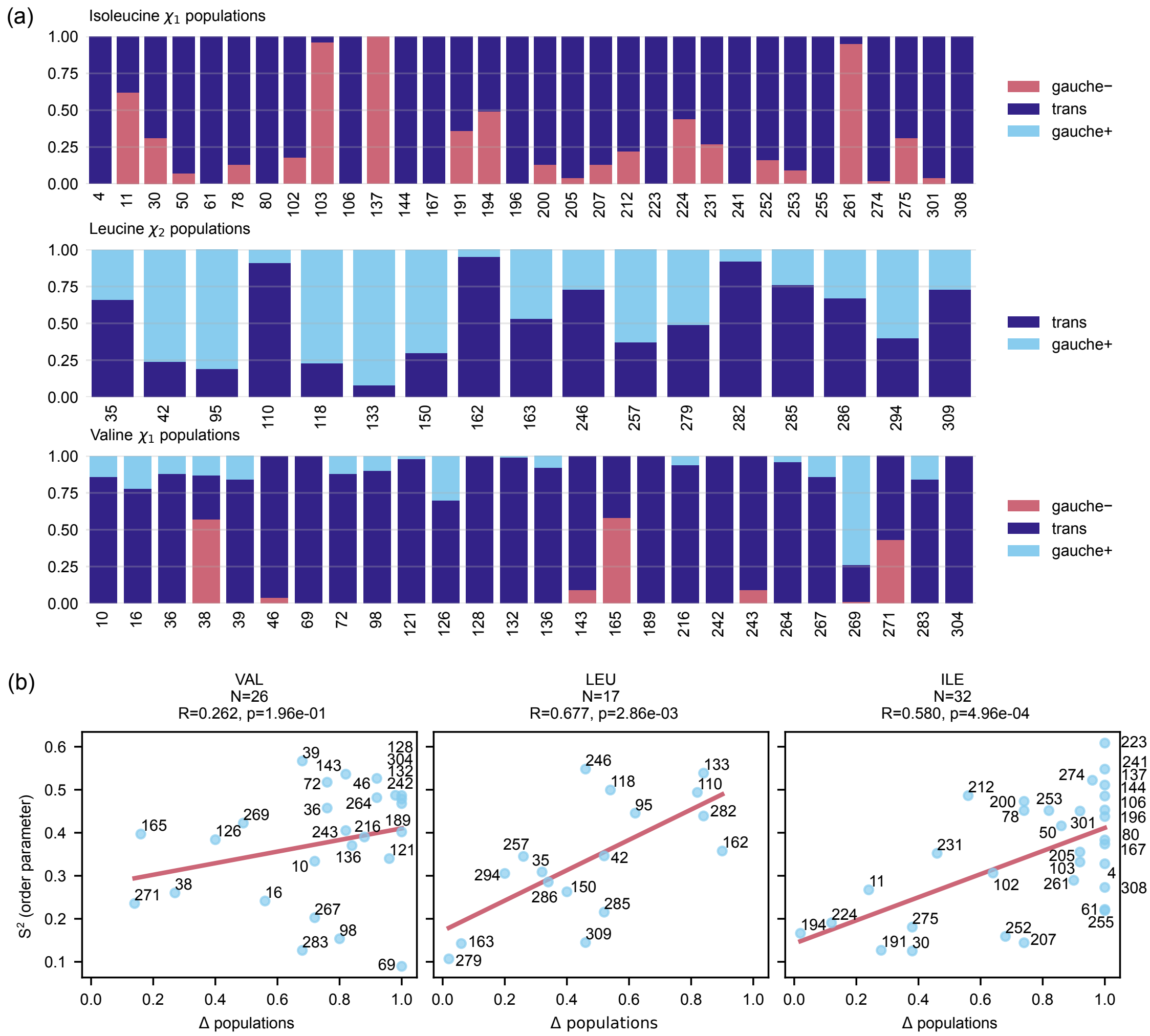

Figure S20: **Chemical shift-based prediction of rotamer populations.** (a) Isoleucine χ_1, Leucine χ_2 and Valine χ_1 populations, predicted based on the ^{13}C chemical shift of methyl groups[25–28]. (b) Correlation between methyl groups order parameters determined by 3Q-relaxation and the differences in populations between the two most populated rotamer states.

(a)

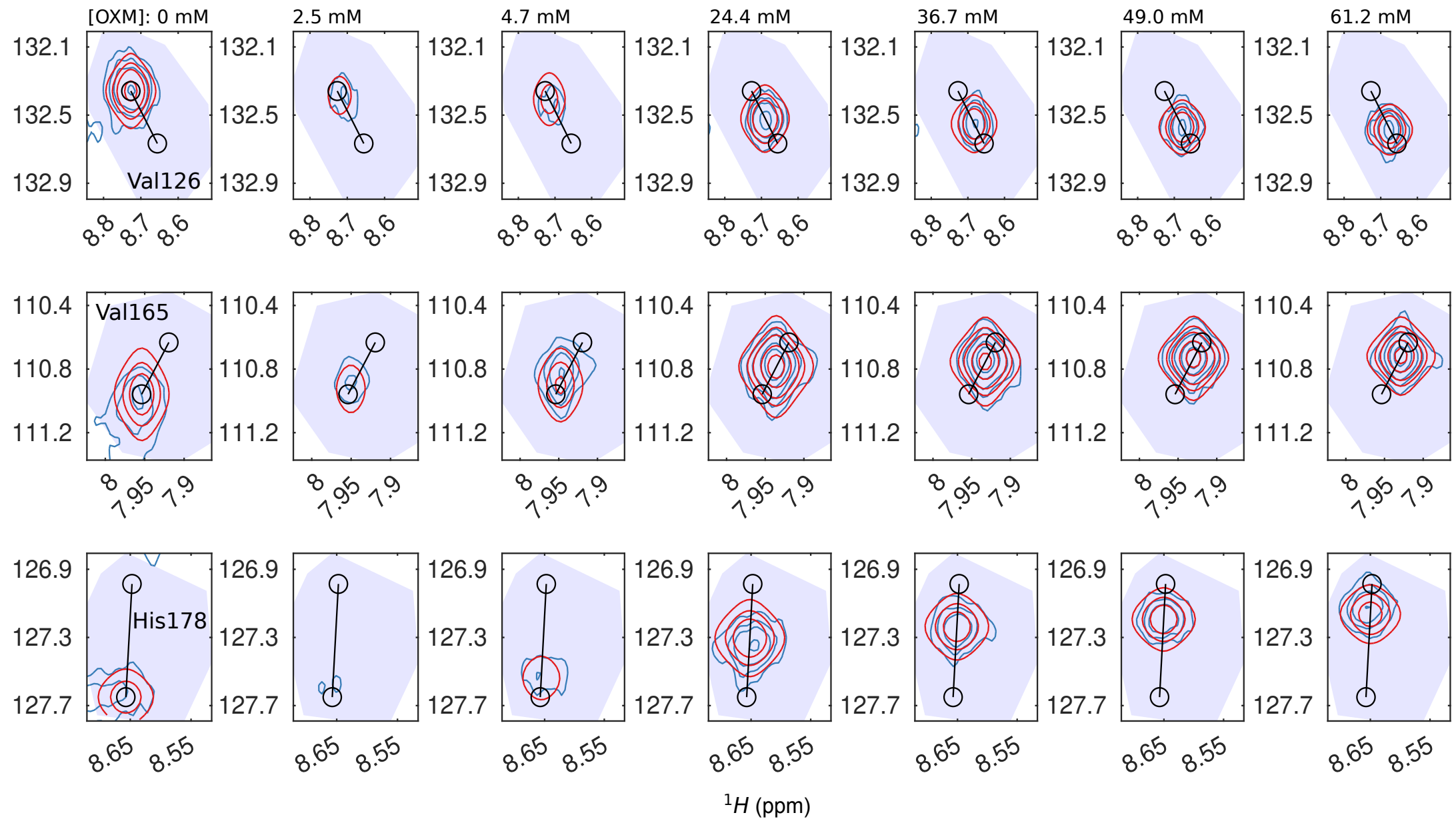

(b)

Two-state ligand binding

$P + L \rightleftharpoons PL$

P_0: protein concentration (e.g. μM)
L_0: ligand concentration (e.g. μM)

K_d: dissociation constant (e.g. μM)
k_{off}: dissociation rate (s^{-1})

(c)

ID	Parameter	Fit	Error
1	Kd	22484.265	1432.604
2	koff	999.080	173.500
3	Val126_dI_1	8.727	0.001
4	Val126_dI_2	8.656	0.002
5	Val126_dS_1	132.359	0.005
6	Val126_dS_2	132.666	0.007
7	Val126_R2I_1	196.844	5.538
8	Val126_R2I_2	201.683	6.325
9	Val126_R2S_1	40.854	2.661
10	Val126_R2S_2	3.341	3.185
11	Val165_dI_1	7.954	0.001
12	Val165_dI_2	7.920	0.001
13	Val165_dS_1	110.959	0.009
14	Val165_dS_2	110.633	0.008
15	Val165_R2I_1	109.110	3.964
16	Val165_R2I_2	84.458	3.862
17	Val165_R2S_1	88.179	7.040
18	Val165_R2S_2	14.112	4.978
19	His178_dI_1	8.658	0.002
20	His178_dI_2	8.647	0.002
21	His178_dS_1	127.650	0.010
22	His178_dS_2	126.989	0.014
23	His178_R2I_1	215.642	9.448
24	His178_R2I_2	182.781	9.210
25	His178_R2S_1	55.778	6.799
26	His178_R2S_2	27.885	6.114

(d)

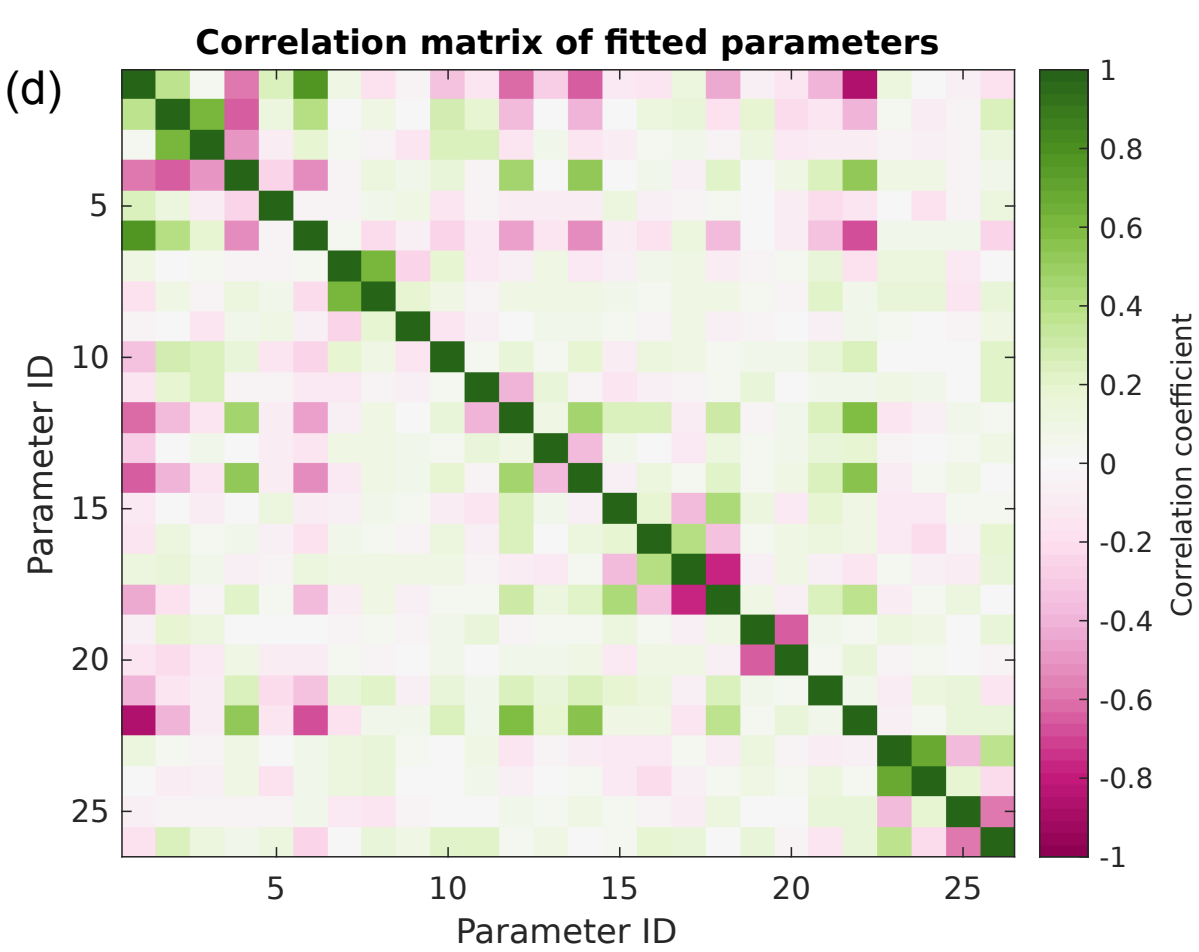

Figure S21: **Two-dimensional lineshape analysis for K_d estimation with NMR-TITAN[29].** (a) Raw spectra and NMR-TITAN fits for Val126, Val165 and His178, throughout the oxamate titration. The three residues were chosen due to their large CSP and their belonging to different structural elements (α1F, Mobile Region 1 and catalytic loop). Leu246, which was shown in Figure 4, could not be fit due to its low intensity throughout the titration. (b) A two-state ligand binding model was chosen to fit the dissociation constant (K_d, in µM) and rate (k_{off}, in s^{-1}). (c) Fitted parameters and errors (bootstrapping method, 100 replicates). In addition to the kinetic parameters, chemical shifts of free and bound states, as well as their linewidths were fitted. (d) Correlation matrix of fitted parameters after bootstrap error analysis.

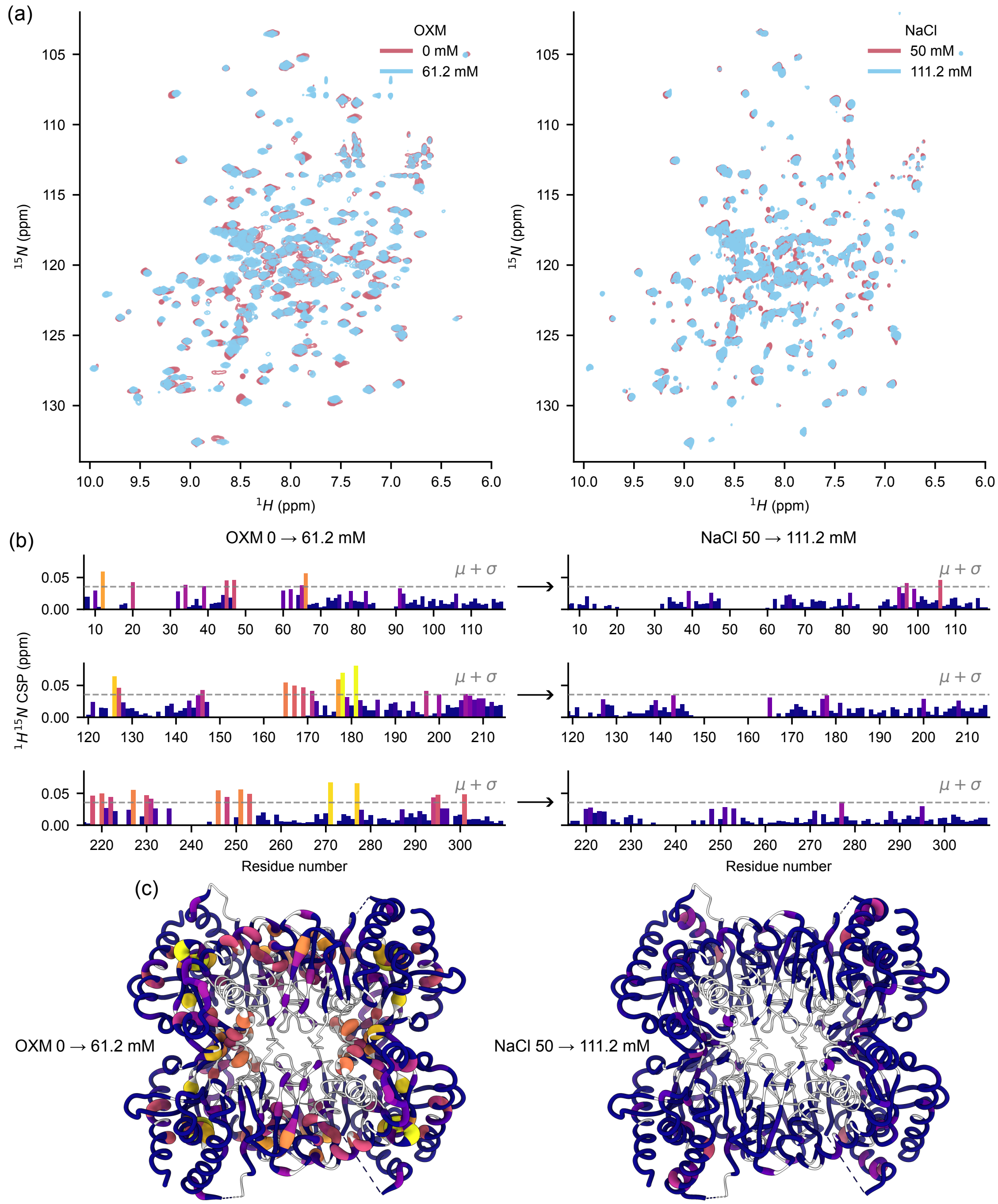

Figure S22: **Ionic strength control for the oxamate titration.** (a) Overlay of 2D NH spectra in the absence and presence of 61.2 mM OXM (left) and in the presence of 50 mM or 111.2 mM NaCl (right). The two pairs of spectra have the same ionic strength. (b,c) Composite NH CSP upon the addition of 61.2 mM OXM (left) or NaCl (right), as a function of the residue number (b) and plotted on the structure (c). While there are detectable effects of varying the ionic strength, these are not sufficient to explain the changes caused by oxamate. Almost none of the residues are significantly perturbed (when adopting the threshold used in Figure 4, $\Delta\delta_{\mathrm{NH,OXM}} > \overline{\Delta\delta_{\mathrm{NH,OXM}}} + \sigma_{\Delta\delta_{\mathrm{NH,OXM}}}$). Interestingly, helix αD/E is more perturbed by chloride than by oxamate.

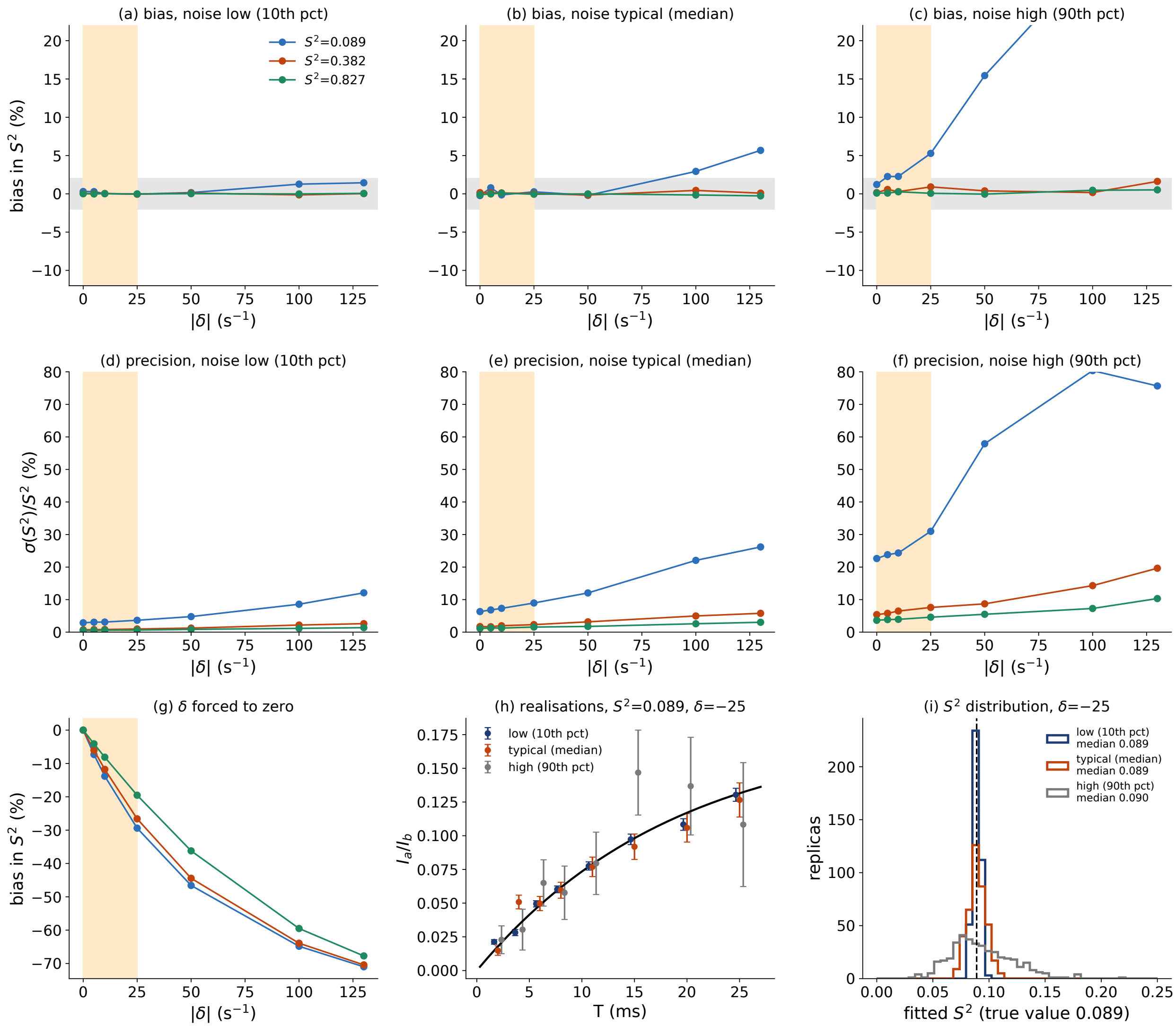

(a) bias, noise low (10th pct)
(b) bias, noise typical (median)
(c) bias, noise high (90th pct)
S^2=0.089
S^2=0.382
S^2=0.827
bias in S^2 (%)
$|\delta|$ (s^{-1})
(d) precision, noise low (10th pct)
(e) precision, noise typical (median)
(f) precision, noise high (90th pct)
$\sigma(S^2)/S^2$ (%)
(g) δ forced to zero
(h) realisations, S^2=0.089, δ=−25
low (10th pct)
typical (median)
high (90th pct)
I_a/I_b
T (ms)
(i) S^2 distribution, δ=−25
low (10th pct) median 0.089
typical (median) median 0.089
high (90th pct) median 0.090
replicas
fitted S^2 (true value 0.089)

Figure S23: (Preceding page.) **Effect of the external-spin term δ on the fitted order parameter, evaluated at three experimentally realistic noise levels.** Triple-quantum buildup curves were simulated with Eq. 1 at the minimum, mean and maximum order parameters of the data set ($S^2_{\text{axis}} = 0.089$, 0.382 and 0.827; blue, orange and green), using the experimental relaxation delays (2, 4, 6, 8, 11, 15, 20 and 25 ms), $\tau_c = 39.0$ ns, $r_{\text{HH}} = 1.813$ Å and δ between 0 and -130 s^{-1}. Noise was drawn from the measured per-delay uncertainties of the 90 methyl groups, taken at their 10th, 50th and 90th percentile (mean over delays 0.0031, 0.0075 and 0.0249), so that the three columns bracket the real data set rather than describing a typical member of it. Each point is the mean over 400 replicates, refitted with both S^2_{axis} and δ free; orange shading marks the range of δ attainable from external protons on structural grounds ($|\delta| \lesssim 25$ s^{-1}). **(a–c)** Relative bias in the fitted S^2_{axis}; grey band, ±2%. Within the structurally plausible range the bias remains small at every noise level and every order parameter: at $\delta = -5$ s^{-1} it is +0.3%, +0.8% and +2.3% for the most mobile methyl ($S^2_{\text{axis}} = 0.089$) at low, typical and high noise, and below 1% in all other cases. A substantial bias appears only when low S^2_{axis}, high noise and large $|\delta|$ coincide (+5.3% at $\delta = -25$ and +15% at $\delta = -50$ s^{-1} in panel c). **(d–f)** Relative precision of the same fits. In contrast to the bias, the precision degrades strongly with both $|\delta|$ and the noise level, and the effect is concentrated at low S^2_{axis}: at $\delta = -25$ s^{-1}, $\sigma(S^2)/S^2$ is 3.6%, 9.0% and 31% across the three columns for $S^2_{\text{axis}} = 0.089$, against 0.6% to 4.6% for $S^2_{\text{axis}} = 0.827$. For the noisiest methyls the relative uncertainty approaches 80% at large $|\delta|$ (panel f), i.e. the order parameter is effectively undetermined. **(g)** Systematic bias incurred if δ is instead constrained to zero, i.e. if external protons are neglected. This is a deterministic misfit and was therefore evaluated on noiseless data: -4 to -7% already at $\delta = -5$ s^{-1}, -20 to -29% at -25 s^{-1}, and approximately -70% at -130 s^{-1}. Fitting δ is thus clearly preferable to ignoring it. **(h)** One noisy realization at each noise level for the most vulnerable case ($S^2_{\text{axis}} = 0.089$, $\delta = -25$ s^{-1}); points are offset horizontally for clarity, solid line is the noiseless curve. **(i)** Distribution of the 400 fitted S^2_{axis} values for that case; dashed line, the true value. At low and typical noise the distribution is a narrow symmetric peak centered on the input value, whereas at high noise it broadens into a right-skewed distribution whose median (0.090) still coincides with the truth while its mean is displaced upward. The apparent bias in panel (c) therefore reflects distributional skew rather than a systematic shift, which implies that for the noisiest methyls a Monte-Carlo standard deviation understates the true uncertainty and a median with percentile intervals is the more appropriate summary.

S5 Bibliography for supplementary information

(1) Li, J.; Bennett, K. C.; Liu, Y.; Martin, M. V.; Head-Gordon, T. Accurate prediction of chemical shifts for aqueous protein structure on "Real World" data. *Chem. Sci.* **2020**, *11*, 3180–3191, DOI: 10.1039/c9sc06561j.

(2) Sun, H.; Kay, L. E.; Tugarinov, V. An optimized relaxation-based coherence transfer NMR experiment for the measurement of side-chain order in methyl-protonated, highly deuterated proteins. *J. Phys. Chem. B* **2011**, *115*, 14878–14884, DOI: 10.1021/jp209049k.

(3) Lee, D.; Hilty, C.; Wider, G.; Wüthrich, K. Effective rotational correlation times of proteins from NMR relaxation interference. *J. Magn. Reson.* **2006**, *178*, 72–76, DOI: 10.1016/j.jmr.2005.08.014.

(4) Favier, A.; Brutscher, B. NMRlib: user-friendly pulse sequence tools for Bruker NMR spectrometers. *J. Biomol. NMR* **2019**, *73*, 199–211, DOI: 10.1007/s10858-019-00249-1.

(5) Yao, L.; Grishaev, A.; Cornilescu, G.; Bax, A. Site-specific backbone amide (15)N chemical shift anisotropy tensors in a small protein from liquid crystal and cross-correlated relaxation measurements. *J. Am. Chem. Soc.* **2010**, *132*, 4295–4309, DOI: 10.1021/ja910186u.

(6) Nucci, N. V.; Marques, B. S.; Bédard, S.; Dogan, J.; Gledhill Jr, J. M.; Moorman, V. R.; Peterson, R. W.; Valentine, K. G.; Wand, A. L.; Wand, A. J. Optimization of NMR spectroscopy of encapsulated proteins dissolved in low viscosity fluids. *J. Biomol NMR* **2011**, *50*, 421–430, DOI: 10.1007/s10858-011-9528-y.

(7) Garcia De la Torre, J.; Huertas, M.; Carrasco HYDRONMR: prediction of NMR relaxation of globular proteins from atomic-level structures and hydrodynamic calculations. *J. Magn. Reson.* **2000**, *147*, 138–146, DOI: 10.1016/S1090-7807(00)00105-0.

(8) Woessner, D. E. Nuclear spin relaxation in ellipsoids undergoing rotational Brownian motion. *J. Chem. Phys.* **1962**, *37*, 647–654, DOI: 10.1063/1.1701390.

(9) Ghose, R.; Fushman, D.; Cowburn, D. Determination of the rotational diffusion tensor of macromolecules in solution from NMR relaxation data with a combination of exact and approximate methods—application to the determination of interdomain orientation in multidomain proteins. *J. Magn. Reson.* **2001**, *149*, 204–217, DOI: 10.1006/jmre.2001.2295.

(10) Mitternacht, S. FreeSASA: An open source C library for solvent accessible surface area calculations. *F1000Research* **2016**, *5*, 189, DOI: 10.12688/f1000research.7931.1.

(11) Halle, B. Flexibility and packing in proteins. *Proc. Natl. Acad. Sci. U. S. A.* **2002**, *99*, 1274–1279, DOI: 10.1073/pnas.032522499.

(12) Ming, D.; Brüschweiler, R. Prediction of methyl-side chain dynamics in proteins. *J. Biomol. NMR* **2004**, *29*, 363–368, DOI: 10.1023/B:JNMR.0000032612.70767.35.

(13) Zhang, F.; Brüschweiler, R. Contact model for the prediction of NMR N–H order parameters in globular proteins. *J. Am. Chem. Soc.* **2002**, *124*, 12654–12655, DOI: 10.1021/ja027847a.

(14) Roche, J.; Girard, E.; Mas, C.; Madern, D. The archaeal LDH-like malate dehydrogenase from Ignicoccus islandicus displays dual substrate recognition, hidden allostery and a non-canonical tetrameric oligomeric organization. *J. Struct. Biol.* **2019**, *208*, 7–17, DOI: 10.1016/j.jsb.2019.07.006.

(15) Shen, Y.; Bax, A. Protein backbone and sidechain torsion angles predicted from NMR chemical shifts using artificial neural networks. *J. Biomol. NMR* **2013**, *56*, 227–241, DOI: 10.1007/s10858-013-9741-y.

(16) Schmidt, E.; Güntert, P. A new algorithm for reliable and general NMR resonance assignment. *J. Am. Chem. Soc.* **2012**, *134*, 12817–12829, DOI: 10.1021/ja306039u.

(17) Guo, C.; Tugarinov, V. Identification of HN-methyl NOEs in large proteins using simultaneous amide-methyl TROSY-based detection. *J. Biomol. NMR* **2009**, *43*, 21–30, DOI: 10.1007/s10858-008-9285-8.

(18) Knödlstorfer, S.; Toscano, G.; Ptaszek, A. L.; Kontaxis, G.; Napoli, F.; Schneider, J.; Maier, K.; Kapitonova, A.; Lichtenecker, R. J.; Schanda, P.; Konrat, R. A Novel HMBC-CC-HMQC NMR Strategy for Methyl Assignment Using Triple-^{13}C-Labeled α-Ketoisovalerate Integrated with UCBShift 2.0. *J. Mol. Biol.* **2025**, *437*, 169465, DOI: 10.1016/j.jmb.2025.169465.

(19) Kerfah, R.; Hamelin, O.; Boisbouvier, J.; Marion, D. CH_3-specific NMR assignment of alanine, isoleucine, leucine and valine methyl groups in high molecular weight proteins using a single sample. *J. Biomol. NMR* **2015**, *63*, 389–402, DOI: 10.1007/s10858-015-9998-4.

(20) Bax, A.; Clore, G. M.; Gronenborn, A. M. ^{1}H-^{1}H correlation via isotropic mixing of ^{13}C magnetization, a new three-dimensional approach for assigning ^{1}H and ^{13}C spectra of ^{13}C-enriched proteins. *J. Magn. Reson.* **1990**, *88*, 425–431, DOI: 10.1016/0022-2364(90)90101-4.

(21) Andreas, L. B. et al. Structure of fully protonated proteins by proton-detected magic-angle spinning NMR. *Proc. Natl. Acad. Sci. U. S. A.* **2016**, *113*, 9187–9192, DOI: 10.1073/pnas.1602248113.

(22) Agarwal, V.; Reif, B. Residual methyl protonation in perdeuterated proteins for multi-dimensional correlation experiments in MAS solid-state NMR spectroscopy. *J. Magn. Reson.* **2008**, *194*, 16–24, DOI: 10.1016/j.jmr.2008.07.004.

(23) Shaka, A.; Lee, C.; Pines, A. Iterative schemes for bilinear operators; application to spin decoupling. *J. Magn. Reson.* **1988**, *77*, 274–293, DOI: 10.1016/0022-2364(88)90146-3.

(24) Luz, Z.; Meiboom, S. Nuclear magnetic resonance study of the protolysis of trimethylammonium ion in aqueous solution—order of the reaction with respect to solvent. *J. Chem. Phys.* **1963**, *39*, 366–370, DOI: 10.1063/1.1734254.

(25) Hansen, D. F.; Kay, L. E. Determining Valine Side-Chain Rotamer Conformations in Proteins from Methyl 13C Chemical Shifts: Application to the 360 kDa Half-Proteasome. *J. Am. Chem. Soc.* **2011**, *133*, 1–10, DOI: 10.1021/ja205482p.

(26) Hansen, D. F.; Neudecker, P.; Kay, L. E. Determination of Isoleucine Side-Chain Conformations in Ground and Excited States of Proteins from Chemical Shifts. *J. Am. Chem. Soc.* **2010**, *132*, 7589–7591, DOI: 10.1021/ja102090z.

(27) Hansen, D. F.; Neudecker, P.; Vallurupalli, P.; Mulder, F. A. A.; Kay, L. E. Determination of Leu Side-Chain Conformations in Excited Protein States by NMR Relaxation Dispersion. *J. Am. Chem. Soc.* **2010**, *132*, 42–43, DOI: 10.1021/ja909294n.

(28) Mulder, F. A. A. Leucine Side-Chain Conformation and Dynamics in Proteins from ^{13}C NMR Chemical Shifts. *ChemBioChem* **2009**, *10*, 1477–1479, DOI: 10.1002/cbic.200900086.

(29) Waudby, C. A.; Ramos, A.; Cabrita, L. D.; Christodoulou, J. Two-dimensional NMR lineshape analysis. *Sci. Rep.* **2016**, *6*, 24826, DOI: 10.1038/srep24826.
